# Supramolecular Behaviour of Heterochiral Dipeptides With Ile

**DOI:** 10.1002/psc.70121

**Published:** 2026-09-02

**Authors:** Erica Scarel, Paola Alletto, Simone Adorinni, Fabio Fazio, Giovanni Pierri, Consiglia Tedesco, Silvia Marchesan

**Affiliations:** ^1^ Department of Chemical and Pharmaceutical Sciences University of Trieste Trieste Italy; ^2^ Department of Chemistry and Biology “A. Zambelli” University of Salerno Fisciano Italy

## Abstract

This study describes the supramolecular behaviour and gelation of heterochiral, aliphatic dipeptides d‐Ile‐l‐Xaa (Xaa = Ala, Val, Leu or Ile) in various solvents and buffered water at neutral pH. In the case of metastable gels, the consequent rapid transition towards crystals enabled X‐ray diffraction analysis to unveil the dipeptides' packing modes in amphipathic layers.

## Graphical abstract

This study describes the supramolecular behaviour and gelation of heterochiral, aliphatic dipeptides d‐Ile‐l‐Xaa (Xaa = Ala, Val, Leu or Ile) in various solvents and buffered water at neutral pH. Metastable gels rapidly crystallised. X‐ray diffraction analysis was conducted to unveil the dipeptides' packing modes in amphipathic layers.

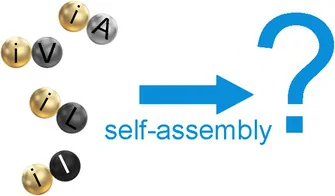

## 1 Introduction

Short peptides are versatile building blocks for green self‐assembled materials. They offer many advantages, such as their ease of preparation and scale‐up, great chemical diversity to encode diverse functions, cytocompatibility and biodegradability. Since the discovery of minimalistic self‐assembling motifs, such as diphenylalanine [1], the interest in this research area has been growing steadily [2]. The applications of these materials span over a wide range of fields from medicine [3, 4, 5, 6] to electronics [7, 8, 9, 10]. Despite the small size of these biomolecules, a vast number of morphologies have been discovered. Short peptides can organise in nanoparticles, nanotubes, fibres, nanobelts and nanoribbons thanks to their amphiphilicity and their ability to form hydrogen bond networks [11].

In particular, porous crystalline nanotubes formed by homochiral dipeptides have been intensively studied through X‐ray crystallography by Prof. Görbitz, revealing the influence of the different alkyl side chains in hydrophobic amino acids (i.e., Ala, Val, Leu, Ile and Phe) on the pore diameter and surface polarity [12]. Porous dipeptide materials of this kind are interesting for technological applications, for instance, through their ability to trap gaseous guest molecules (e.g., CO_2_, H_2_, CH_4_, O_2_, N_2_ and Xe) [13, 14]. Given these interesting findings for the homochiral dipeptides, the following question arises: How different will the corresponding heterochiral superstructures be? To answer this query, our group started the mapping of heterochiral hydrophobic dipeptides, finding that in some cases, such as Phe‐Val and Phe‐Ile, heterochirality enabled the formation of water‐filled nanotubes while homochirality did not; in other cases, such as Phe‐Leu, the opposite was found; lastly, in the case of Phe‐Phe, nanotubes formed analogously for homochiral and heterochiral analogues, although the stereoconfiguration dictated the modes of their hierarchical association into fibrils and fibres [15]. Furthermore, Nle‐Phe and Phe‐Nle were recently reported to form nanotubes too [16], demonstrating that this approach can be successfully extended to sequences with non‐natural amino acids bearing linear side chains, although with differing results based on chirality. Indeed, the rationalisation of these findings is far from trivial, and a complete mapping of the supramolecular behaviour of heterochiral, hydrophobic dipeptides is an important milestone to allow for a thorough comparison with the homochiral sequences. To date, the self‐assembly behaviour of heterochiral dipeptides arising from all possible combinations of aliphatic amino acids featuring Ile is still unreported. In particular, hydrophobic l‐dipeptides with N‐terminal Ile are particularly interesting, being the only ones able to form both hydrophobic (Ile‐Ala and Ile‐Val) [17] and hydrophilic (Ile‐Leu) [18] nanotubes. For this reason, this study focusses on their heterochiral analogues d‐Ile‐l‐Xaa (Xaa = Ala, Val, Leu or Ile) and their ability to fibrillate, gel or crystallise in aprotic polar organic solvents. The single‐crystal X‐ray diffraction (XRD) of these four peptides revealed a different packing compared to their homochiral analogues.

## 2 Materials and Methods

### 2.1 Materials and General Methods

All reagents and solvents were purchased from Merck (Milan, Italy) and were utilised as provided, except for the homochiral dipeptides that were bought from SynPeptides (China) and further purified by HPLC as described further below. 2‐Chlorotrityl chloride resin was sourced from GL Biochem (Shanghai, China). All buffers and aqueous solutions were prepared with pure water that was obtained from a Millipore MilliQ‐system RiOs/Origin (St. Louis, MS, USA) with a resistivity > 18.2 MΩ·cm at 25°C. ^1^H and ^13^C NMR spectra were recorded on a Varian Innova spectrometer at 400 and 100 MHz, respectively, while LC–MS data were obtained with an Agilent 6120 Infinity.

### 2.2 Dipeptide Synthesis and Purification

The dipeptides were prepared by solid‐phase peptide synthesis, using Fmoc‐protection strategy and 2‐chlorotrityl chloride resin, using a standard protocol, followed by reversed‐phase HPLC purification and freeze‐drying [19]. ^1^H and ^13^C NMR spectra and ESI‐MS spectra confirmed products identity and purity (Supporting Information). LC–MS analyses were performed on all the dipeptides using an analytical Agilent 6120 Infinity system (Kinetex EVO C18, 5 μm, 100 Å, 250 × 4.6 mm, Phenomenex) with a quadrupole ESI‐MS detector, by dissolving the dipeptides in water with 0.05% formic acid and then by employing the following gradient using a binary mixture of water and MeCN with 0.05% formic acid: *t* = 0–3 min, 2% MeCN; *t* = 25 min, 50% MeCN; *t* = 36 min, 95% MeCN; *t* = 38 min, 95% MeCN.

### 2.3 Self‐Assembly Tests

The dipeptides were dissolved in glass vials at increasing concentrations up to 100 mM in 0.1 M PBS with mild heating in an oil bath at 80°C, followed by allowing the vial to cool down at room temperature. Alternatively, they were dissolved in an alkaline sodium phosphate (0.1 M) solution at pH 11.8, followed by dilution with an equal volume of a mildly acidic sodium phosphate solution (0.1 M) at pH 5.8, to reach a final pH 7.3.

### 2.4 Circular Dichroism (CD)

CD spectra were recorded on freshly prepared samples (1 mM) in quartz cells (1 mm) in MilliQ water. The temperature was set at 25°C on a Jasco J‐815 with a scanning rate of 50 nm/min and a set resolution of 1 nm.

### 2.5 Crystallisation

The dipeptide crystals were obtained as described in the text from the self‐assembly tests. Details of single‐crystal XRD performed at the XRD1 beamline at Elettra Synchrotron (Italy) can be found in the S1.

### 2.6 Rheology

d‐Ile‐l‐Ile hydrogel was freshly prepared in situ on a 20‐mm‐wide steel plate with parallel geometry of a Malvern Kinexus Ultra Plus Rheometer (Alfatest, Milan, Italy) equipped with a Peltier temperature controller. d‐Ile‐l‐Ile (200 mM) was dissolved in a glass vial in 0.1 M PBS with mild heating in an oil bath at 80°C, followed by allowing the peptide to cool down at room temperature on the wide steel plate. Time sweeps were performed at 1 Hz and 1 Pa at 25°C. Frequency sweeps were performed at 1 Pa and 25°C. Stress sweeps were registered at 1 Hz and 25°C.

## 3 Results and Discussion

### 3.1 Peptide Synthesis, Purification and Spectroscopic Characterisation

The four dipeptide sequences (Table 1) were synthesised in solid phase using the standard Fmoc‐protection strategy protocol, and then, they were purified by reversed‐phase HPLC. However, this workup was not suitable for d‐Ile‐l‐Ala that eluted too quickly, due to its higher hydrophilicity. For this reason, it was purified by precipitation in diethyl ether and Büchner filtration. LC–MS analyses of all the heterochiral dipeptides revealed different retention times (Rt), which can be considered as a crude measure of average hydrophobicity [21], with an overall trend that was consistent with the corresponding log*P* values calculated using the ChemDraw software (Table 1). Interestingly, the analogous homochiral stereoisomers were more hydrophilic, confirming that heterochirality is a useful strategy to increase hydrophobicity to favour self‐assembly in polar media [22, 23]. The dipeptides' identity and purity were confirmed by LC–MS, ^1^H NMR and ^13^C NMR (see the Supporting Information).

**TABLE 1:** Hydrophobicity of the dipeptides.

Dipeptide	HPLC Rt (min)	log*P* ^a^	log*P* ^b^
d‐Ile‐l‐Ala	8.8	−0.25	−0.39
d‐Ile‐l‐Val	13.6	0.64	0.21
d‐Ile‐l‐Leu	15.4	0.98	0.47
d‐Ile‐l‐Ile	15.3	1.05	0.66
l‐Ile‐l‐Ala	3.2	−0.25	−0.39
l‐Ile‐l‐Val	6.3	0.64	0.21
l‐Ile‐l‐Leu	11.7	0.98	0.47
l‐Ile‐l‐Ile	10.7	1.05	0.66

^a^ Calculated using ChemDraw Professional 15.0.0.106.

^b^ Calculated using Swiss ADME (www.SwissADME.ch) [20].

**FIGURE 1:**
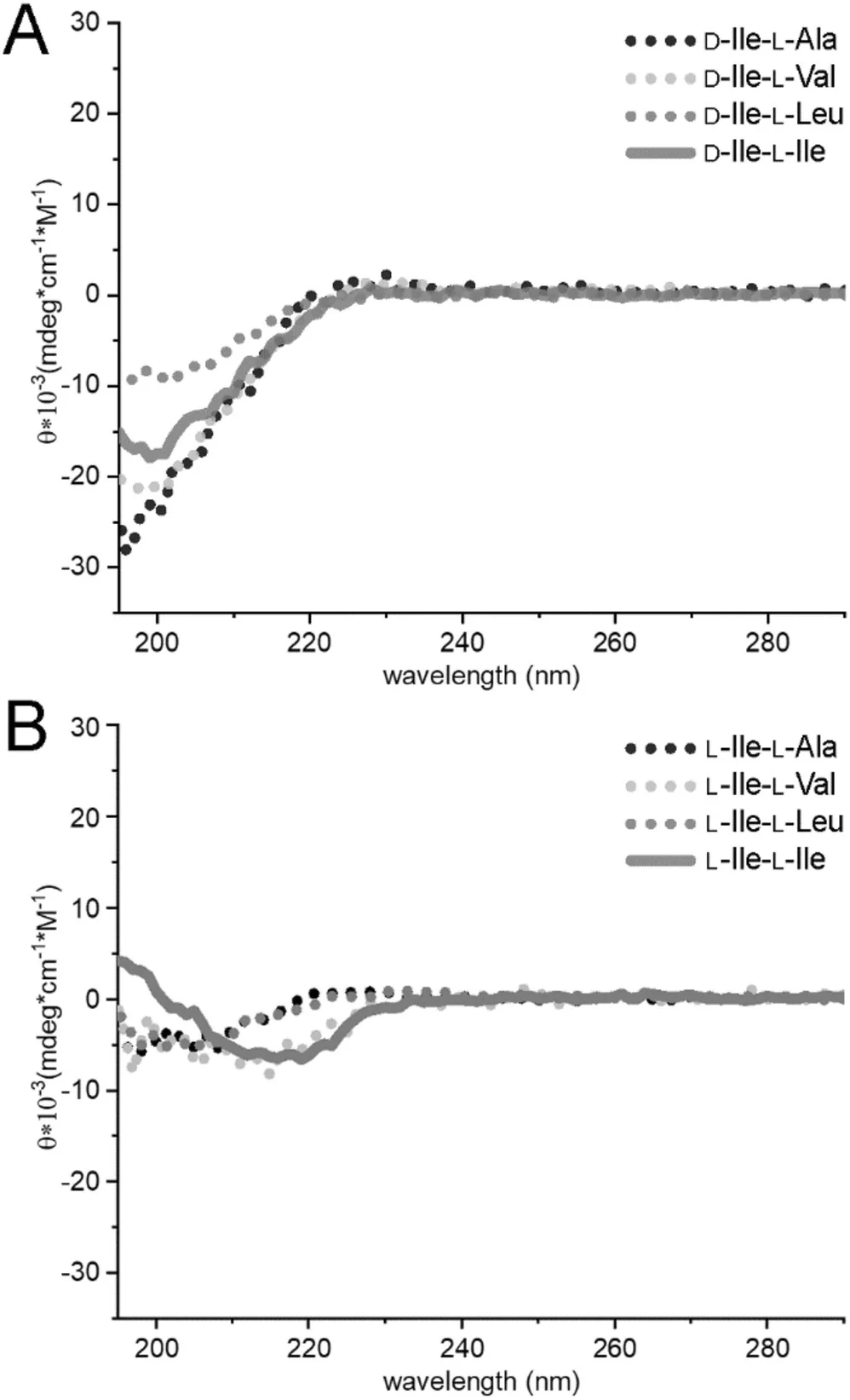
CD spectra of (A) heterochiral and (B) homochiral dipeptides at 1 mM in water, pH 7.0.

All four heterochiral dipeptides were characterised by CD spectroscopy and compared with the corresponding homochiral stereoisomers (Figure 1A). All four d‐Ile‐l‐Xaa peptides shared the same CD signal features highlighting a similar behaviour in water. Previous studies on heterochiral aliphatic dipeptides Leu‐Xaa (Xaa = Ala, Val, Ile or Leu) showed that having a d‐amino acid at the C‐terminus led to CD spectra typical of d‐peptides [19]. Therefore, the enantiomers with a d‐amino acid at the N‐terminus, as in this case, will have mirror‐imaged spectra. Indeed, all four compounds displayed a negative minimum at 200–210 nm and a weakly positive maximum at 220–230 nm, as expected. Such spectral features are reminiscent of l‐peptide random coils, analogously to what was found for the heterochiral Leu‐Xaa aliphatic series [19]. Interestingly, the homochiral analogues (Figure 1B) displayed CD spectra with significantly attenuated intensity of the signals; furthermore, while Ile‐Ala and Ile‐Val revealed the same spectroscopic features of their heterochiral analogues, Ile‐Leu and Ile‐Ile displayed the negative minimum shifted to 210–220 nm range. CD spectra were acquired also at higher concentration (50 mM) and are reported in Figures S33 and S34.

### 3.2 Self‐Assembly and Crystallisation

**FIGURE 2:**
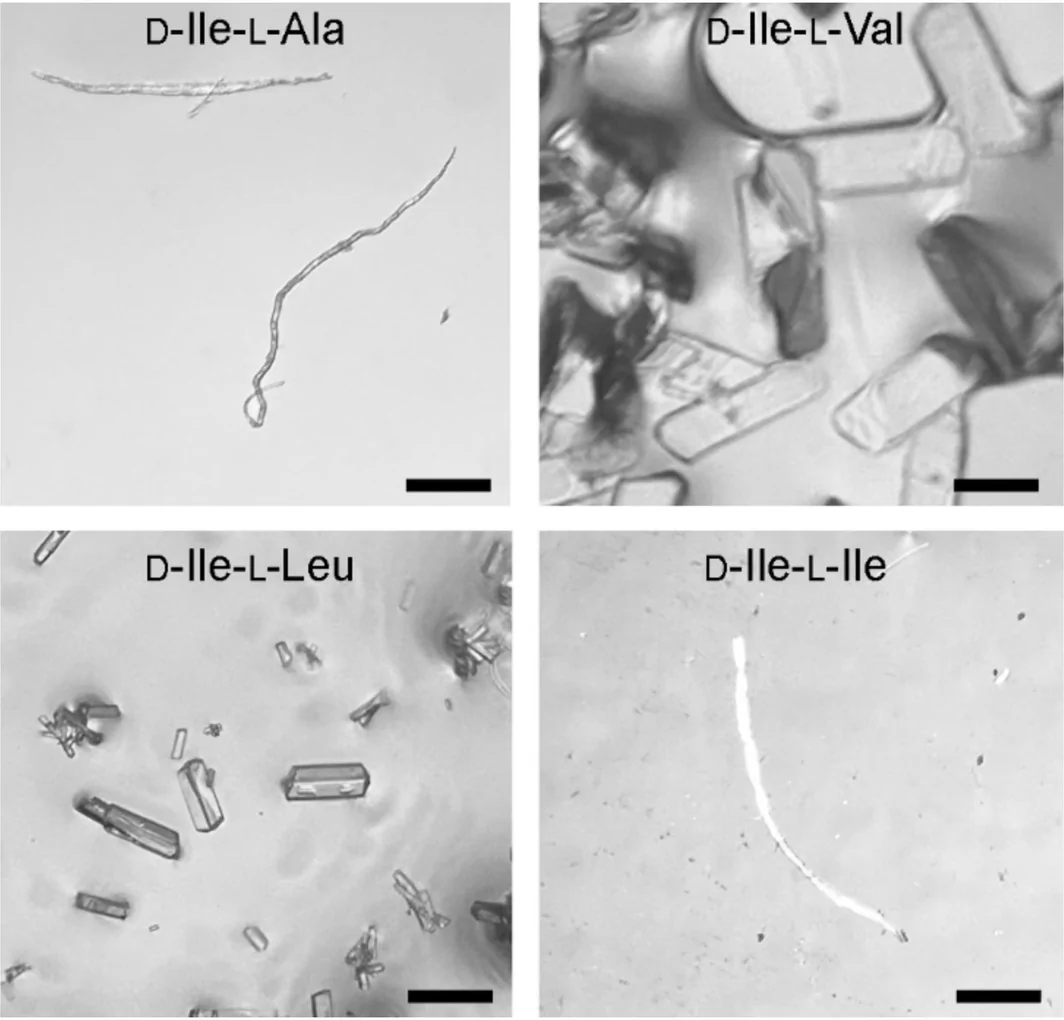
Photographs of the heterochiral peptide solutions observed under the optical microscope through a polarised filter (100 mM, 0.1 M PBS). Scale bar = 200 μm.

**FIGURE 3:**
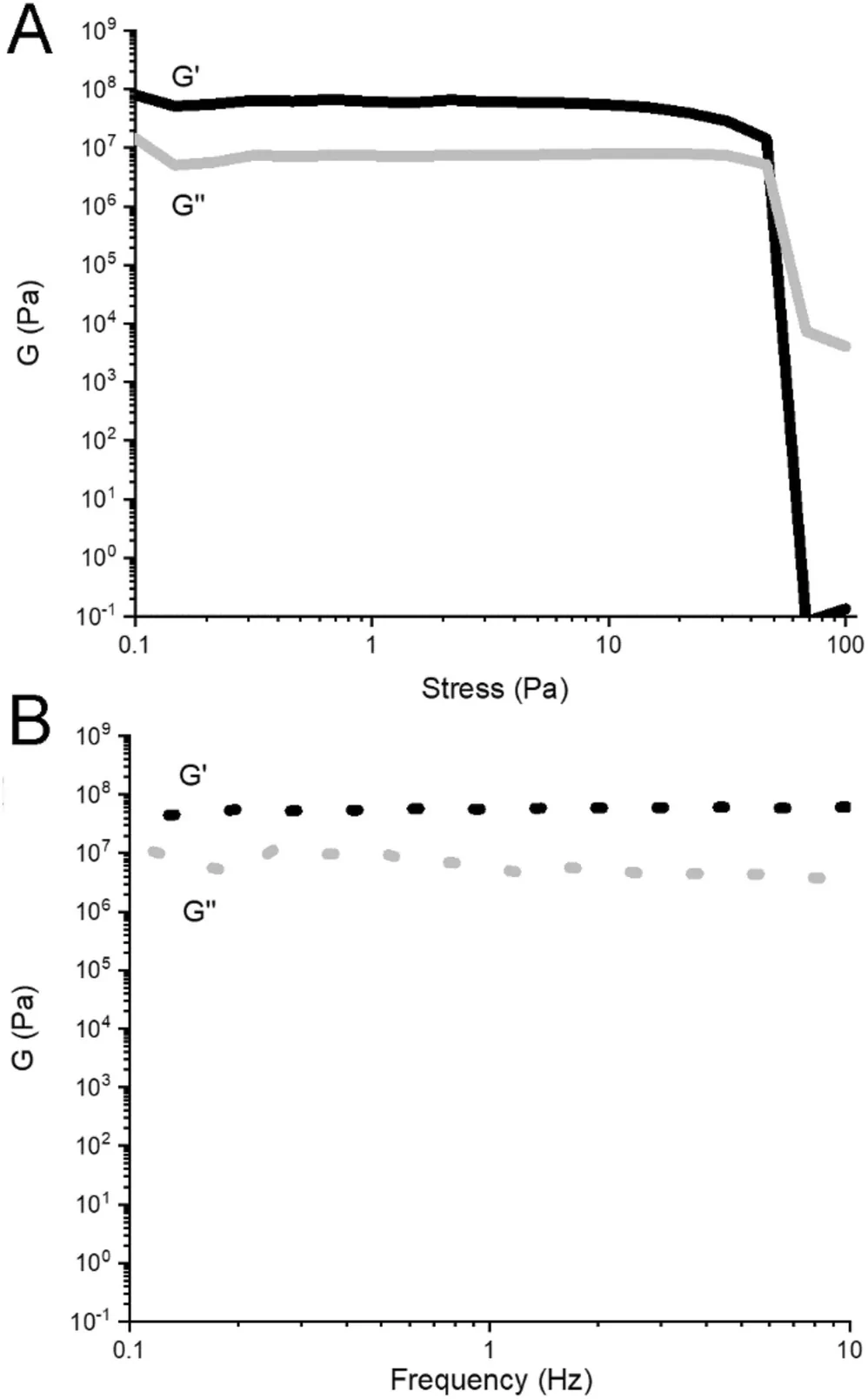
(A) Stress sweep and (B) frequency sweep of d‐Ile‐l‐Ile hydrogel.

The four heterochiral dipeptides were tested for self‐assembly in aqueous and organic solvents. Firstly, we used an established protocol to trigger hydrogelation of hydrophobic dipeptides using a pH switch, from alkaline (pH 11.8) to dissolve the anionic form, to neutral pH, at which the N‐terminus is protonated to yield the zwitterion that can engage in salt bridges for self‐assembly [24]. Under these conditions, no gels formed, only few short fibrils were observed. Unfortunately, however, at these conditions, no gels formed, but a few short fibrils (Table 2). Hydrogelation was tested also at higher ionic strength in phosphate buffered saline (PBS, 0.1 M), because these conditions successfully led to hydrogelation of d‐Phe‐l‐Ile [22]. Under these conditions, all the dipeptides formed rare instances of short fibrils, except for d‐Ile‐l‐Leu and d‐Ile‐l‐Val that formed microcrystals (Figure 2). Further increase of their concentration up to 200 mM yielded a hydrogel solely in the case of d‐Ile‐l‐Ile, as confirmed by rheometry (Figure 3 and the Supporting Information). Because gelation occurred immediately, it was not possible to monitor the sol‐to‐gel transition in the rheometer. Noteworthily, the stress sweep (Figure 3A) revealed a linear viscoelastic range up to 10 Pa and a gel‐to‐sol transition occurring at ~50 Pa. The frequency sweep (Figure 3B) confirmed the hydrogel stability. The corresponding xerogel was also analysed by ATR‐IR (see Figure S35) and showed the typical amide I signal at 1634 cm^−1^ of amyloids, as well as a prominent signal in the range where β‐turns are found for longer peptides (1676–1686 cm^−1^), as observed for fibrillating Nle‐Phe and Phe‐Nle dipeptides [16]. Additionally, the signal at 1725 cm^−1^ of the protonated COOH group involved in H‐bonding was visible.

**TABLE 2:** Self‐assembly tests in various solvents at 50 mM.

Dipeptide	0.1‐M PBS	MeCN	THF	DMSO
d‐Ile‐l‐Ala	Rare fibres	Insoluble	Gel^a^, ^b^	Insoluble
d‐Ile‐l‐Val	Crystals	Crystals	Crystals^b^	Fibres
d‐Ile‐l‐Leu	Crystals	Gel^a^	Fibres	Crystals^b^
d‐Ile‐l‐Ile	Fibres	Gel^a^/crystals^b^	Crystals	Fibres

^a^ Organogel formed as metastable phase, which evolved quickly into crystals.

^b^ Crystals collected and of suitable quality for single‐crystal XRD analysis.

For comparison, the four homochiral analogues were also tested under the same conditions described above, and no hydrogelation was observed. Rare instances of short fibres were observed for all the homochiral dipeptides too (see the Supporting Information). In the case of the homochiral dipeptides, the situation remained unchanged even when their concentration was increased to 200 mM, in contrast with the heterochiral d‐Ile‐l‐Ile, which was the only dipeptide of the whole series undergoing hydrogelation.

**FIGURE 4:**
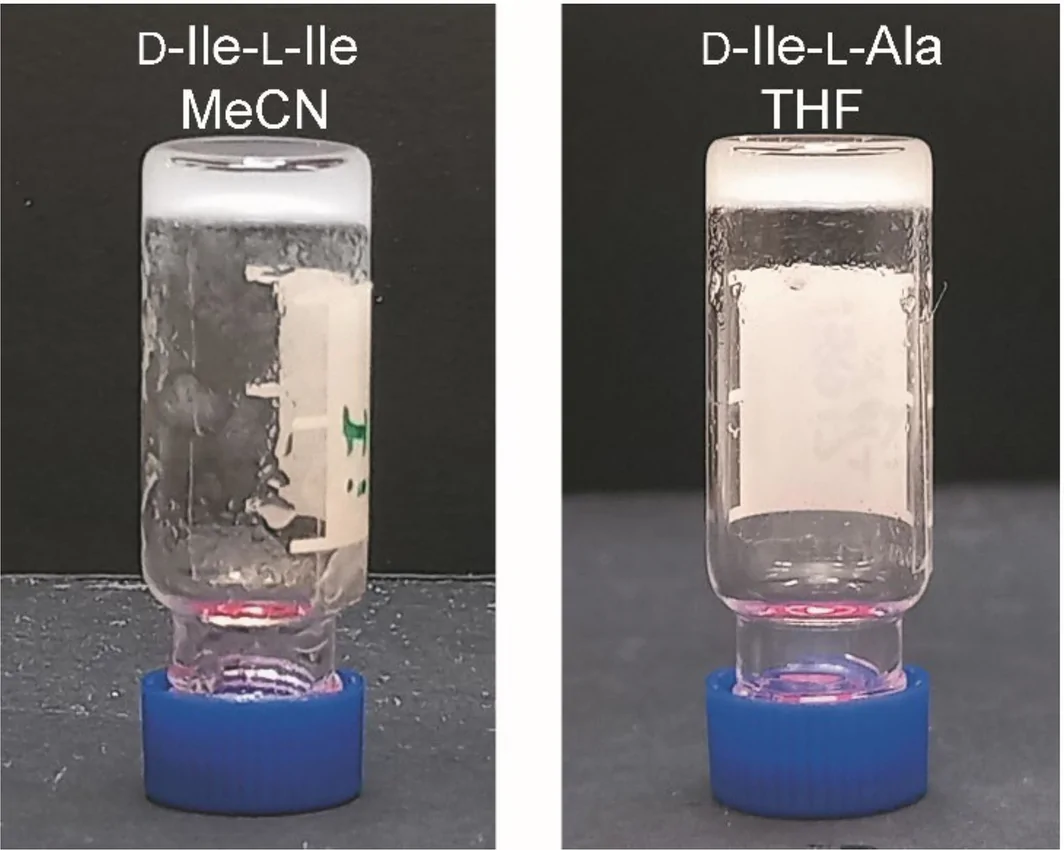
Photographs of the two metastable organogels.

The dipeptides were tested for gelation in organic solvents too (Table 2), which were reported to influence self‐organisation of other dipeptides in the literature. For example, traces of water present in the organic solvent led to a different behaviour in the case of Phe‐Phe [25]. This effect was also observed for d‐Leu‐l‐Xaa (Ala, Val, Leu or Ile) series in MeCN, with hydrogelation occurring with traces of water acting as precipitant [19]. For this reason, MeCN and other polar aprotic organic solvents were tested on the four dipeptides. Peptides were dissolved in the organic solvent as trifluoroacetate salt, and then, the assembly was triggered by the addition of a small amount of NaOH 1 M (2 μL in 100 μL of organic solvent). The addition of the base induces a change in the protonation state of the dipeptide to the zwitterionic species and introduces a quantity of water that is insufficient to solubilise the peptide. As a result, all peptides crystallised, and in two cases, it was possible to observe a metastable transient organogel before the crystallisation (Table 2 and Figure 4), which, however, occurred too rapidly to enable rheological analyses. In particular, d‐Ile‐l‐Ala formed an organogel in tetrahydrofuran (THF), whereas it was insoluble in MeCN and dimethylsulphoxide (DMSO). The organogel evolved quickly into crystals, which proved to be of suitable quality for XRD analysis, revealing a zwitterionic anhydrous polymorph (vide infra). d‐Ile‐l‐Ile showed a similar behaviour in MeCN. In this case, it was possible to slow down the gel‐to‐crystal transition by cooling down the organogel to 5°C. Nevertheless, the organogel collapsed into peptide crystals and liquid solvent within minutes, thus impeding rheological characterisation. Unfortunately, the obtained crystals were too fragile and thin to be investigated by single‐crystal XRD. d‐Ile‐l‐Ile crystallised also in DMSO, quickly forming large crystals composed of the packing of the zwitterionic peptide and DMSO. d‐Ile‐l‐Val and d‐Ile‐l‐Leu were prone to crystallise easily without forming transient organogels. Both peptides crystallised as hydrated zwitterions. It is worth noting that the observed transient organogels were formed only with freshly freeze‐dried dipeptides. Due to their hygroscopicity, peptide samples that were left at room temperature absorbed humidity from the air and were unable to assemble in organogels without previous freeze‐drying. Considering all these observations, it is clear that traces of water can influence the self‐assembling ability of these aliphatic dipeptides.

### 3.3 Single‐Crystal XRD Analysis

**FIGURE 5:**
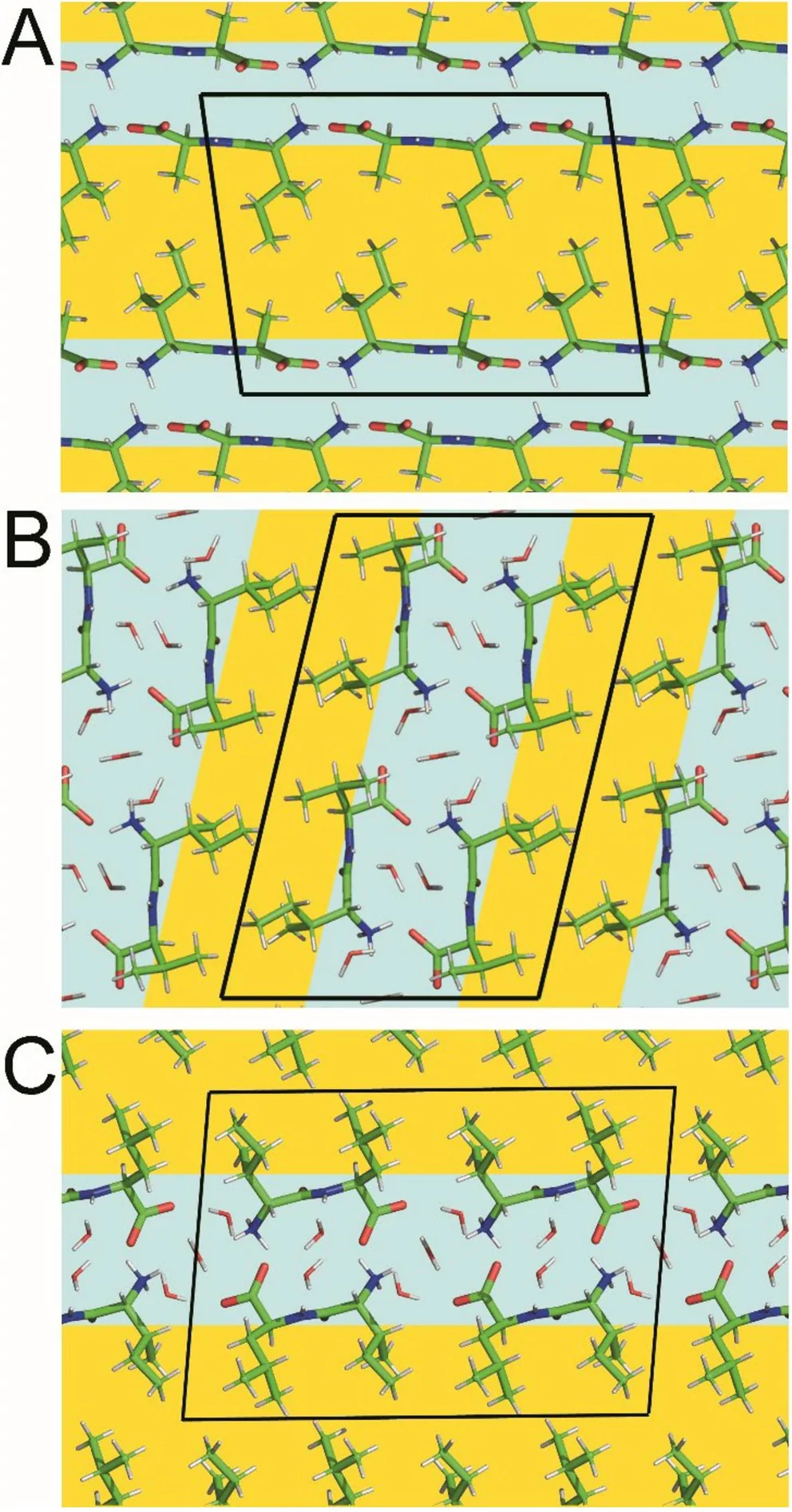
Supramolecular packing in amphipathic layers (hydrophobic regions in yellow and hydrophilic regions in light blue) of the three heterochiral dipeptides bearing Ala (A), Val (B) or Leu (C) as viewed along the *b* crystallographic axis.

All four heterochiral dipeptides produced crystals of suitable quality for XRD analysis (Table 3). Three heterochiral dipeptides (bearing Ala, Val or Leu) crystallised in the monoclinic crystal system within *C*2 space group. The Ala‐bearing dipeptide crystallised in anhydrous form, and the asymmetric unit is composed of one zwitterionic peptide molecule (Figure 5A and the Supporting Information for details). Those bearing either Val or Leu, instead, crystallised with water as cocrystal component with a similar packing (Figure 5B,C and the Supporting Information).

**TABLE 3:** Main features of the crystals analysed by single‐crystal XRD and obtained with the conditions indicated in Table 2.

Sequence	d‐Ile‐l‐Ala	d‐Ile‐l‐Val	d‐Ile‐l‐Leu	d‐Ile‐l‐Ile
CCDC code	2476795	2476794	2477381	2476796
Crystallisation solvent	THF	THF	MeCN	DMSO
Protonation state	Zwitterionic	Zwitterionic	Zwitterionic	Zwitterionic
Cocrystal molecule	/	Water	Water	DMSO
Space group	*C*2	*C*2	*C*2	*P*2_1_
*a* (Å)	16.672(3)	19.228(4)	19.352(4)	5.7010(11)
*b* (Å)	5.1930(10)	6.5710(13)	6.5710(13)	18.209(4)
*c* (Å)	12.480(3)	12.268(3)	13.009(3)	15.351(3)
*β* (°)	97.49(3)	103.15(3)	95.15(3)	93.63(3)

The three heterochiral dipeptides packed in alternating polar/hydrophobic layers that were clearly visible along the *b* crystallographic axis (Figure 5). It is worth noting that the rapid crystallisation of all these dipeptides was triggered by the addition of a small amount of water, after their dissolution in the aprotic organic solvent. However, the dipeptide with Ala first formed an organogel, and then, it crystallised in the anhydrous form. This tendency to form metastable organogels was also observed by the heterochiral Leu‐Xaa series (Ala, Val, Leu and Ile) in MeCN [19]. The increased steric hindrance of Val (or Leu) side chain, relative to Ala, affected the polar region of the crystal, changing the position of the carboxylate and crystallising the peptide as hydrate. Furthermore, both dipeptides (with Val or Leu) share the same conformation in the crystalline state, but the increased steric hindrance of the Leu side chain, relative to Val, affected the *β* angle of the unit cell and the interdigitation of the Ile side chain in the hydrophobic zipper.

The homochiral, analogue sequences studied by Prof. Görbitz revealed a porous packing for all three diastereomers [17]. In particular, l‐Ile‐l‐Ala and l‐Ile‐l‐Val crystallised in the hexagonal crystal system in the space group *P*6_1_ forming hydrophobic channels. For both homochiral peptides, their alkylic side chains are in *r diad* disposition. In this conformation, each side chain is involved in the wall of a different hydrophobic pore. By contrast, heterochirality results in a differing position of one of the two side chains of the analogue stereoisomers, and the corresponding heterochiral dipeptides (as well as the other two studied in this work) were found to prefer the *m diad* disposition. Homochiral l‐Ile‐l‐Leu was also observed in a *m diad* disposition crystallising in the monoclinic crystal system in the *C*2 space group as a hydrate [18]. However, in this case, the dipeptide packed in hydrophilic channels.

**FIGURE 6:**
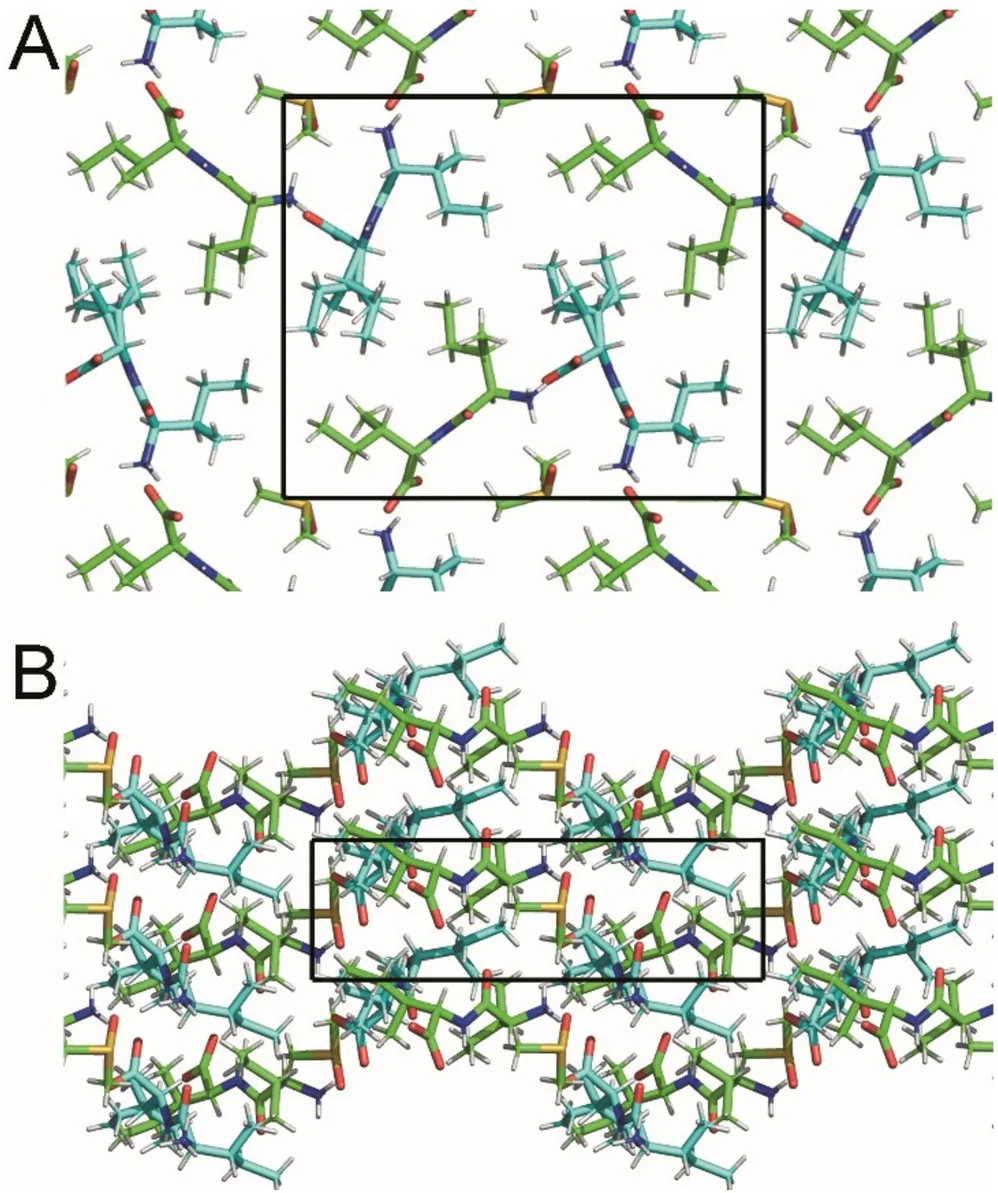
d‐Ile‐l‐Ile crystal packing: (A) as viewed along the *a* axis and (B) along the *c* axis.

Finally, d‐Ile‐l‐Ile, as previously discussed, formed a metastable organogel in acetonitrile. The crystals evolved from the gel but revealed to be too thin and small to be investigated by XRD. For this reason, the peptide was crystallised with the same protocol but in DMSO (as shown in Table 2). The peptide crystallised quickly in large plate‐like crystals that were collected and analysed by single‐crystal XRD. The compound crystallised in the monoclinic crystal system within *P*2_1_ space group as a zwitterion. The asymmetric unit is composed of two peptide molecules and one DMSO molecule (Figure 6). The homochiral diastereomer was characterised by Prof. Görbitz in different protonation states [26]. The homochiral zwitterion crystallised forming hydrophobic columns separated by oval polar regions with the peptide adopting a *r diad* configuration. Unfortunately, the heterochiral d‐Ile‐l‐Ile crystallised with DMSO as cocrystal component, thus not allowing any meaningful comparison with the homochiral hydrate crystal structure or the possible organogel suprastructure obtained in acetonitrile. Indeed, DMSO is a strong hydrogen bond acceptor that significantly disrupts the native peptide backbone hydrogen‐bonding network. The DMSO fills the amphiphilic regions of the crystal acting as hydrogen bond acceptor (oxygen) and interacting with the hydrophobic peptide side chains through the methyl groups (see the Supporting Information). This particular packing was also observed for heterochiral Leu‐Leu and Leu‐Ile dipeptides [19].

We also calculated the *χ*_1_ and *χ*_2_ torsion angles of the isoleucine side chains (Table S2): In all cases, the first d‐Ile residue features a *tt* conformation. As for d‐Ile‐l‐Ile peptide crystals, the second residue features both *g* ^*−*^ *t* and *tt* conformations (with a value of *χ*_1_ close to an energetically unfavoured value of 90° for the chain with occupancy factor of 0.14).

## 4 Conclusions

In conclusion, this work reports the characterisation of the heterochiral dipeptides d‐Ile‐l‐Xaa (Xaa = Ala, Val, Leu or Ile). Heterochirality resulted in an increased hydrophobicity relative to the homochiral analogues, as confirmed by reversed‐phase HPLC. CD spectra revealed similar conformations in water at neutral pH as observed for the analogous series of heterochiral dipeptides with Leu at the N‐terminus, in place of Ile. Self‐assembly tests confirmed the tendency to fibrillate for all the dipeptides in various solvents. In particular, the dipeptides bearing Ala or Ile at the C‐terminus formed metastable gels that rapidly evolved into crystals. Single‐crystal XRD revealed a similar packing mode for all the dipeptides—except for d‐Ile‐l‐Ile—into amphipathic layers, with or without water molecules in the hydrophilic regions. Interestingly, d‐Ile‐l‐Ile was the only dipeptide of the series able to form also hydrogels at neutral pH. Although this dipeptide crystallised in different conditions, the only crystals that were large enough to allow for successful structural elucidation by XRD contained H‐bound DMSO in the structure. Therefore, it is possible that this dipeptide packs in differing modes in other conditions, and we cannot exclude its ability to form porous crystals in the absence of DMSO, thus giving scope for further investigations on its crystallisation. Once the mapping of the crystal structures of the aliphatic dipeptide series is complete, it will be possible to better rationalise their ability to yield nanotubes to enable their rational design with other sequences too. Furthermore, water has been shown to play an important role in the nanomorphologies formed by heterochiral peptides, and its role in these systems certainly deserves further investigation in the future [27].

## Conflicts of Interest

The authors declare no conflicts of interest.

## Supporting information


**Figure S1:** ^1^H NMR (400 MHz, MeOD‐*d*_4_) of d‐Ile‐l‐Ala.
**Figure S2:** H–H COSY (400 MHz, MeOD‐*d*_4_) of d‐Ile‐l‐Ala.
**Figure S3:** ^13^C NMR (100 MHz, MeOD‐*d*_4_) of d‐Ile‐l‐Ala.
**Figure S4:** HSQCAD (100 MHz, MeOD‐*d*_4_) of d‐Ile‐l‐Ala.
**Figure S5:** ESI mass spectrum of d‐Ile‐l‐Ala. Positive (top) and negative mode (bottom).
**Figure S6:** ^1^H NMR (500 MHz, DMSO‐*d*_6_) of d‐Ile‐l‐Val.
**Figure S7:** H–H COSY (500 MHz, DMSO‐*d*_6_) of d‐Ile‐l‐Val.
**Figure S8:** ^13^C NMR (125 MHz, DMSO‐*d*_6_) of d‐Ile‐l‐Val.
**Figure S9:** HSQCAD (125 MHz, DMSO‐*d*_6_) of d‐Ile‐l‐Val.
**Figure S10:** ESI mass spectrum of d‐Ile‐l‐Val. Positive (top) and negative mode (bottom).
**Figure S11:** ^1^H NMR (500 MHz, DMSO‐*d*_6_) of d‐Ile‐l‐Leu.
**Figure S12:** H–H COSY (500 MHz, DMSO‐*d*_6_) of d‐Ile‐l‐Leu.
**Figure S13:** ^13^C NMR (125 MHz, DMSO‐*d*_6_) of d‐Ile‐l‐Leu.
**Figure S14:** HSQCAD (125 MHz, DMSO‐*d*_6_) of d‐Ile‐l‐Leu.
**Figure S15:** ESI mass spectrum of d‐Ile‐l‐Leu. Positive (top) and negative mode (bottom).
**Figure S16:** ^1^H NMR (500 MHz, DMSO‐*d*_6_) of d‐Ile‐l‐Ile.
**Figure S17:** H–H COSY (500 MHz, DMSO‐*d*_6_) of d‐Ile‐l‐Ile.
**Figure S18:** ^13^C NMR (125 MHz, DMSO‐*d*_6_) of d‐Ile‐l‐Ile.
**Figure S19:** HSQCAD (125 MHz, DMSO‐*d*_6_) of d‐Ile‐l‐Ile.
**Figure S20:** ESI mass spectrum of d‐Ile‐l‐Ile. Positive (top) and negative mode (bottom).
**Figure S21:** ^1^H NMR (500 MHz, DMSO‐*d*_6_) of l‐Ile‐l‐Ala.
**Figure S22:** ESI mass spectrum of l‐Ile‐l‐Ala. Positive (top) and negative mode (bottom).
**Figure S23:** ^1^H NMR (500 MHz, DMSO‐*d*_6_) of l‐Ile‐l‐Val.
**Figure S24:** ESI mass spectrum of l‐Ile‐l‐Val. Positive (top) and negative mode (bottom).
**Figure S25:** ^1^H NMR (500 MHz, DMSO‐*d*_6_) of l‐Ile‐l‐Leu.
**Figure S26:** ESI mass spectrum of l‐Ile‐l‐Leu. Positive (top) and negative mode (bottom).
**Figure S27:** ^1^H NMR (500 MHz, DMSO‐*d*_6_) of l‐Ile‐l‐Ile.
**Figure S28:** ESI mass spectrum of l‐Ile‐l‐Ile. Positive (top) and negative mode (bottom).
**Figure S29:** Reversed‐phase HPLC traces of dipeptides.
**Figure S30:** Photographs of self‐assembly tests of heterochiral dipeptides at increasing concentrations in 0.1‐M PBS. *In sodium phosphate buffer 0.1 M (pH trigger method).
**Figure S31:** Photographs and optical microscope images of self‐assembly tests of homochiral dipeptides at increasing concentrations in 0.1‐M PBS. *In sodium phosphate buffer 0.1 M (pH trigger method).
**Figure S32:** Photograph of self‐assembly tests at 200 mM in 0.1‐M PBS.
**Figure S33:** CD spectra of heterochiral dipeptides at 50 mM in MilliQ water at pH 7.
**Figure S34:** CD spectra of homochiral dipeptides at 50 mM in MilliQ water at pH 7.
**Figure S35:** ATR‐IR spectrum of the xerogel of d‐Ile‐l‐Ile at 200 mM in PBS.
**Figure S36:** d‐Ile‐l‐Ala crystal packing: (A) view along the *b* crystallographic axis and (B) view along the *a* crystallographic axis. Peptide is represented as stick model. Green: carbon, blue: nitrogen, red: oxygen, white: hydrogen.
**Figure S37:** d‐Ile‐l‐Ala noncovalent polar interactions. (A) One layer is composed of staggered peptides that interact by head‐to‐tail salt bridges between termini. The amide groups of neighbouring peptides are too distant to engage in hydrogen bond interactions. Side chains are represented as wire model for clarity. (B) Layers are connected in the polar region by more salt bridges between neighbouring termini.
**Figure S38:** d‐Ile‐l‐Val crystal packing: (A) view along the *b* crystallographic axis and (B) view along the *a* crystallographic axis. Peptide is represented as stick model. Green: carbon, blue: nitrogen, red: oxygen, white: hydrogen.
**Figure S39:** d‐Ile‐l‐Val hydrogen bond network. One peptide chain and three water molecules are present in the asymmetric unit. (A) One peptide layer is composed by a staggered peptide packing. Two water molecules (red oxygen) act as a bridge between different peptide units whereas one water molecule (cyan oxygen) is involved in an intramolecular bridge between the termini of the same peptide. This water molecule further interacts as a hydrogen bond donor with the amide carbonyl of the neighbouring peptide layer. (B) The peptide layers are bridged by all the water molecules. Two water molecules (red oxygen) bridge carboxylates and ammonium groups between the peptide layers. The water molecule in the special *C2* position interacts with four different peptide termini in a tetrahedral geometry. The cyan water molecule bridges the carboxylate group present in one layer with the amide carbonyl group of the neighbouring peptide layer. In panel (B), side chains are omitted for clarity.
**Figure S40:** d‐Ile‐l‐Leu crystal packing: (A) view along the *b* crystallographic axis and (B) view along the *a* crystallographic axis. Peptide is represented as stick model. Green: carbon, blue: nitrogen, red: oxygen, white: hydrogen.
**Figure S41:** d‐Ile‐l‐Leu hydrogen bond network. One peptide chain and three water molecules are present in the asymmetric unit. (A) The peptide layer shows a disposition similar to d‐Ile‐l‐Val. Water molecules bridge the polar termini between peptide chains. The cyan water molecule bridge amide groups of neighbouring peptides; thus, there is no direct interaction between different amides. (B) The two peptide layers are bridged by all the water molecules present in the asymmetric unit. The cyan water molecule bridges intramolecularly the N‐ and C‐terminal residue of the peptide. In panel (B), side chains are omitted for clarity.
**Figure S42:** d‐Ile‐l‐Ile crystal packing: (A) view along the *a* crystallographic axis and (B) view along the *c* crystallographic axis. Peptide is represented as stick model. Green, cyan: carbon, blue: nitrogen, red: oxygen, yellow: sulphur, white: hydrogen.
**Figure S43:** d‐Ile‐l‐Ile Hydrogen bond network. (A) Head‐to‐tail salt bridges between peptides. Amide group of the green peptide is isolated, and it is not involved in hydrogen bond. DMSO is omitted for clarity. Side chains are either omitted or represented by wire model clarity. (B) DMSO acts as a hydrogen bond acceptor bridging the two N‐terminal residues of the two independent peptide conformers. The NH amide group of the cyan peptide engages in a hydrogen bond interaction with the carboxylate group of the neighbour cyan peptide.
**Table S1:** Crystallographic data and refinement details.
**Table S2:** Values of *χ*_1_ and *χ*_2_ torsion angles of the isoleucine side chains for heterochiral dipeptides d‐Ile‐l‐Xaa (Xaa = Ala, Val or Ile).

## Data Availability

The data that support the findings of this study are available in the Supporting Information of this article.
