# Supplementary material for: Supramolecular Behaviour of Heterochiral Dipeptides With Ile
Source: J Pept Sci. 2026 Sep 2;32(10):e70121. doi: 10.1002/psc.70121 (PMC13538969; doi:10.1002/psc.70121)
Supplement: Supplementary file 1 [file PSC-32-e70121-s001.pdf]

Supporting Information

Supramolecular behavior of heterochiral dipeptides with Ile

Erica Scarel,[1] Paola Alletto,[1] Simone Adorinni,[1] Fabio Fazio,[1] Giovanni Pierri,[2] Consiglia Tedesco,[2] and Silvia Marchesan*[1]

[1] Dept. of Chemical and Pharmaceutical Sciences, University of Trieste, 34127 Trieste, Italy
[2] Department of Chemistry and Biology "A. Zambelli", University of Salerno, 84084 Fisciano, Italy
smarchesan@units.it

Table of Contents

1. D-Ile-L-Ala spectroscopic data……………………………………………………… 2
2. D-Ile-L-Val spectroscopic data……………………………………………………… 5
3. D-Ile-L-Leu spectroscopic data……………………………………………………… 8
4. D-Ile-L-Ile spectroscopic data………………………………………………………… 11
5. L-Ile-L-Ala spectroscopic data……………………………………………………… 14
6. L-Ile-L-Val spectroscopic data……………………………………………………… 15
7. L-Ile-L-Leu spectroscopic data……………………………………………………… 16
8. L-Ile-L-Ile spectroscopic data………………………………………………………… 18
9. HPLC traces…………………………………………………………………………….. 20
10. Self-assembly tests……………………………………………………………………… 21
11. Single-crystal X-ray diffraction……………………………………………………… 24

1. D-Ile-L-Ala spectroscopic data

^{1}H NMR (400 MHz, MeOD-d_4, TMS) δ (ppm): 4.35 (q, J = 7.2 Hz, 1H, CH_α Ala), 3.67 (d, J = 6.4 Hz, 1H, CH_α Ile), 1.91 (m, 1H, CH_β Ile), 1.60 (m, 1H, $CH_{2\gamma}$), 1.41 (d, J = 7.2 Hz, 3H, CH_3 Ala), 1.23 (m, 1H, $CH_{2\gamma}$ Ile), 1.02 (d, J = 6.8 Hz, 3H, $CH_{3\gamma}$ Ile), 0.98 (dd, J = 8.0 Hz, J = 7.2 Hz, 3H, $CH_{3\delta}$ Ile). ^{13}C NMR (100 MHz, MeOD-d_4, TMS) δ (ppm): 177.4 (1C, COOH), 169.0 (1C, CN), 59.0 (1C, C_α Ile), 50.6 (1C, C_α Ala), 37.8 (1C, C_β Ile), 25.6 (1C, $CH_2\gamma$ Ile), 18.2 (1C, C_β Ala), 15.1 (1C, $CH_3\gamma$ Ile), 11.5 (1C, $CH_{3\delta}$).

In the H–H COSY spectrum it is possible to observe the coupling of CH_α Ala with CH_3 Ala, CH_β Ile with $CH_3\gamma$ Ile, and CH_α Ile with CH_β Ile, as highlighted in the spectrum (Fig. S2). Similarly, the cross-peaks are highlighted in the HSQCAD spectrum (Fig. S4).

MS (ESI): m/z 203.0 [M+H]$^+$, 225.0 [M+Na]$^+$. 201.0 [M-H]$^-$.

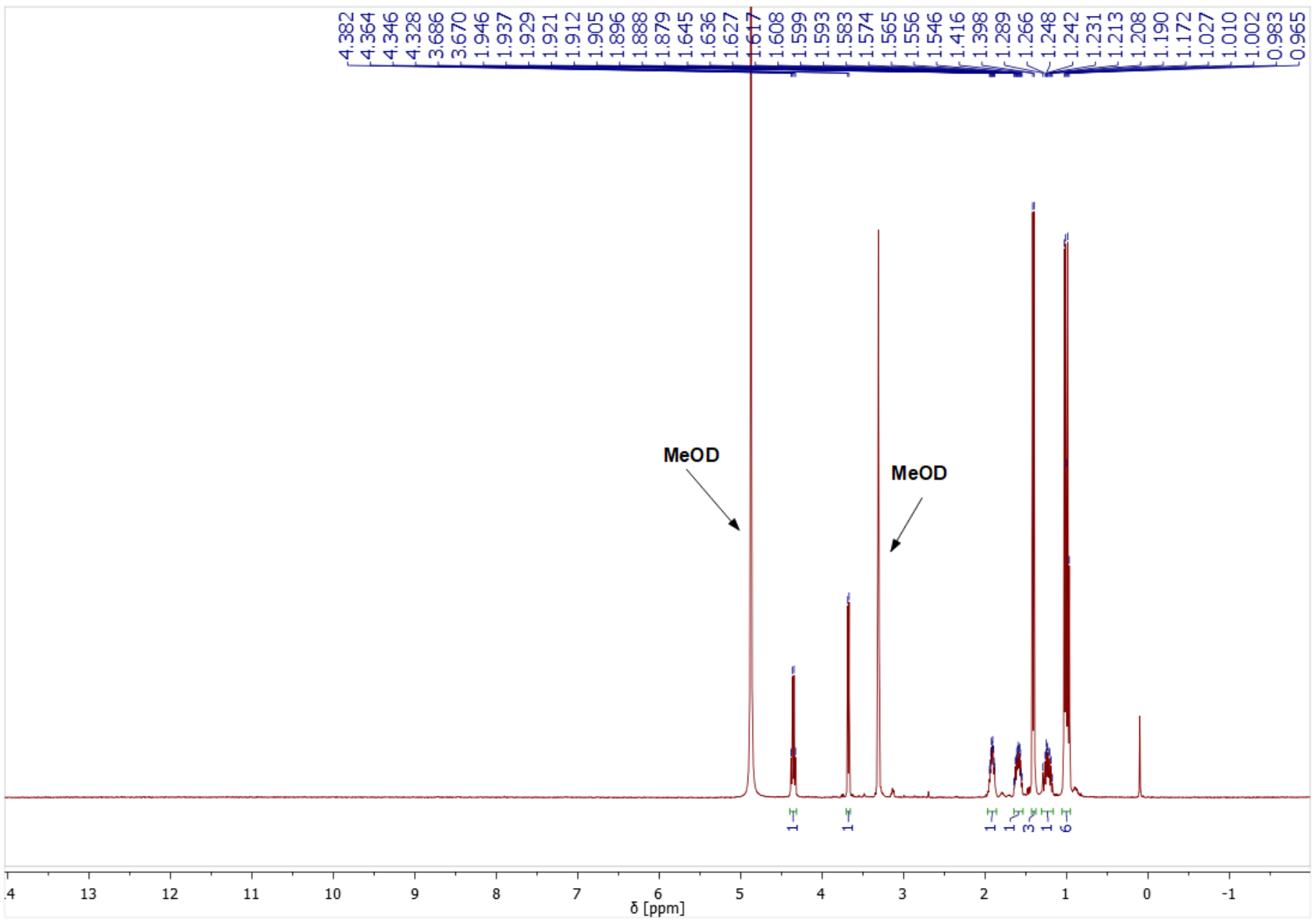

Figure S1: ^{1}H NMR (400 MHz, MeOD-d4) of D-Ile-L-Ala.

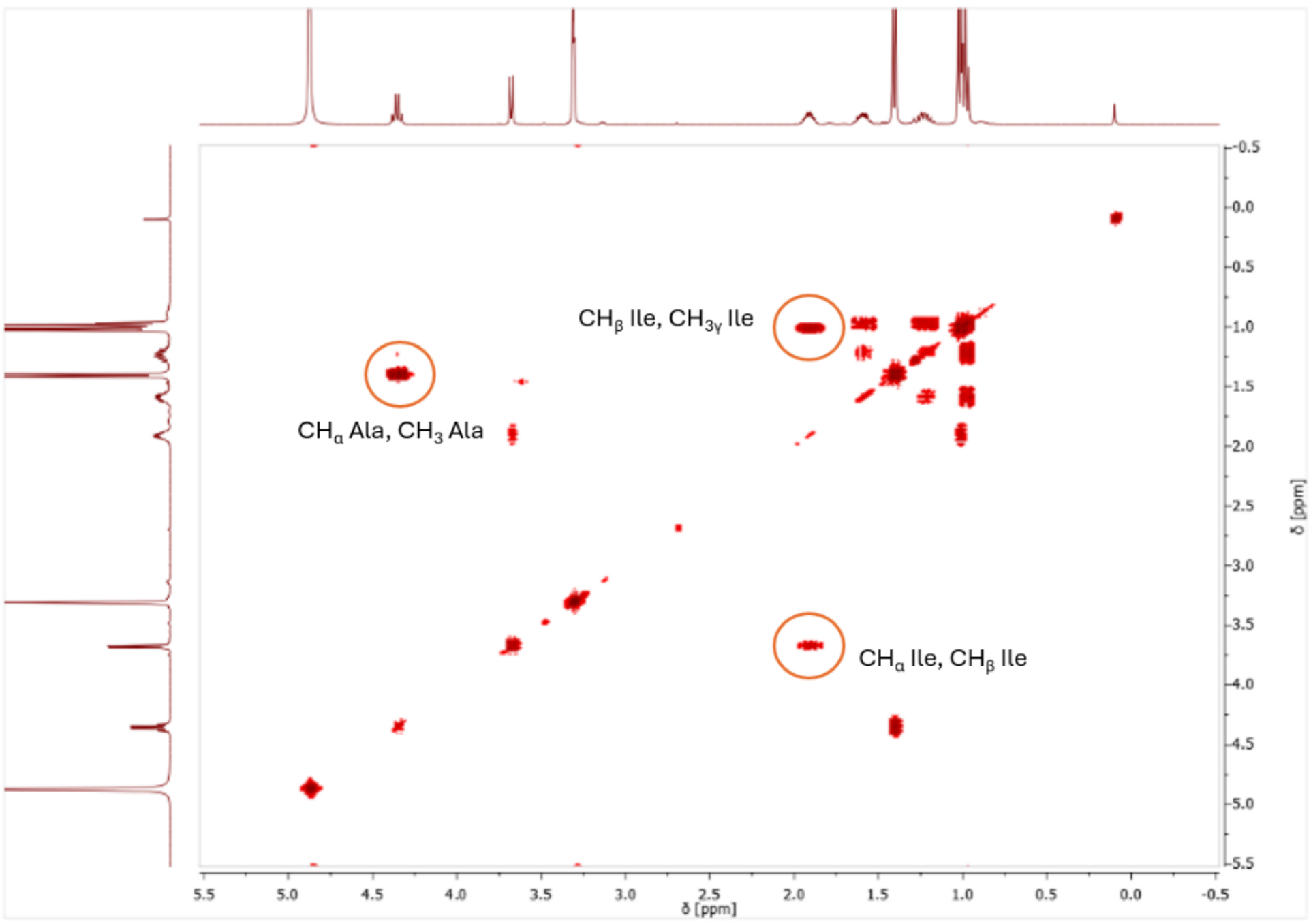

Figure S2: H-H COSY (400 MHz, MeOD-d_4) of D-Ile-L-Ala.

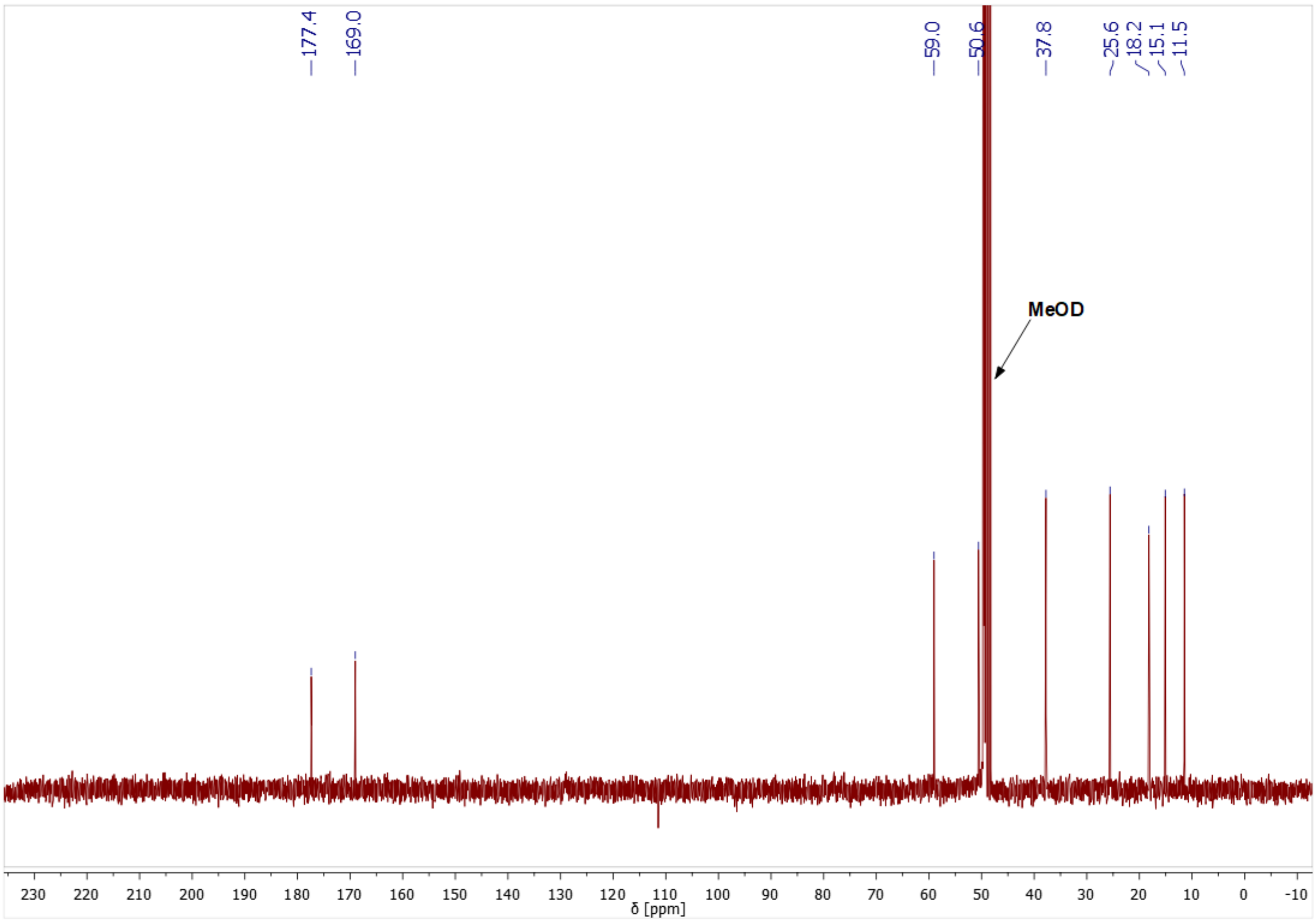

Figure S3: ^{13}C NMR (100 MHz, MeOD-*d₄*) of D-Ile-L-Ala.

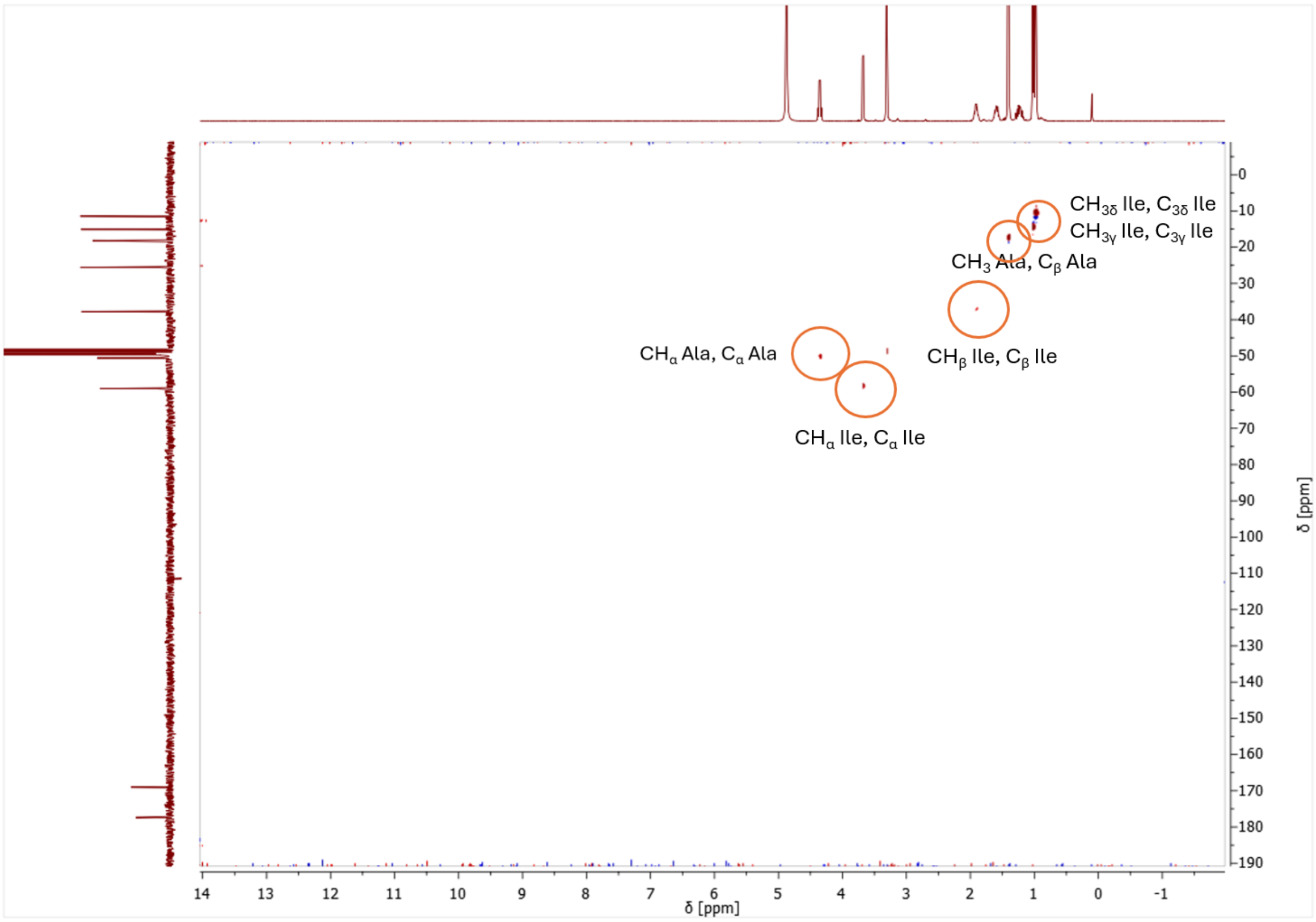

Figure S4: HSQCAD (100 MHz, MeOD-*d₄*) of D-Ile-L-Ala.

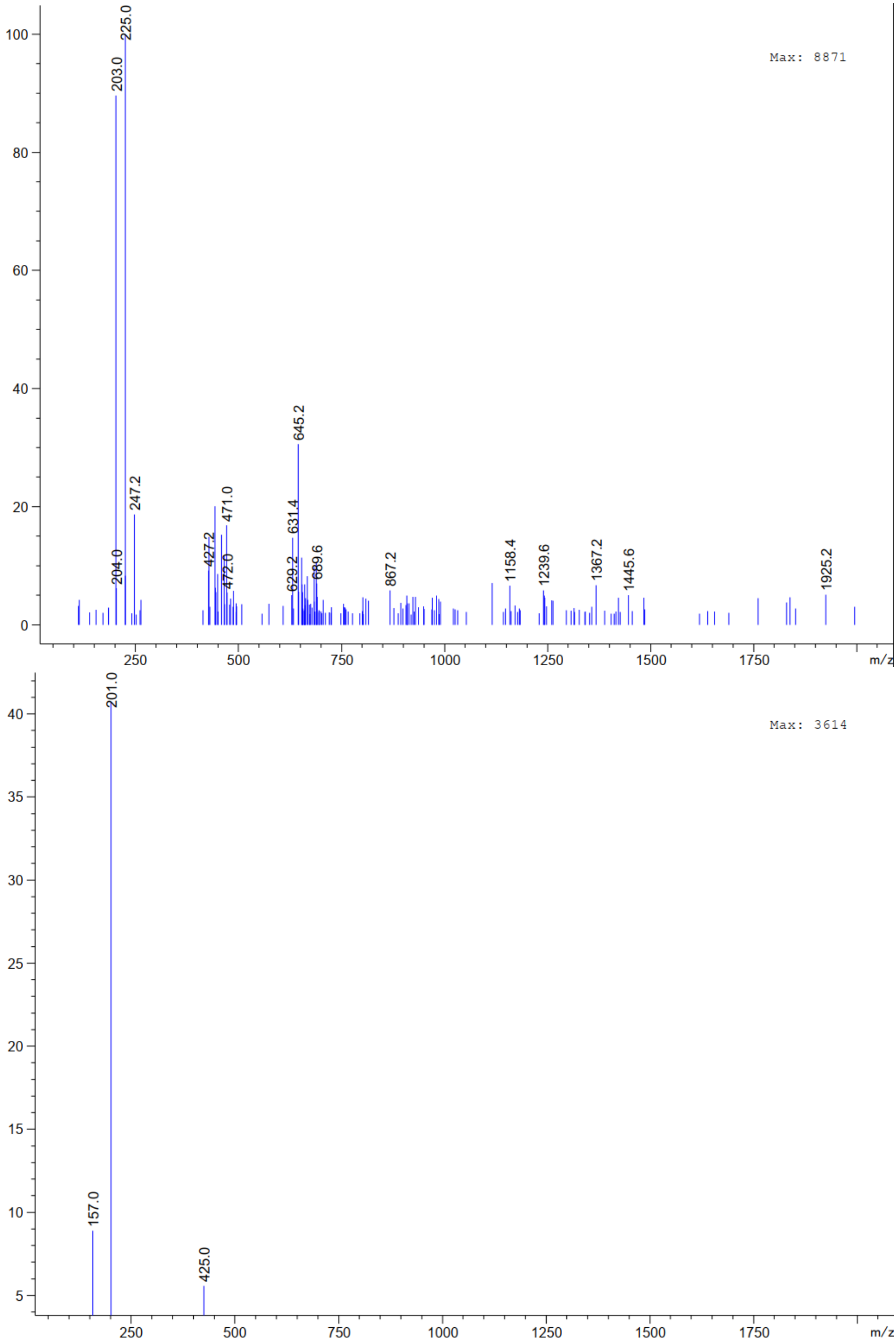

Figure S5: ESI mass spectrum of D-Ile-L-Ala. Positive (top) and negative mode (bottom).

2. D-Ile-L-Val spectroscopic data

^{1}H NMR (500 MHz, DMSO-d_6, TMS) δ (ppm): 8.60 (d, J = 8.8 Hz, 1H, NH), 4.24 (dd, J = 8.8 Hz, J = 5.4 Hz, 1H, $CH_α$ Val), 3.79 (d, J = 5.0 Hz, 1H, $CH_α$ Ile), 2.10 (m, $CH_β$, 1H, Val), 1.83 (m, $CH_β$, 1H, Ile), 1.47 (m, $CH_γ$, 1H, Ile), 1.10 (m, $CH_γ$, 1H, Ile), 0.94 (d, J = 7.0 Hz, 3H, $CH_{3γ}$ Ile), 0.89 (d, J = 7.0 Hz, 6H, $CH_{3γ}$ Val), 0.85 (dd, J = 7.0 Hz, J = 7.0 Hz, 3H, $CH_{3δ}$, Ile). ^{13}C NMR (125 MHz, DMSO-d_6, TMS) δ (ppm): 172.5 (1C, COOH), 168.4 (1C, CN), 57.4 (1C, $C_α$ Val), 56.7 (1C, $C_α$ Ile), 36.6 (1C, $C_β$ Ile), 30.0 (1C, $C_β$ Val), 23.5 (1C, $CH_{2γ}$ Ile), 19.2 (1C, $CH_3γ$ Val), 17.9 (1C, $CH_3γ$ Val), 14.8 (1C, $CH_{3γ}$ Ile), 11.3 (1C, $CH_{3δ}$ Ile)

In the H–H COSY spectrum it is possible to observe the coupling of $CH_α$ Ala with NH, $CH_α$ Ala with $CH_β$ Val, $CH_α$ Ile with $CH_β$ Ile and $CH_β$ Ile and $CH_{3γ}$ Ile, as highlighted in the spectrum (Fig. S7). Similarly, the cross-peaks are highlighted in the HSQCAD spectrum (Fig. S9).

MS (ESI): m/z, 231.1 $[M+H]^+$, 229.0 $[M-H]^-$.

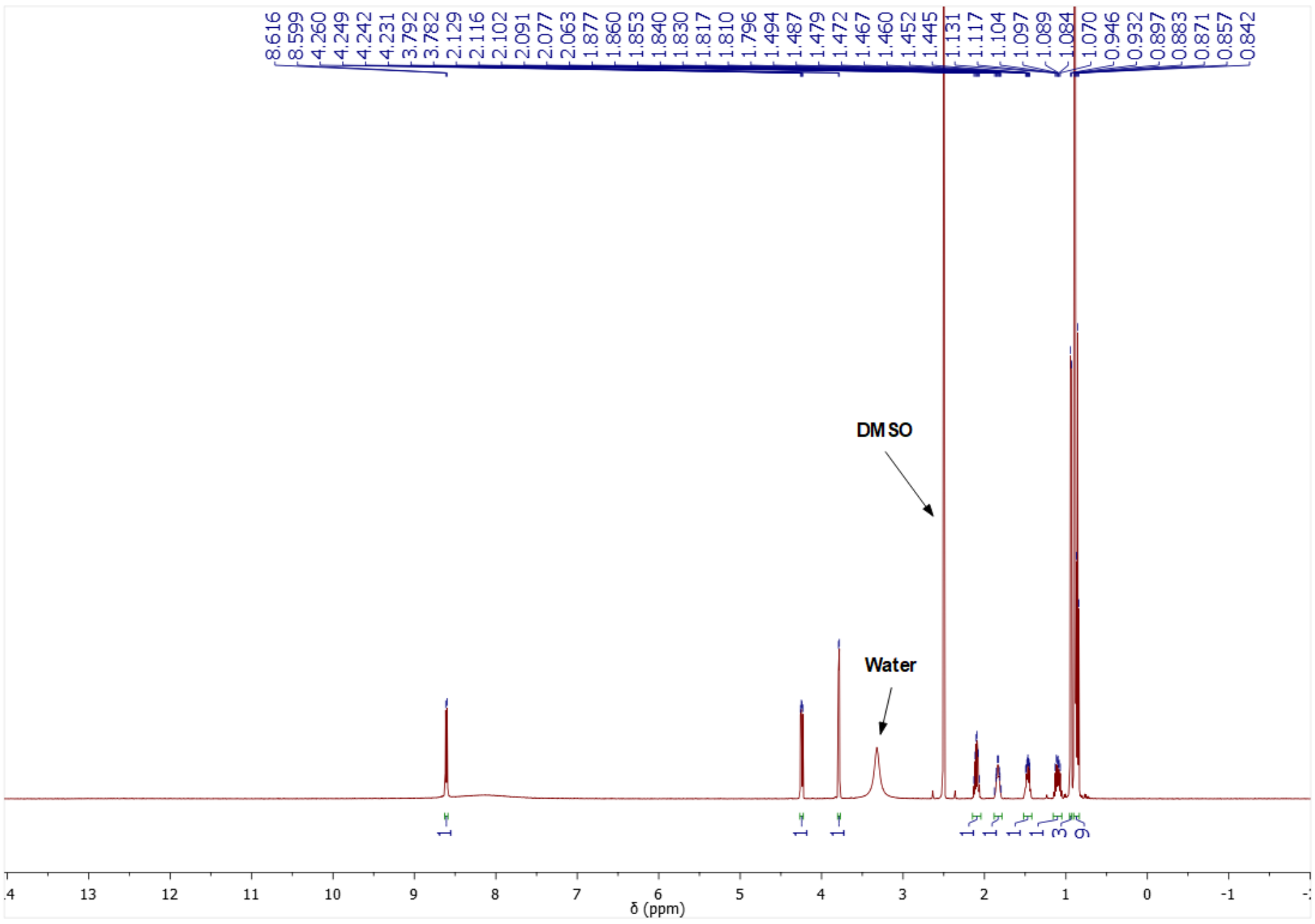

Figure S6: ^{1}H NMR (500 MHz, DMSO-d_6) of D-Ile-L-Val.

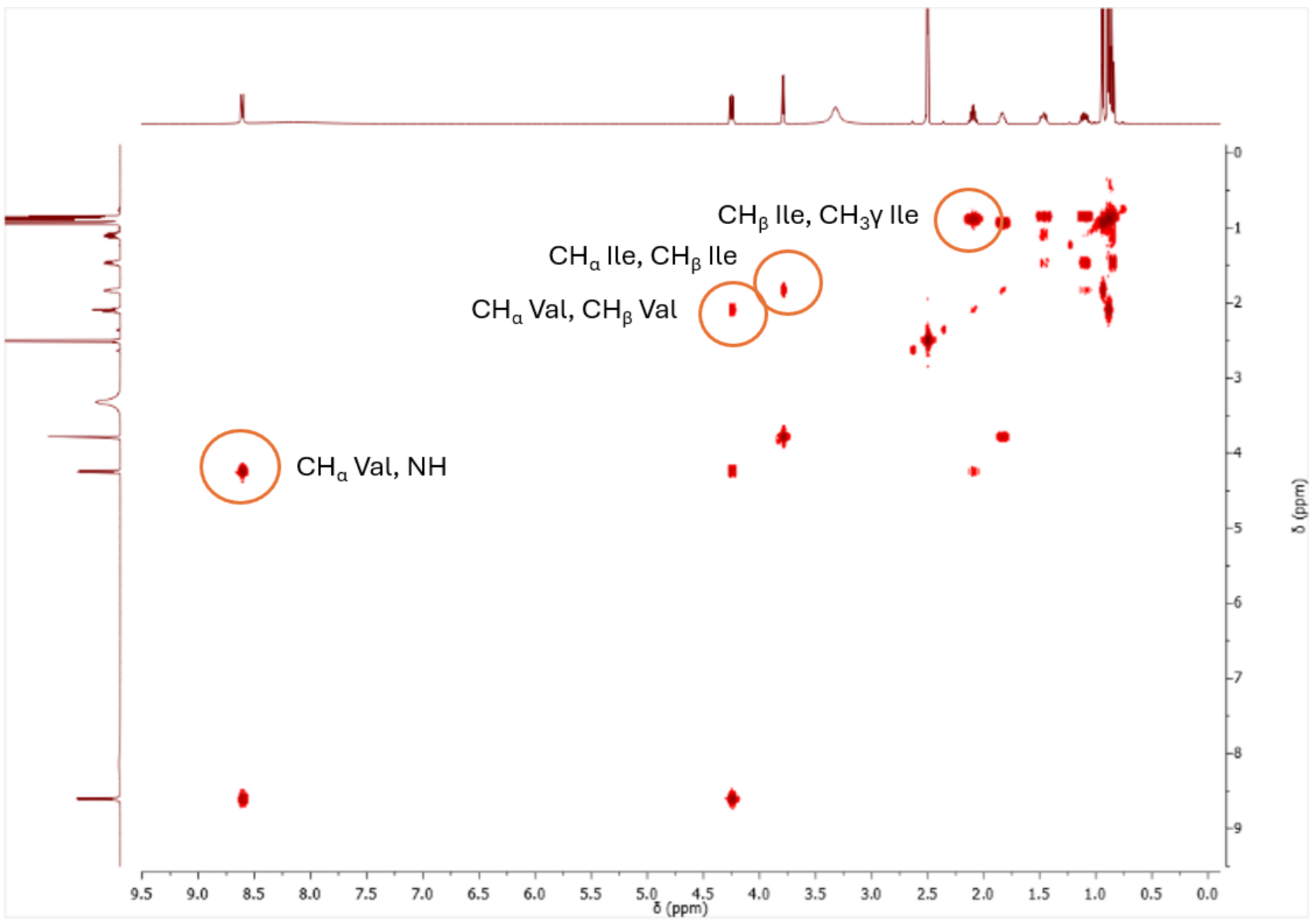

Figure S7: H-H COSY (500 MHz, DMSO-d_6) of D-Ile-L-Val.

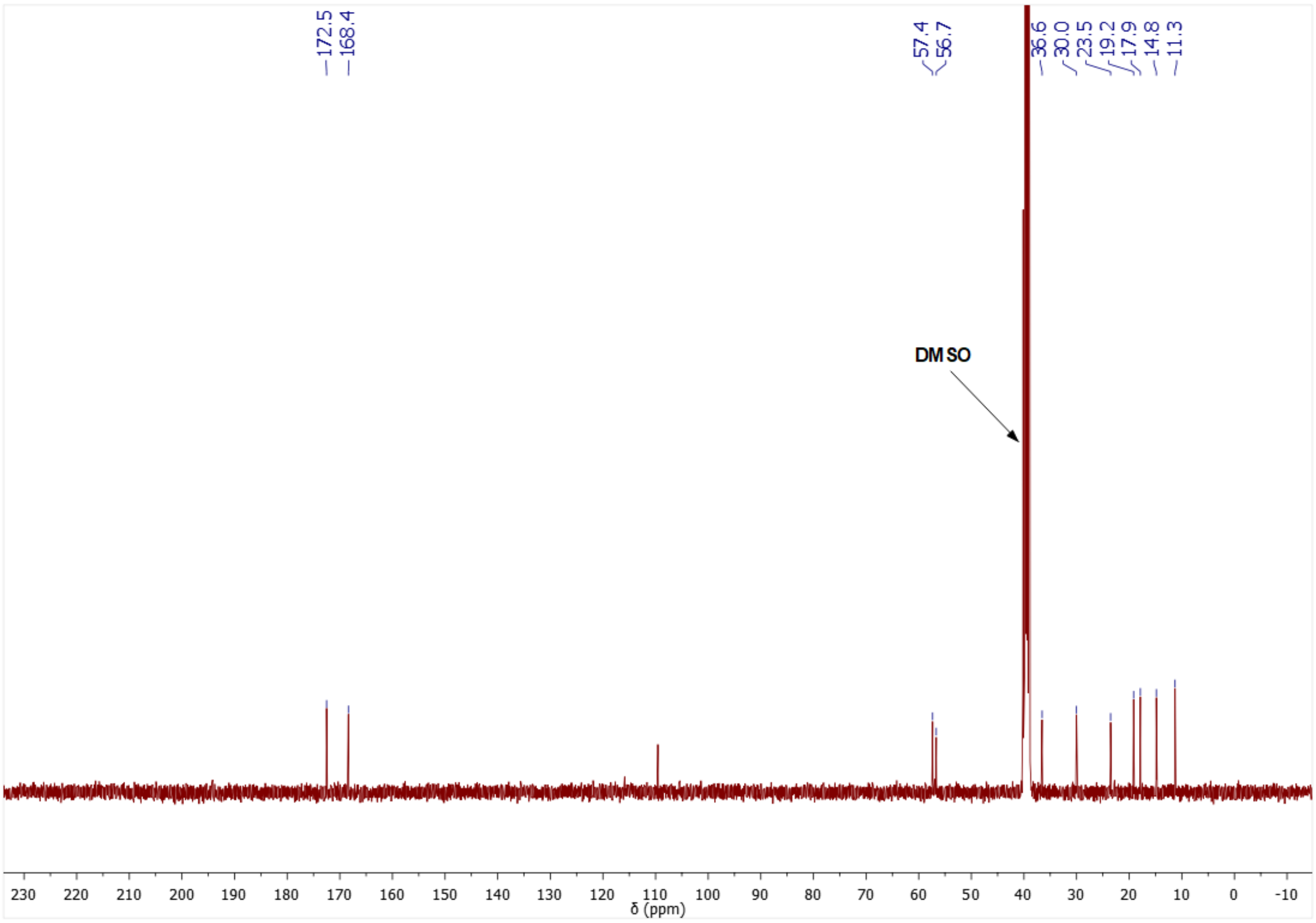

Figure S8: ^{13}C NMR (125 MHz, DMSO-d_6) of D-Ile-L-Val.

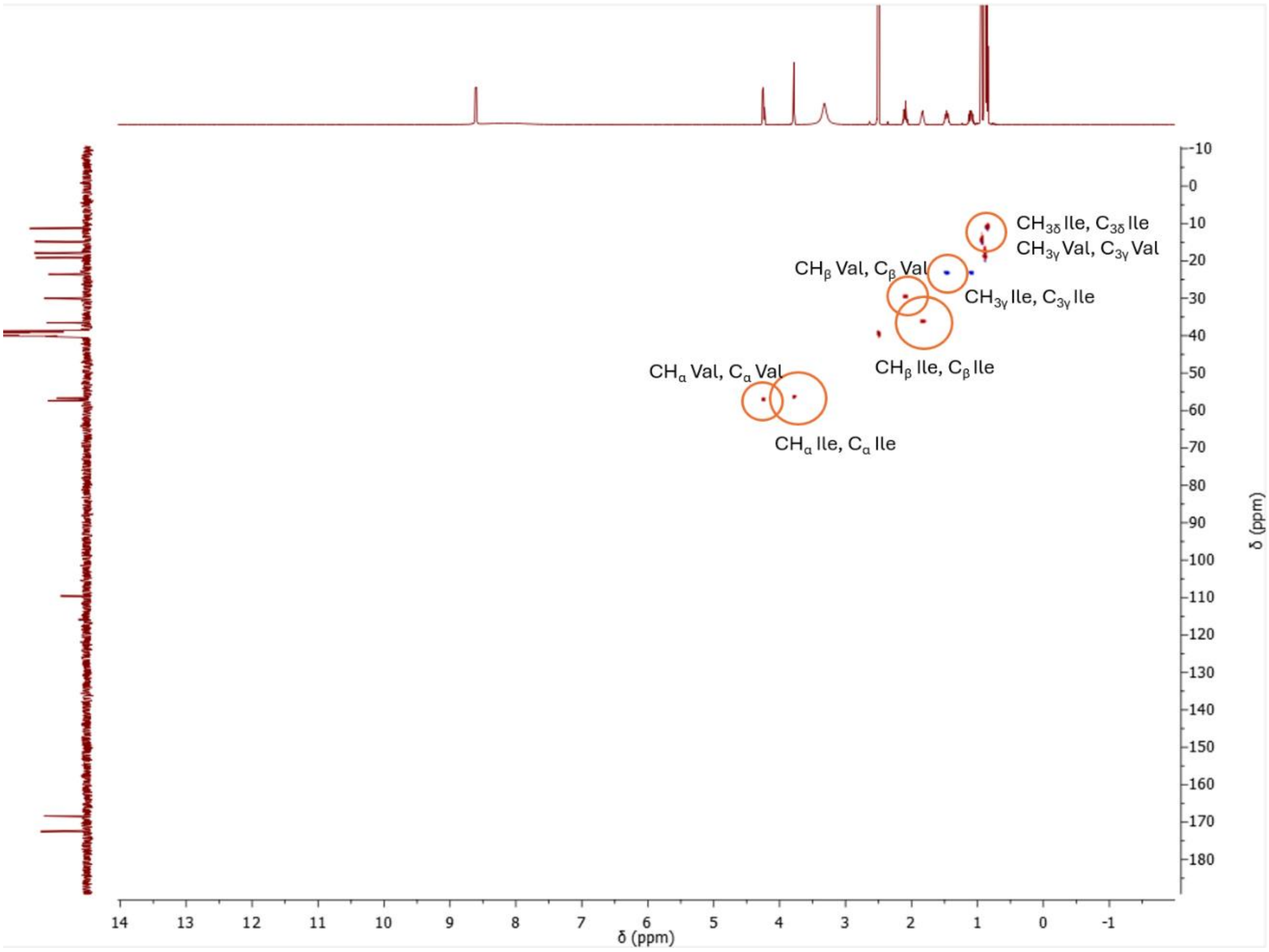

Figure S9: HSQCAD (125 MHz, DMSO-d_6) of D-Ile-L-Val.

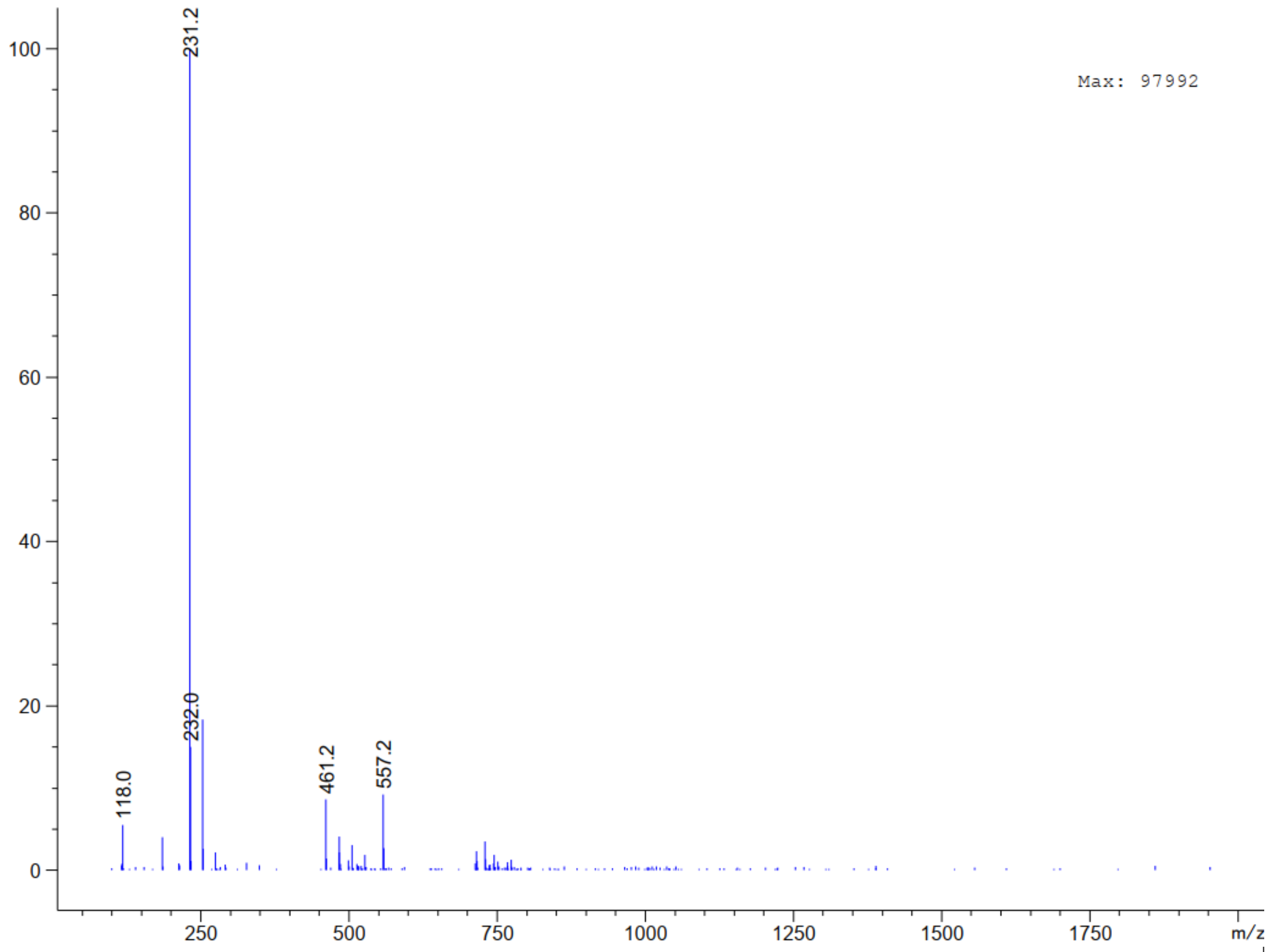

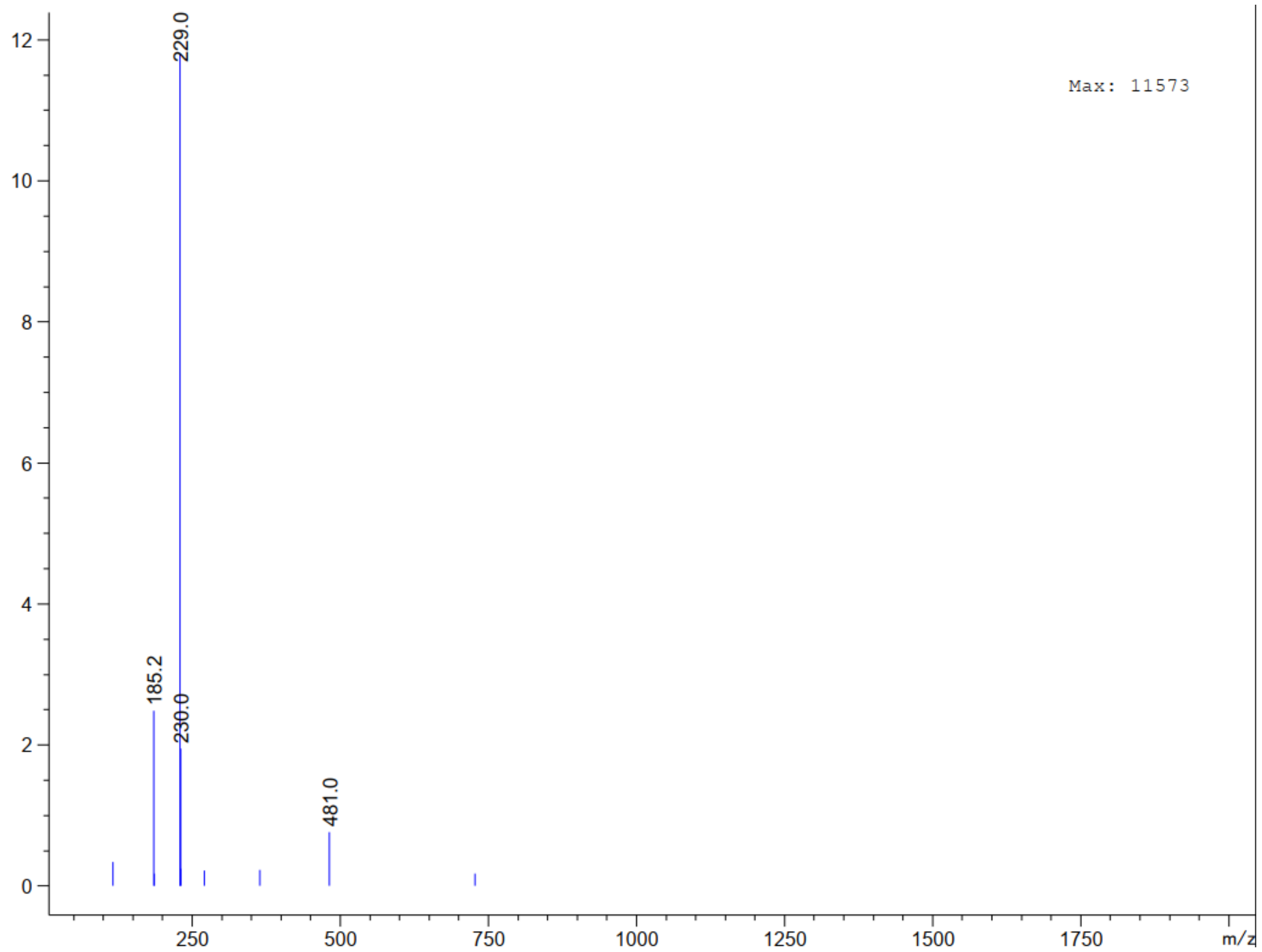

Figure S10: ESI mass spectrum of D-Ile-L-Val. Positive (top) and negative mode (bottom).

3. D-Ile-L-Leu spectroscopic data

^{1}H NMR (500 MHz, DMSO-d_6, TMS) δ (ppm): 8.61 (d, J = 8.0 Hz, 1, NH), 4.27 (ddd, J = 5.0 Hz, J = 8.0 Hz, J = 10.0 Hz, 1, CH_α Leu), 3.62 (d, J = 5.0 Hz, 1, CH_α Ile), 1.80 (m, 1, CH_β Ile) 1.65-1.48 (m, 3, CH_β Leu, CH_γ Leu), 1.42 (m, 1, CH_γ Ile), 1.08 (m, 1, CH_γ Ile), 0.91 (d, J = 7.0 Hz, $CH_{3\gamma}$ Ile), 0.90 (d, J = 7.0 Hz, $CH_{3\delta}$ Leu), 0.86-0.82 (m, $CH_{3\delta}$ Leu, $CH_{3\delta}$ Ile). ^{13}C NMR (125 MHz, DMSO-d_6, TMS) δ (ppm): 173.6 (1, COOH), 168.6 (1, NH), 57.0 (1, C_α Ile), 50.5 (1, C_α Leu), 39.3 (1, C_β Leu), 36.7 (1, C_β Ile), 24.4 (1, C_γ Leu), 23.6 (1, C_γ Ile), 22.9 (1, C_δ Leu), 20.9 (1, C_δ Leu), 14.9 (1, C_γ Ile), 11.3 (1, C_δ Ile).
In the H–H COSY spectrum it is possible to observe the coupling of CH_α Leu with NH, CH_α Leu with CH_β Leu and with CHγ Leu, CH_α Ile with CH_β Ile and CH_β Ile and $CH_{3\gamma}$ Ile, as highlighted in the spectrum (Fig. S12). Similarly, the cross-peaks are highlighted in the HSQCAD spectrum (Fig. S14).
MS (ESI): m/z, 245.2 [M+H]$^+$, 243.0 [M-H]$^-$.

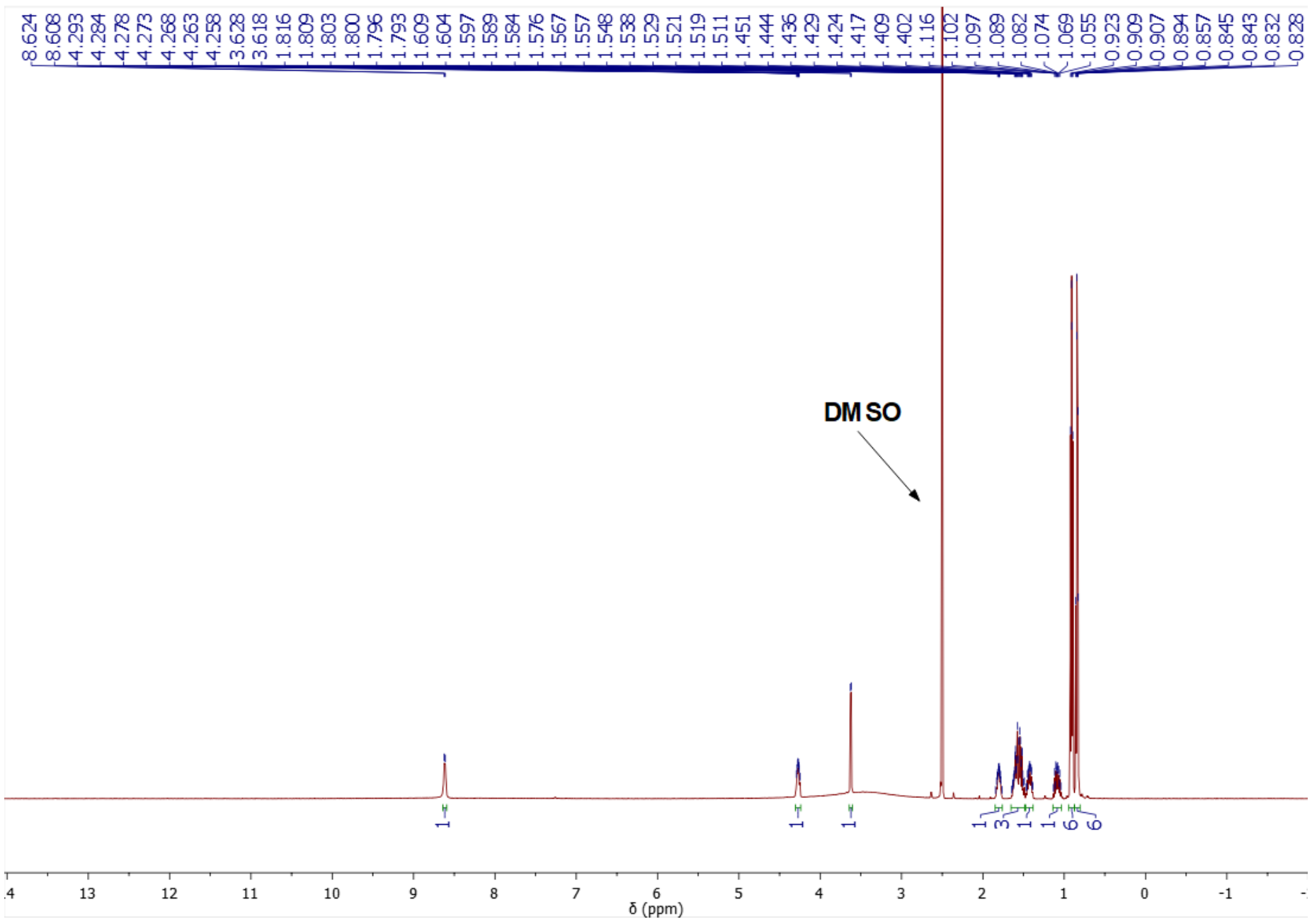

Figure S11: ^{1}H NMR (500 MHz, DMSO-d_6) of D-Ile-L-Leu.

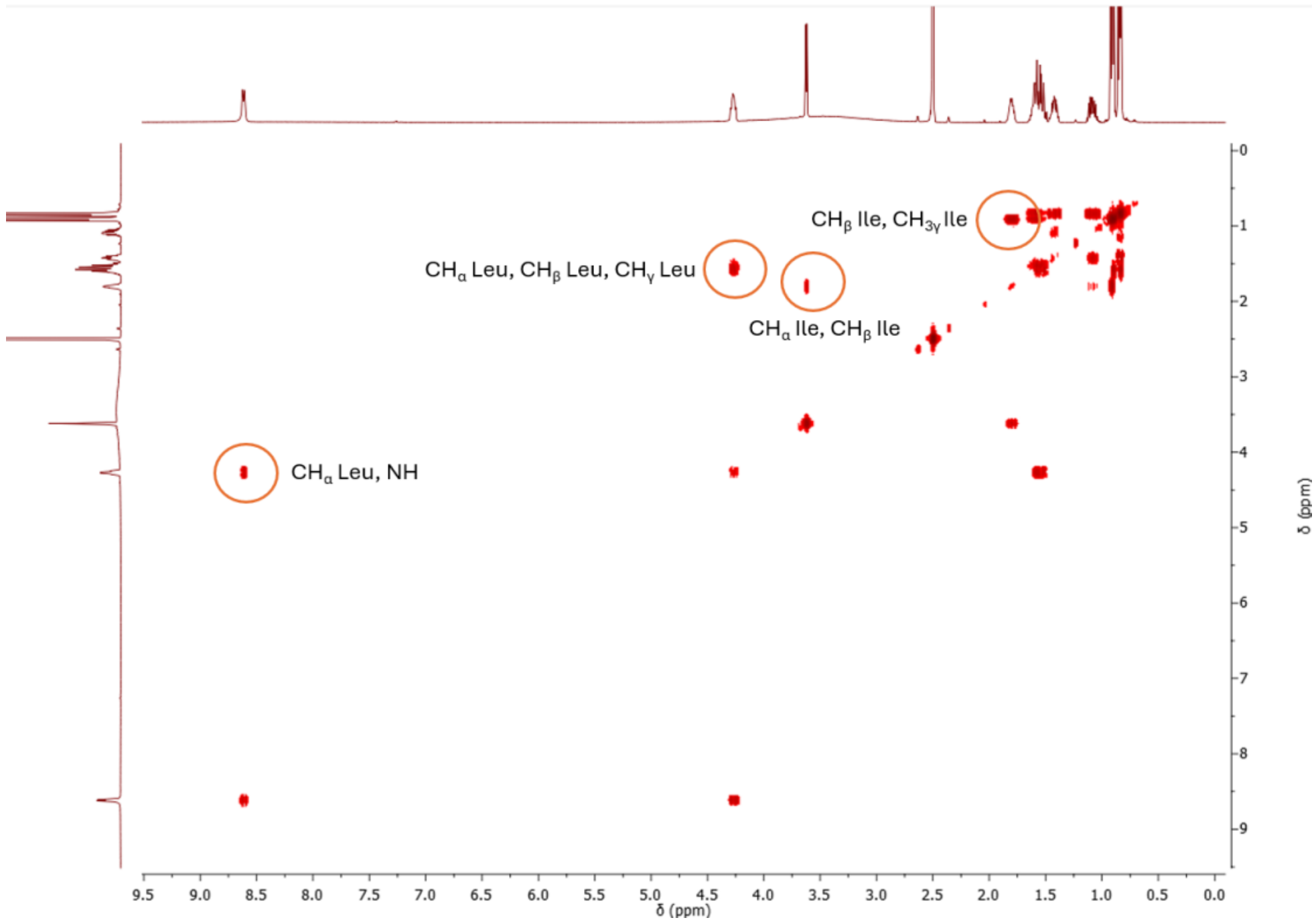

Figure S12: H-H COSY (500 MHz, DMSO-d_6) of D-Ile-L-Leu.

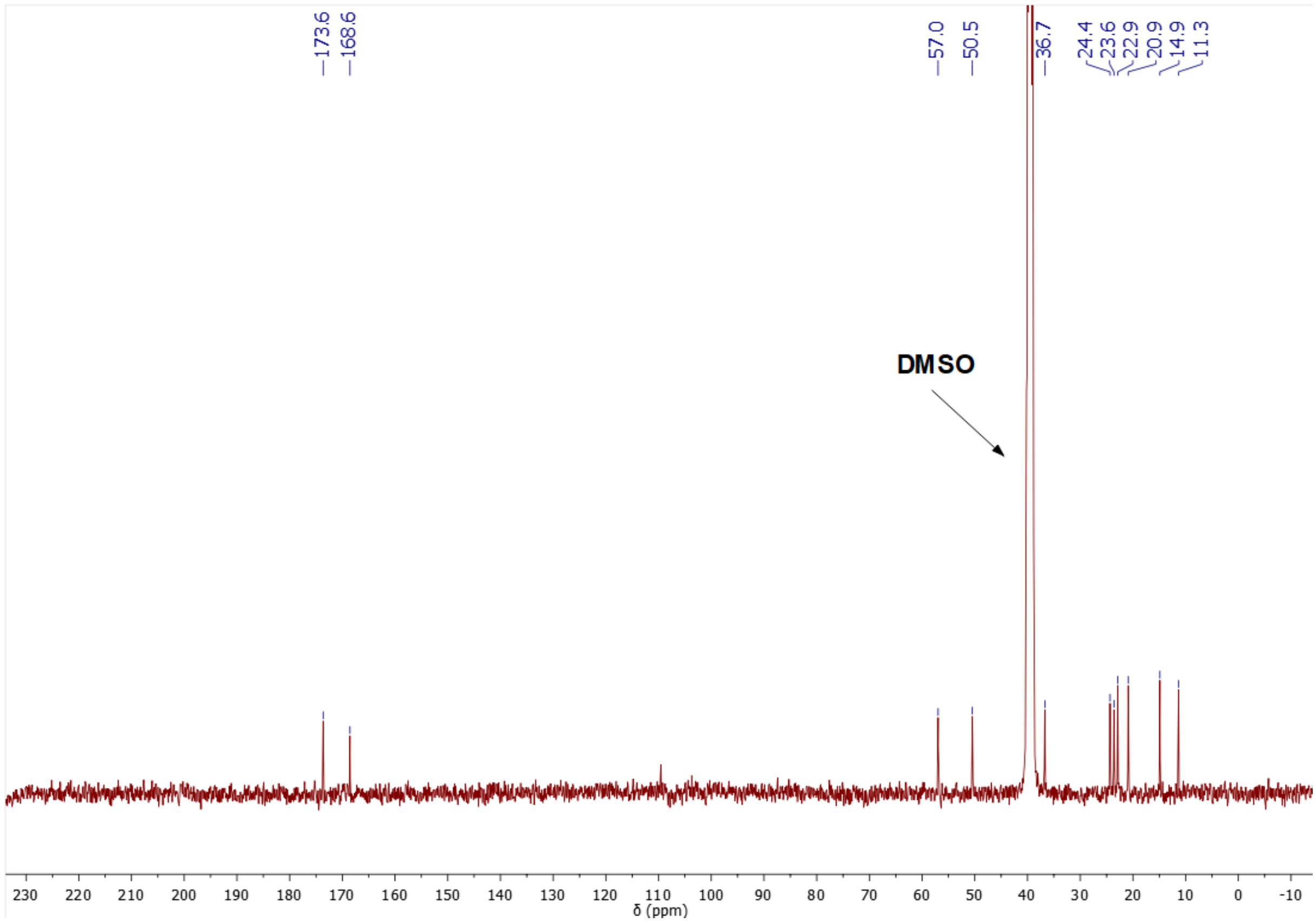

Figure S13: ^{13}C NMR (125 MHz, DMSO-d_6) of D-Ile-L-Leu.

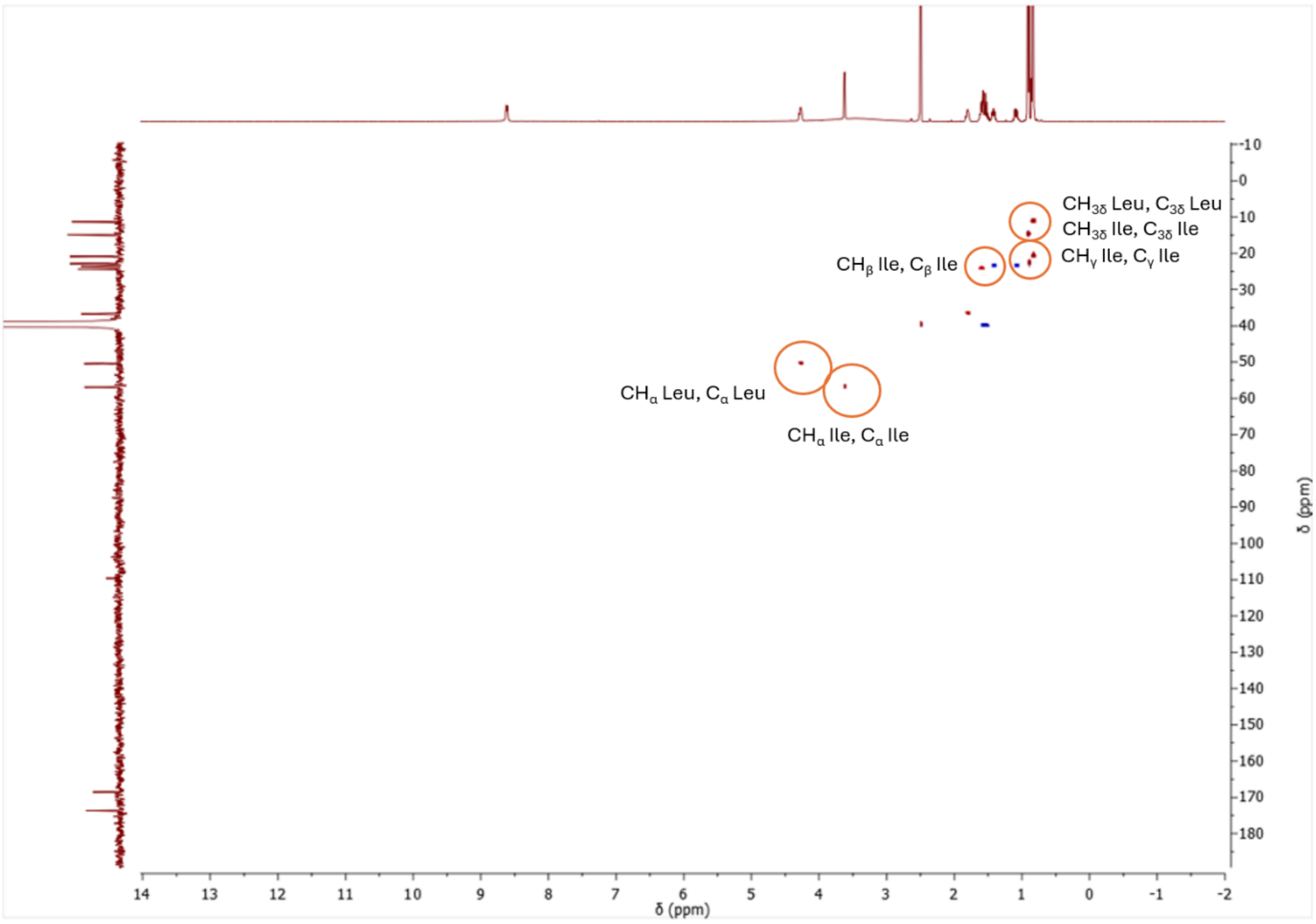

Figure S14: HSQCAD (125 MHz, DMSO-d_6) of D-Ile-L-Leu.

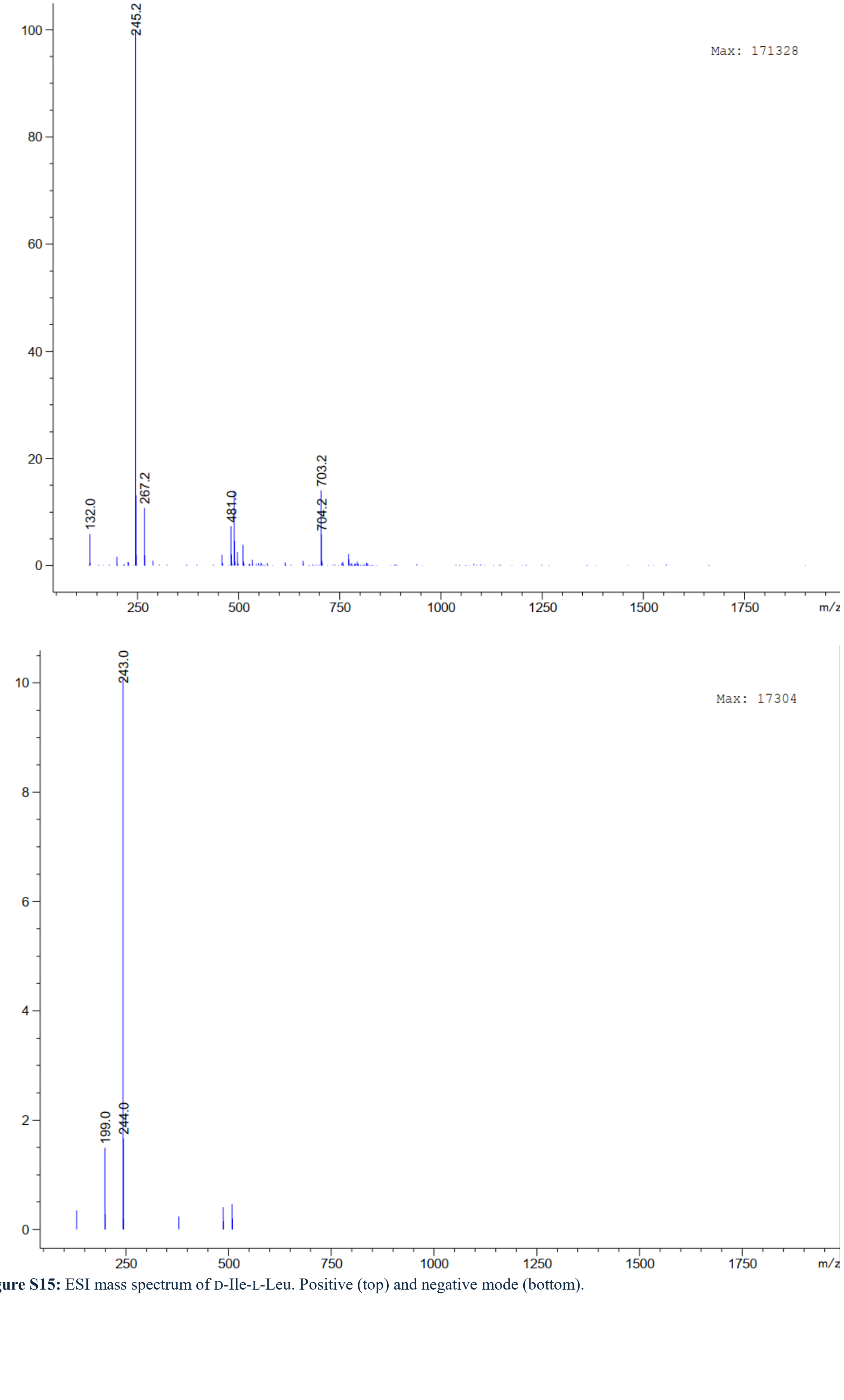

Figure S15: ESI mass spectrum of D-Ile-L-Leu. Positive (top) and negative mode (bottom).

4. D-Ile-L-Ile spectroscopic data

^{1}H NMR (500 MHz, DMSO-d_6, TMS) δ (ppm): 8.61 (d, J = 8.5 Hz, 1, NH), 4.28 (dd, J = 6.0 Hz, J = 8.5 Hz, 1, $CH_α$ IleC), 3.78 (d, J = 5.5 Hz, 1, $CH_α$ IleN), 1.82 (m, 2, $CH_β$ IleN, IleC), 1.43 (m, 2, $CH_{2δ}$, IleN, IleC), 1.19 (m, 1, $CH_{2γ}$ Ile), 1.01 (m, $CH_{2γ}$ Ile), 0.93 (d, J = 7.0 Hz, 3, $CH_{3γ}$ Ile), 0.88-0.83 (m, 6, $CH_{3γ}$, $CH_{3δ}$ Ile). ^{13}C NMR (125 MHz, DMSO-d_6, TMS) δ (ppm): 172.5 (1, COOH), 168.3 (1, CN), 56.6 ($C_α$ IleN), 56.4 ($C_α$ IleC), 36.6, 36.5 (2, $C_β$), 24.6 (1, $CH_{2γ}$), 23.5 (1, $CH_{2γ}$), 15.6 (1, $CH_{3γ}$), 14.8 (1, $CH_{3γ}$), 11.3, 11.0 (2, $CH_{3δ}$).
In the H–H COSY spectrum it is possible to observe the coupling of $CH_α$ C-terminal Ile (IleC) with NH, $CH_α$ C-terminal Ile (IleC) with $CH_β$, $CH_α$ N-terminal Ile (IleN) with $CH_β$ (Fig. S17). Similarly, the cross-peaks are highlighted in the HSQCAD spectrum (Fig. S19).
MS (ESI): m/z, 245.2 [M+H]$^+$, 243.0 [M-H]$^-$.

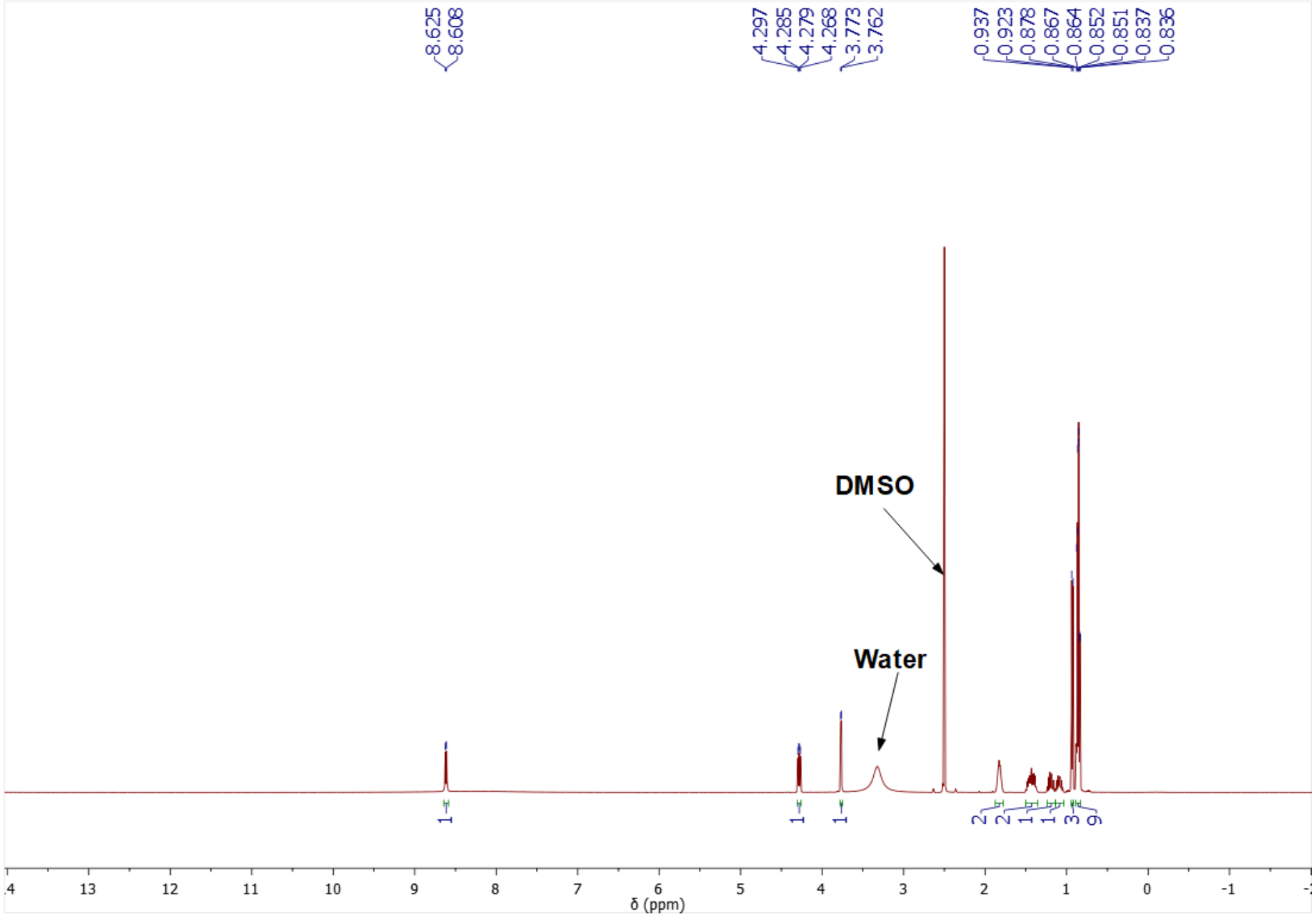

Figure S16: ^{1}H NMR (500 MHz, DMSO-d_6) of D-Ile-L-Ile.

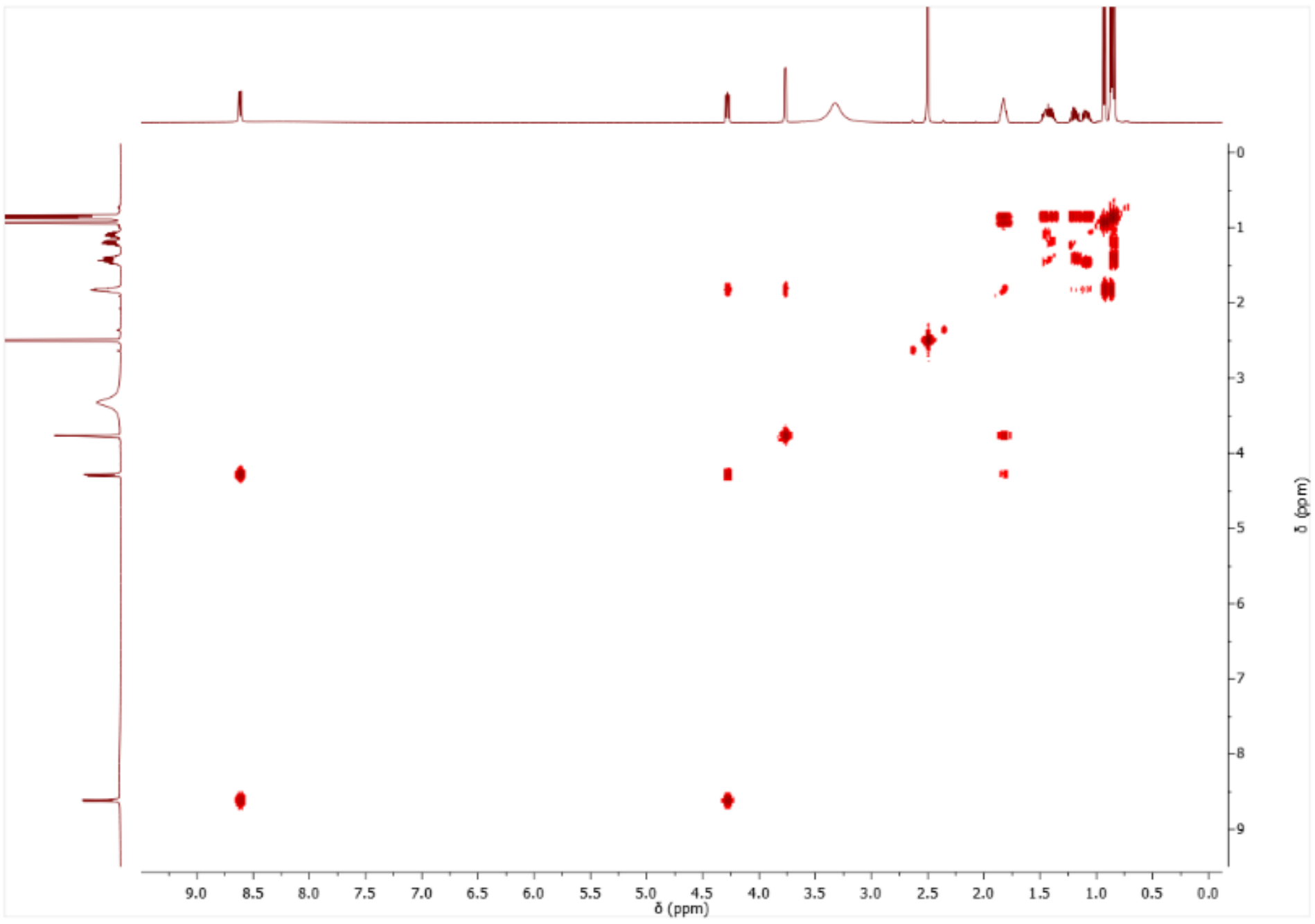

Figure S17: H-H COSY (500 MHz, DMSO-d_6) of D-Ile-L-Ile.

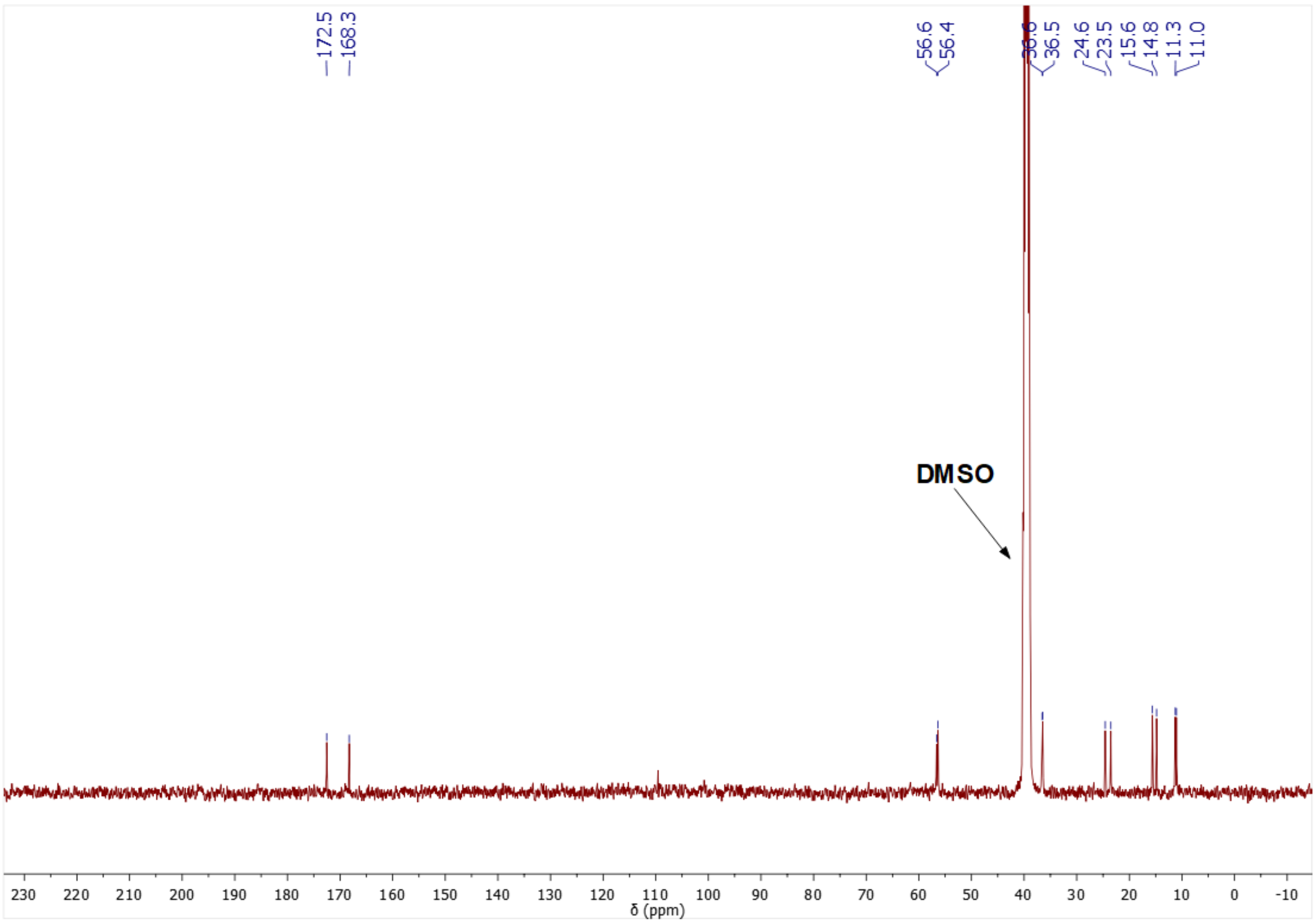

Figure S18: ^{13}C NMR (125 MHz, DMSO-d_6) of D-Ile-L-Ile.

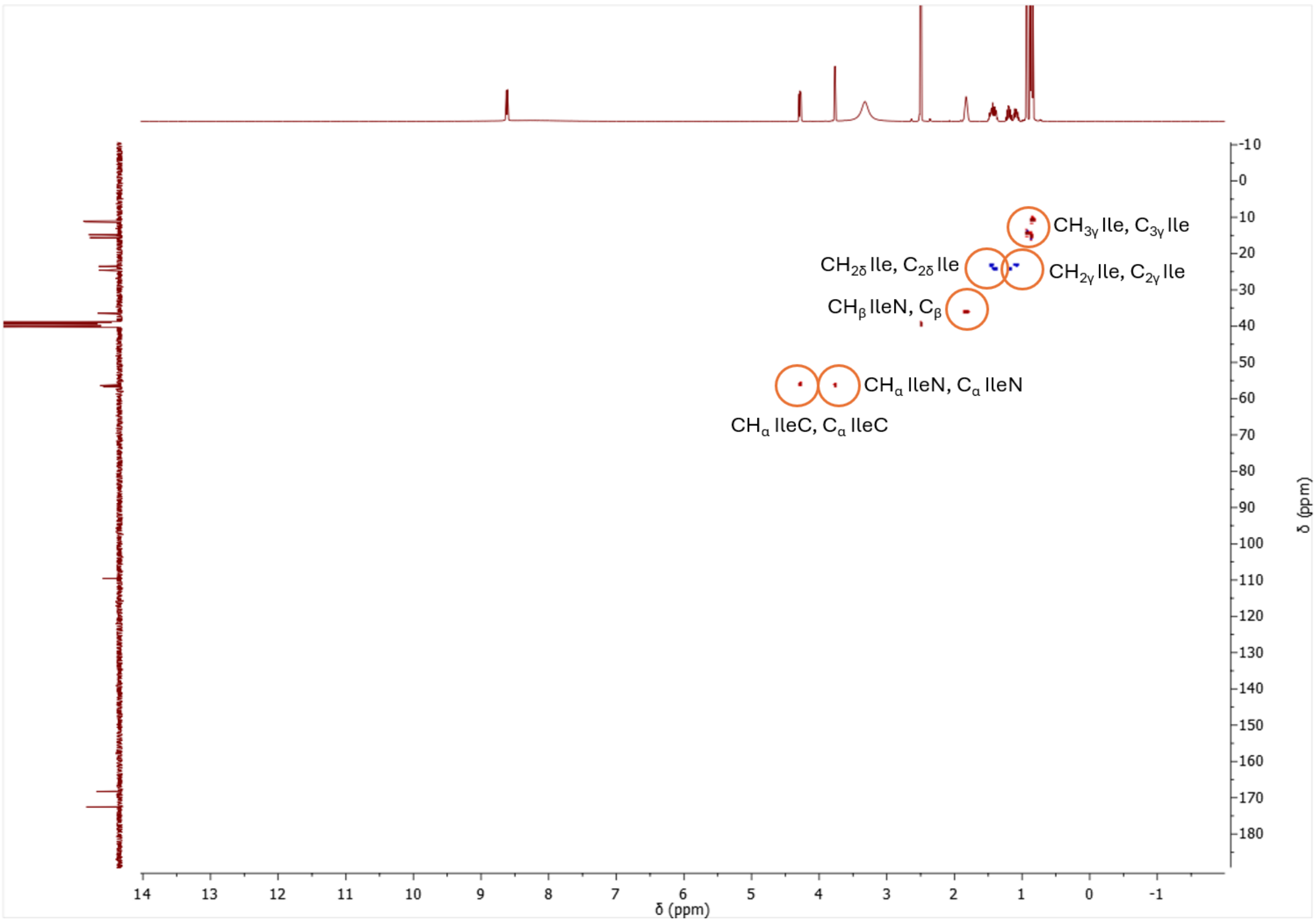

Figure S19: HSQCAD (125 MHz, DMSO-d_6) of D-Ile-L-Ile.

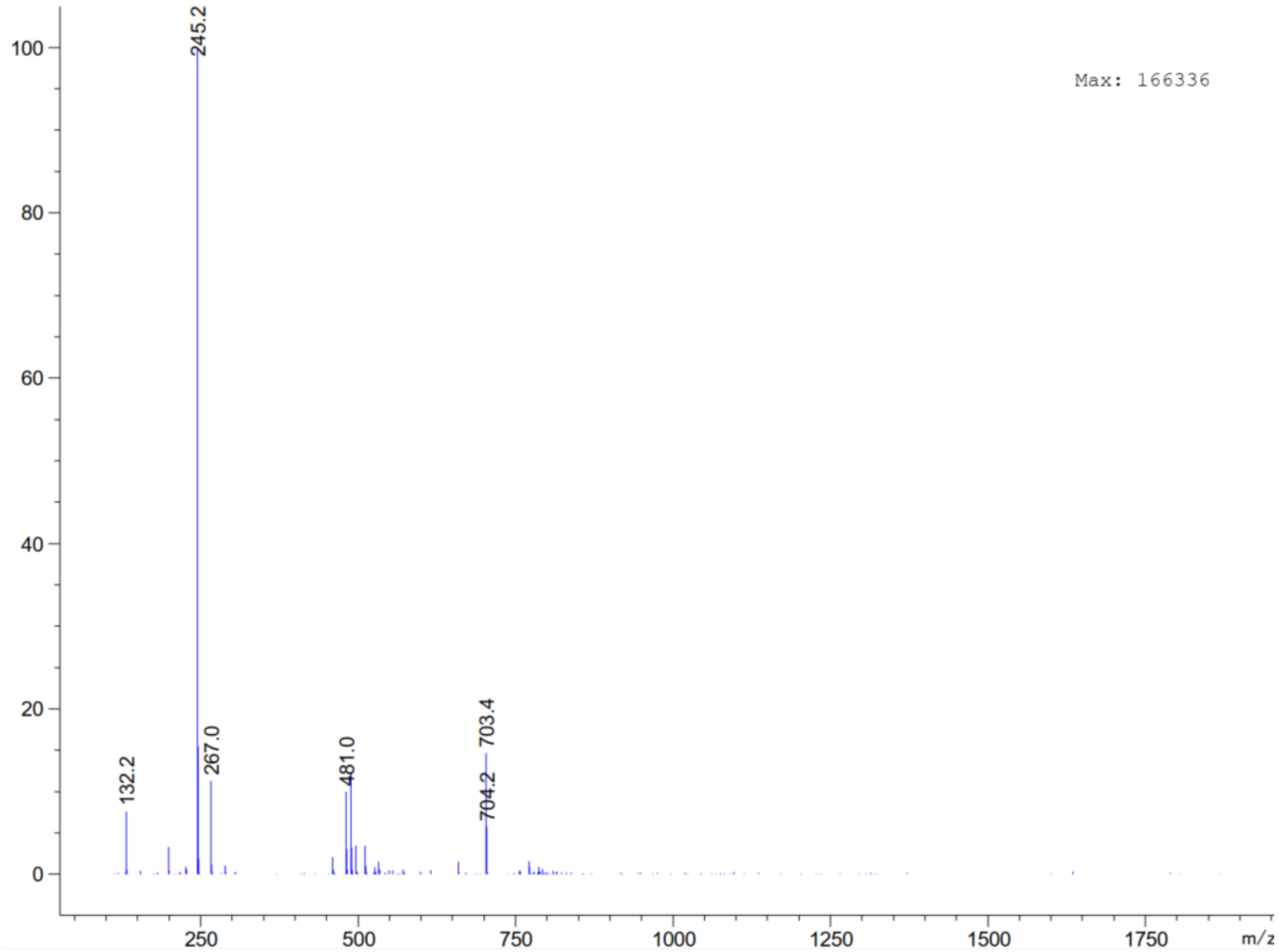

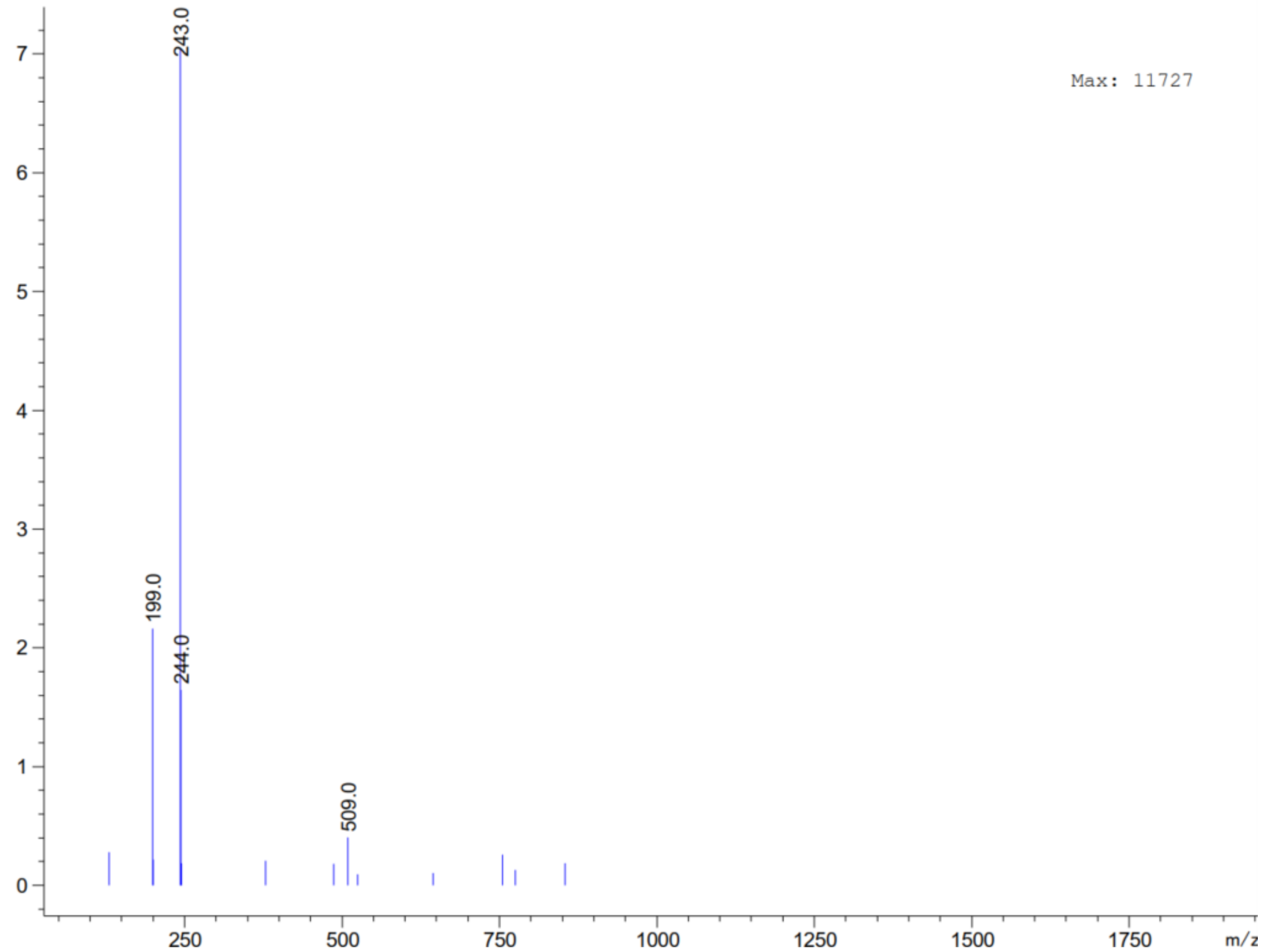

Figure S20: ESI mass spectrum of D-Ile-L-Ile. Positive (top) and negative mode (bottom).

5. L-Ile-L-Ala spectroscopic data

^{1}H NMR (400 MHz, DMSO-d_6,) δ (ppm): 8.67 (d, J = 8.0 Hz, 1H, NH), 8.27 (s (br), 3H, NH_3^+), 4.28 (qd, 1H, CH_α Ala), 3.61 (d, J = 5.7 Hz, 1H, CH_α Ile), 1.81 (m, 1H, CH_β Ile), 1.52 (m, 1H, $CH_{2\gamma}$), 1.31 (d, J = 7.3 Hz, 3H, CH_3 Ala), 1.15 (m, 1H, $CH_{2\gamma}$ Ile), 0.93 (d, J = 6.9 Hz, 3H, $CH_{3\gamma}$ Ile), 0.86 (dd, J = 7.3, 7.3 Hz, 3H, $CH_{3\delta}$ Ile). MS (ESI): m/z 203.2 $[M+H]^+$, 225.2 $[M+Na]^+$. 201.0 $[M-H]^-$.

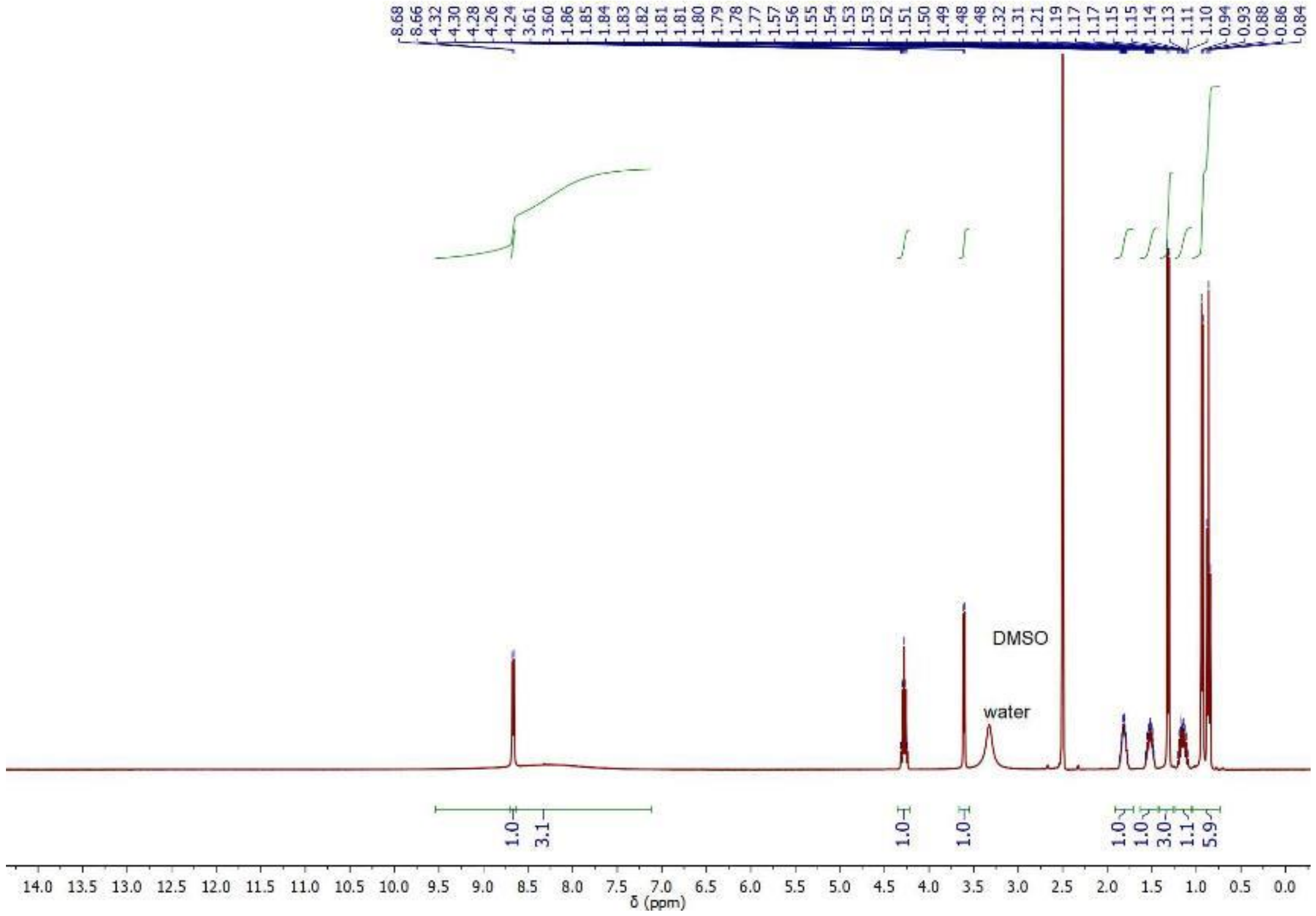

Figure S21: ^{1}H NMR (500 MHz, DMSO-d_6) of L-Ile-L-Ala.

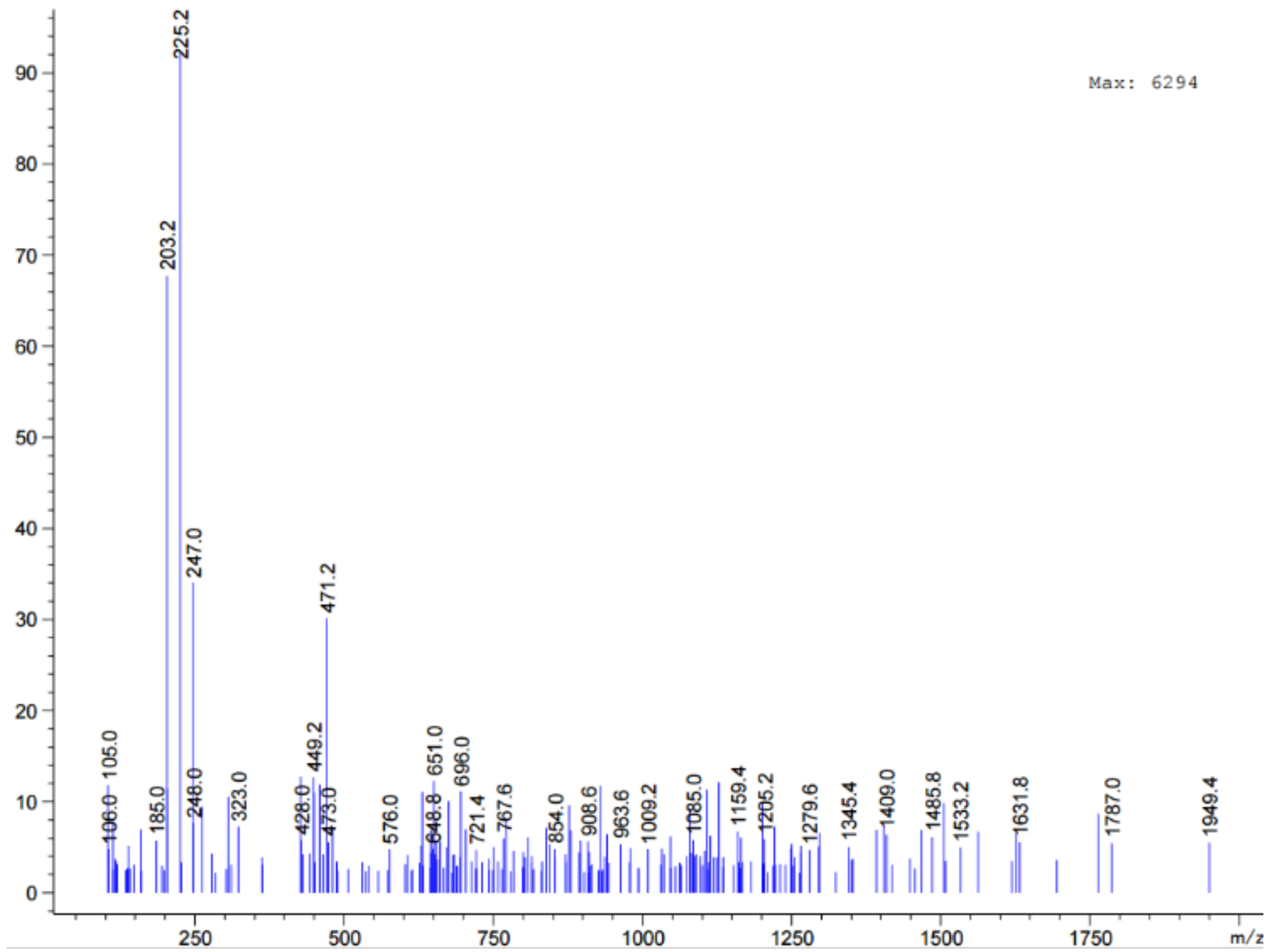

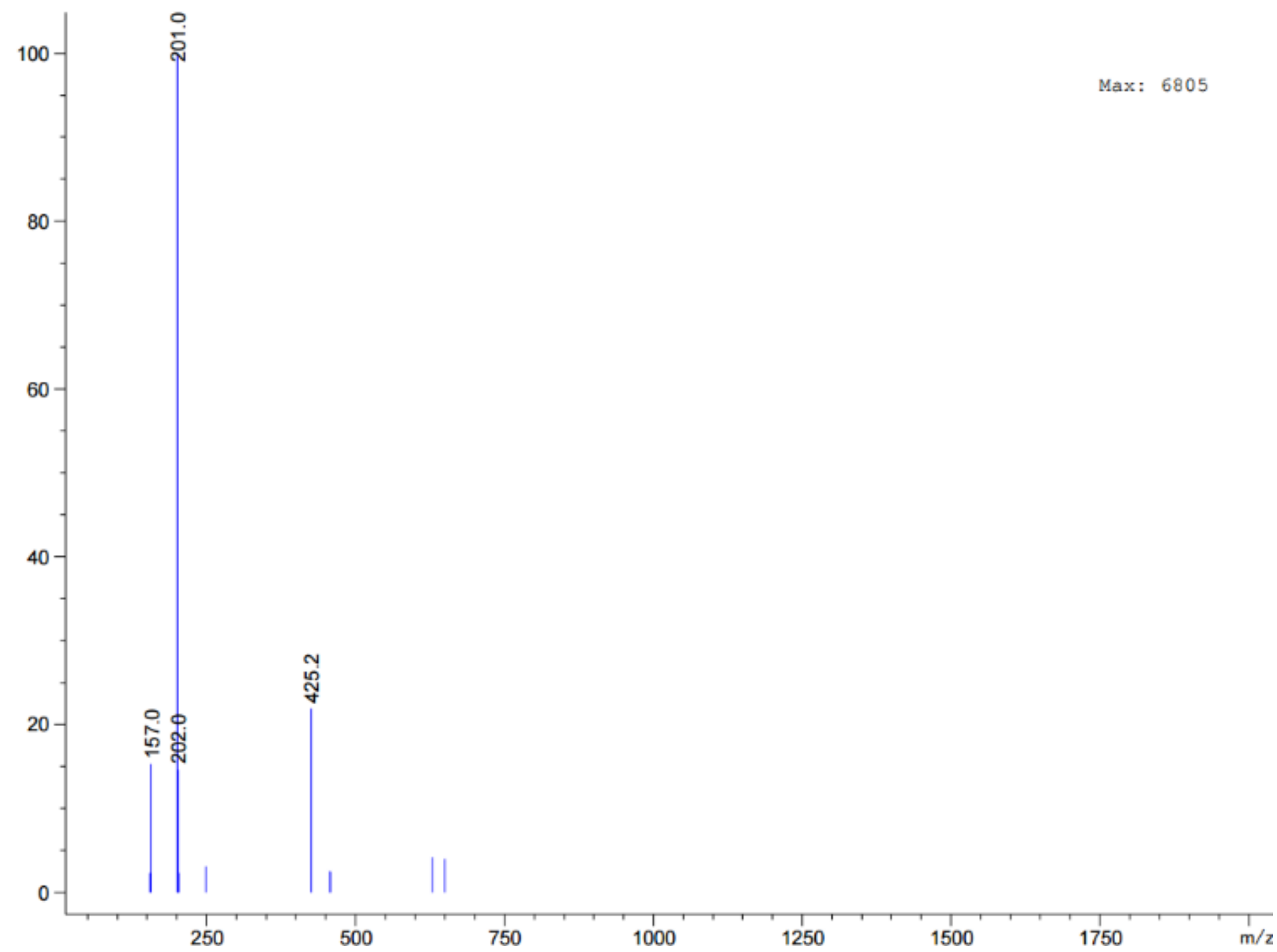

Figure S22: ESI mass spectrum of L-Ile-L-Ala. Positive (top) and negative mode (bottom).

6. L-Ile-L-Val spectroscopic data

^{1}H NMR (400 MHz, MeOD-d_4,) δ (ppm): 4.34 (d, J = 5.2 Hz, 1H, CH_α Val), 3.86 (d, J = 5.2 Hz, 1H, CH_α Ile), 2.20 (m, 1H, CH_β Val), 1.97 (m, 1H, CH_β Ile), 1.61 (m, 1H, $CH_{2\gamma}$ Ile), 1.24 (m, 1H, $CH_{2\gamma}$ Ile), 1.07-0.94 (m, 12H, 4xCH_3). MS (ESI): m/z 231.2 $[M+H]^+$, 253.0 $[M+Na]^+$. 229.0 $[M-H]^-$.

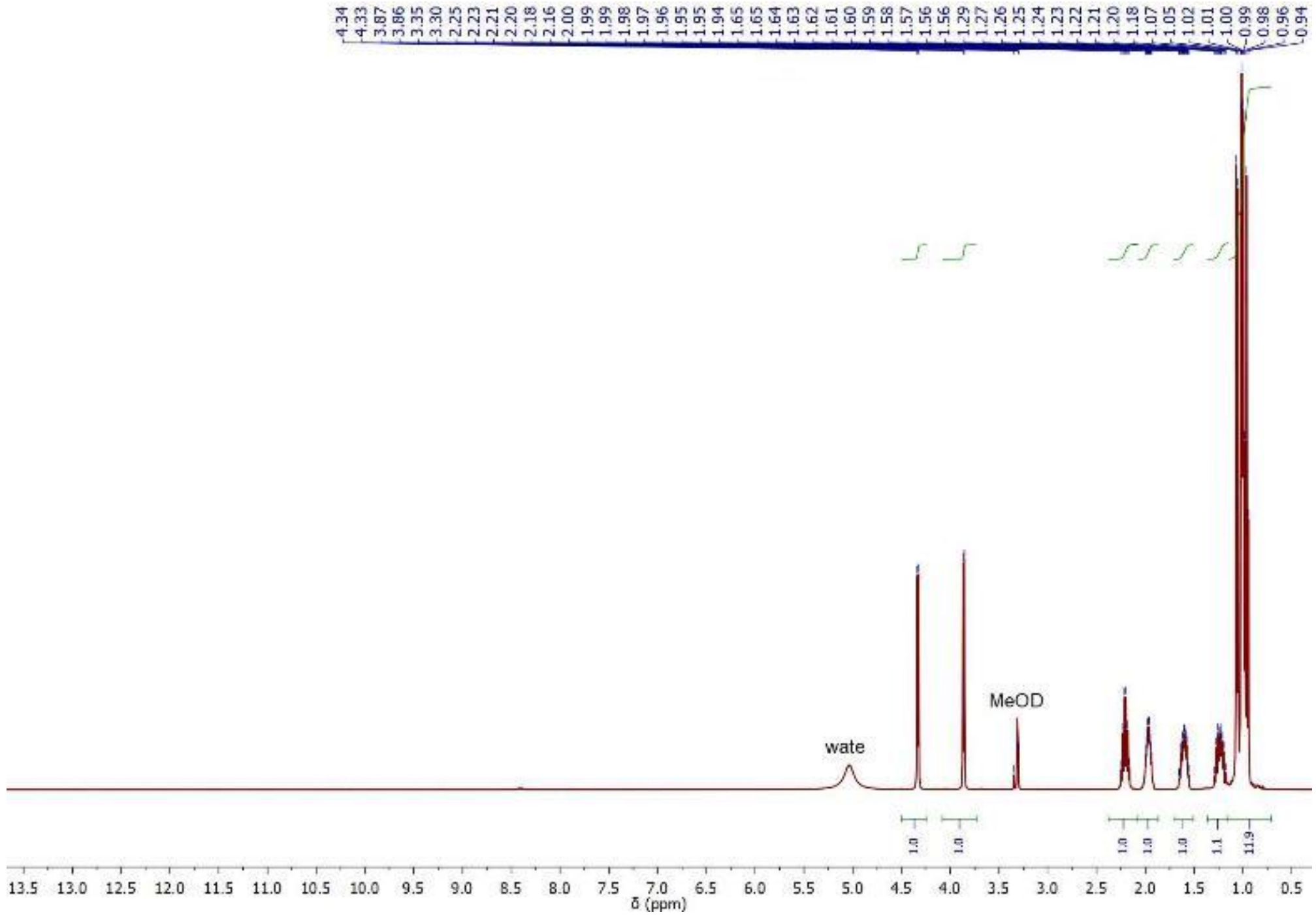

Figure S23: ^{1}H NMR (500 MHz, DMSO-d_6) of L-Ile-L-Val.

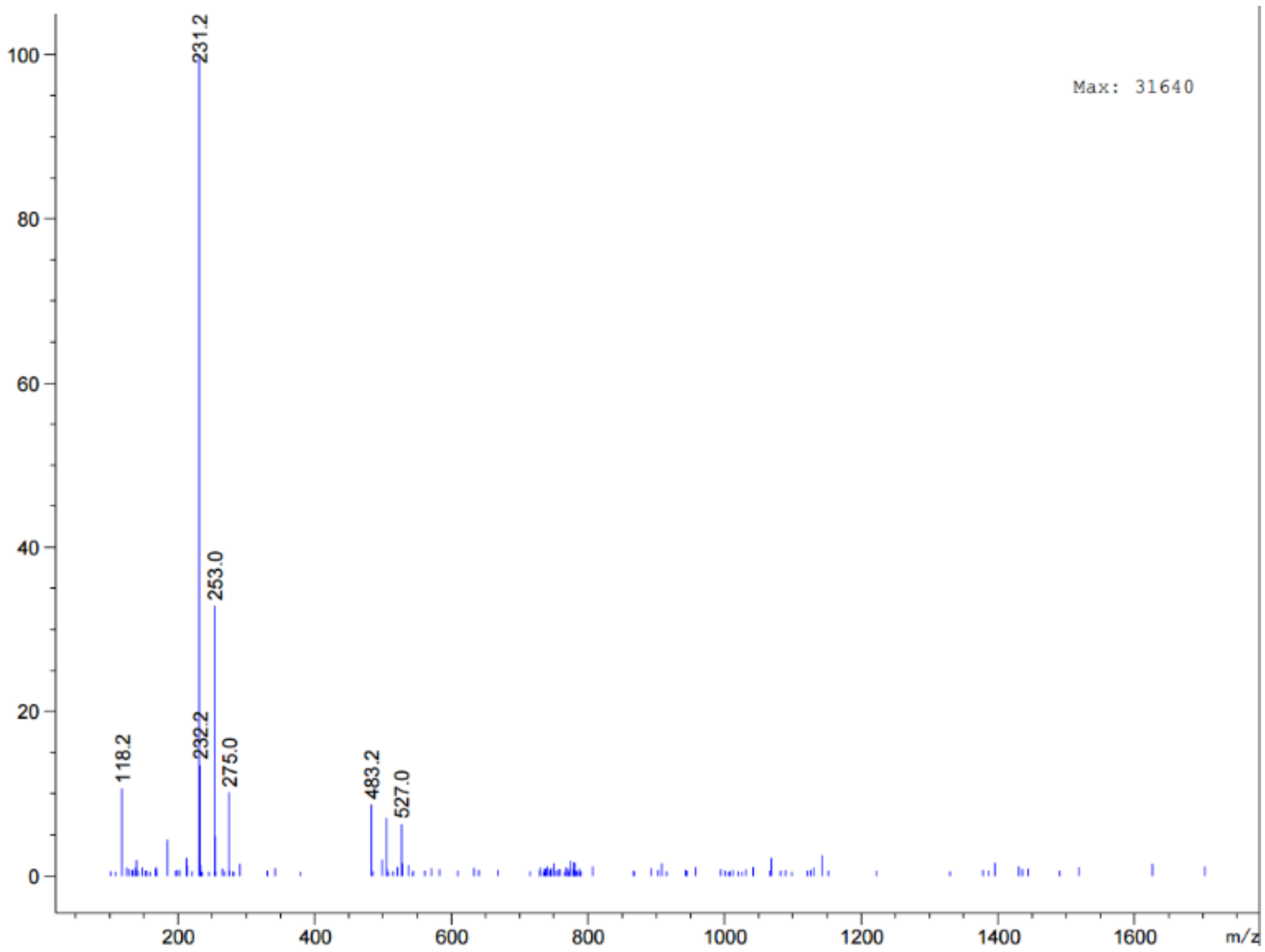

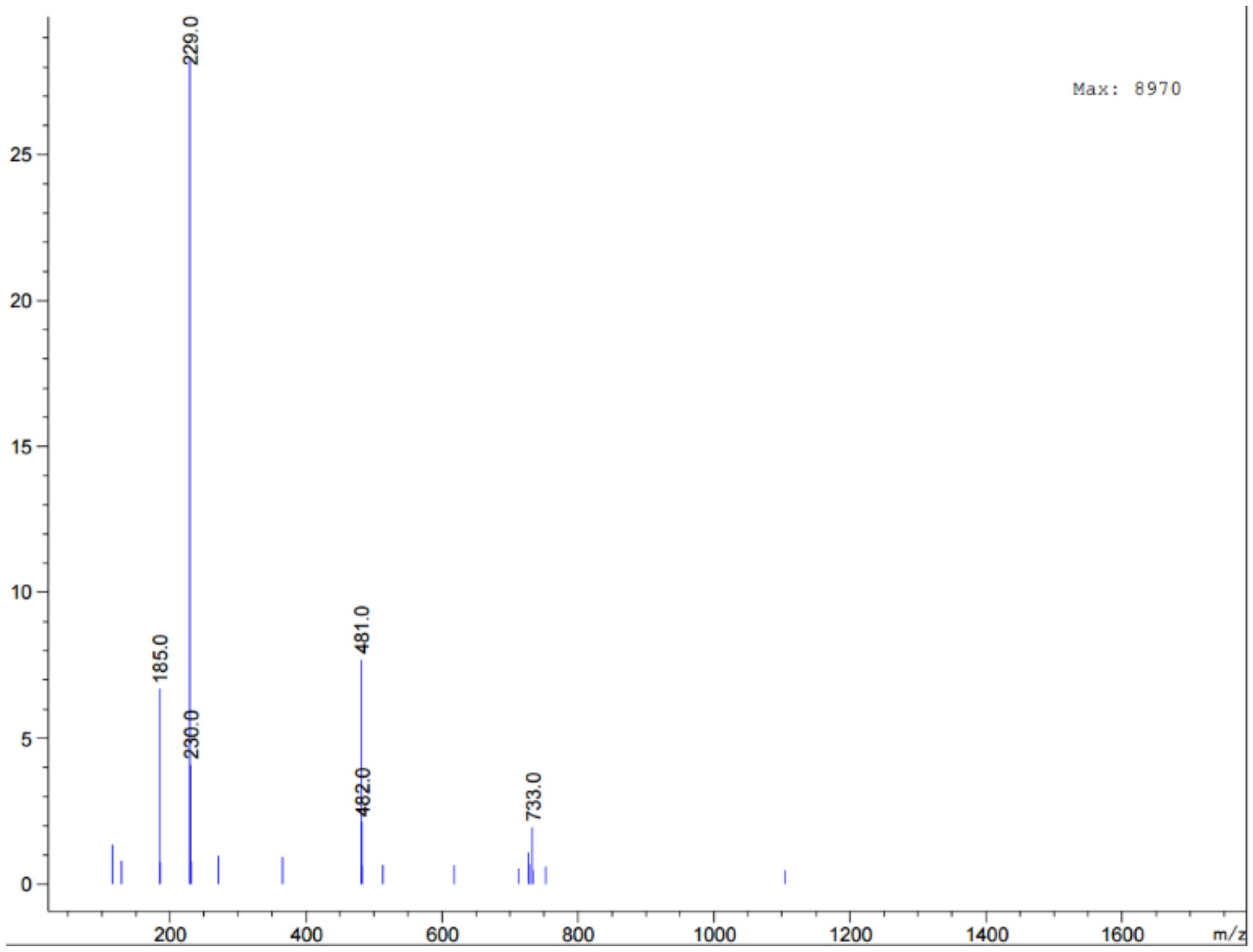

Figure S24: ESI mass spectrum of L-Ile-L-Val. Positive (top) and negative mode (bottom).

7. L-Ile-L-Leu spectroscopic data

^{1}H NMR (400 MHz, MeOD-*d_4*,) δ (ppm): 4.47 (m, 1H, CH_{α} Leu), 3.78 (d, *J* = 5.2 Hz, 1H, CH_{α} Ile), 1.97 (m, 1H, CH_{β} Ile), 1.73 (m, 1H, CH), 1.66 (m, 2H, CH), 1.61 (m, 1H, CH), 1.07 (d, *J*= 6.8 Hz, 3H, CH_3) 0.99-0.94 (m, 9H, 3xCH_3). MS (ESI): m/z 245.2 $[M+H]^+$. 243.0 $[M-H]^-$.

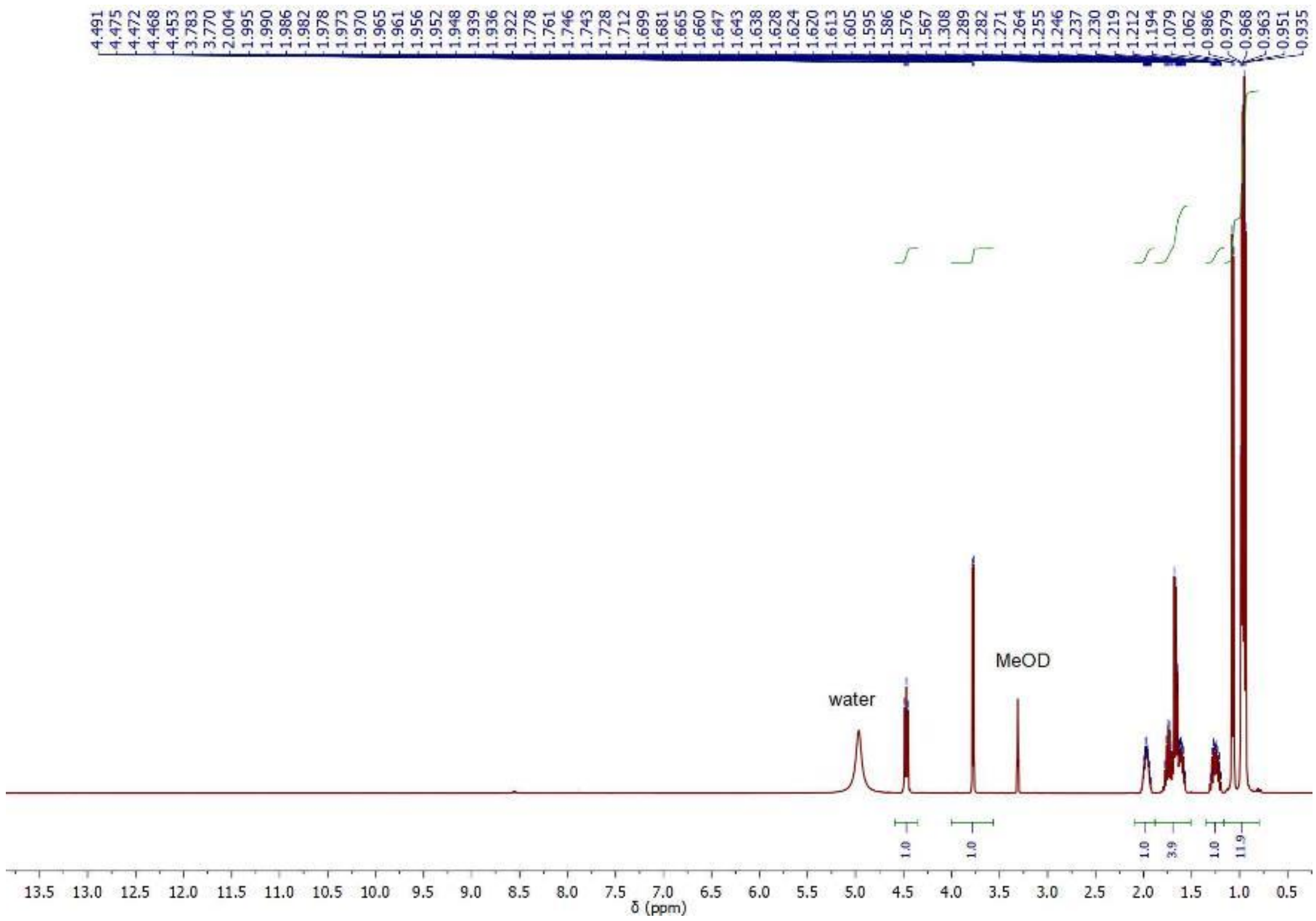

Figure S25: ^{1}H NMR (500 MHz, DMSO-d_6) of L-Ile-L-Leu.

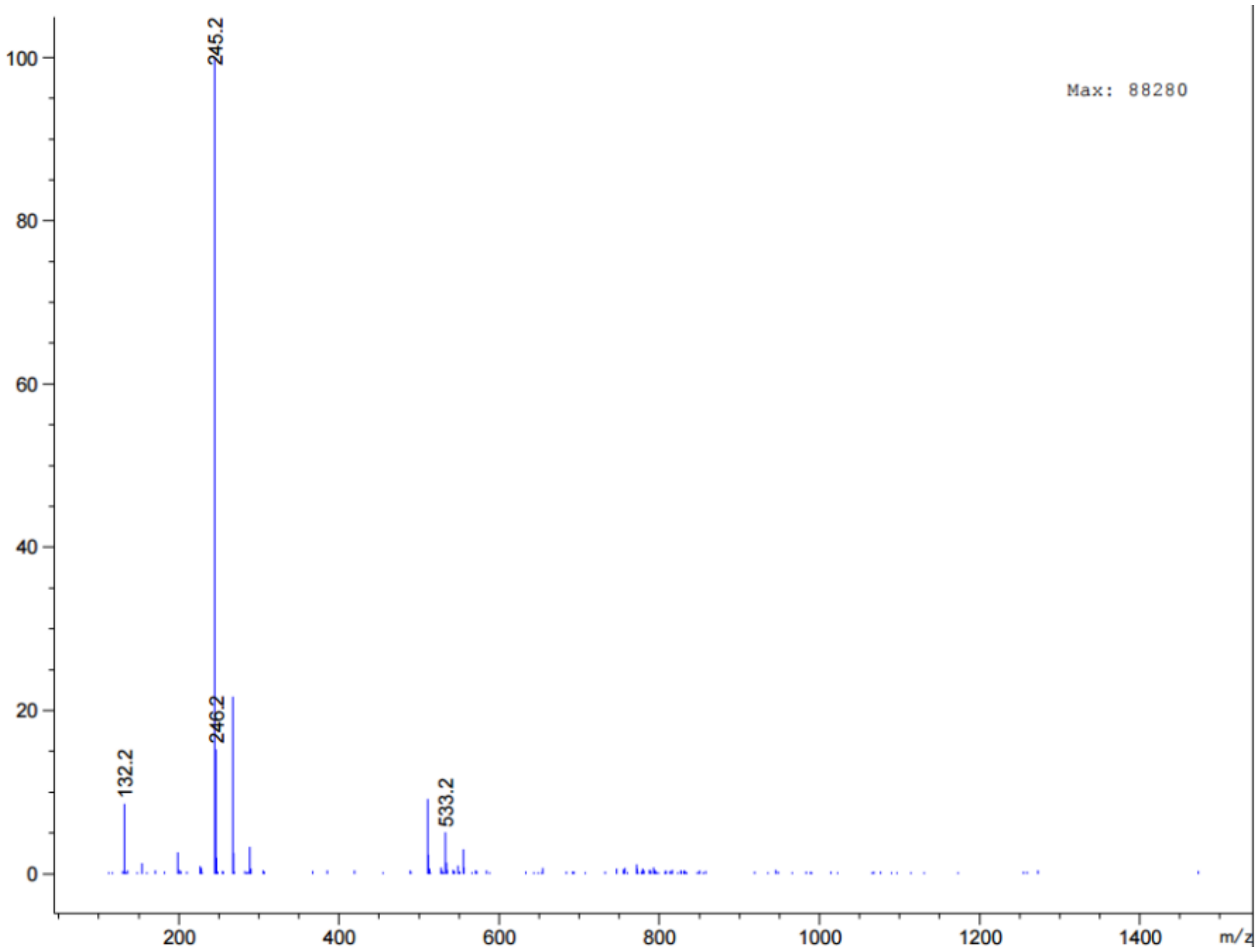

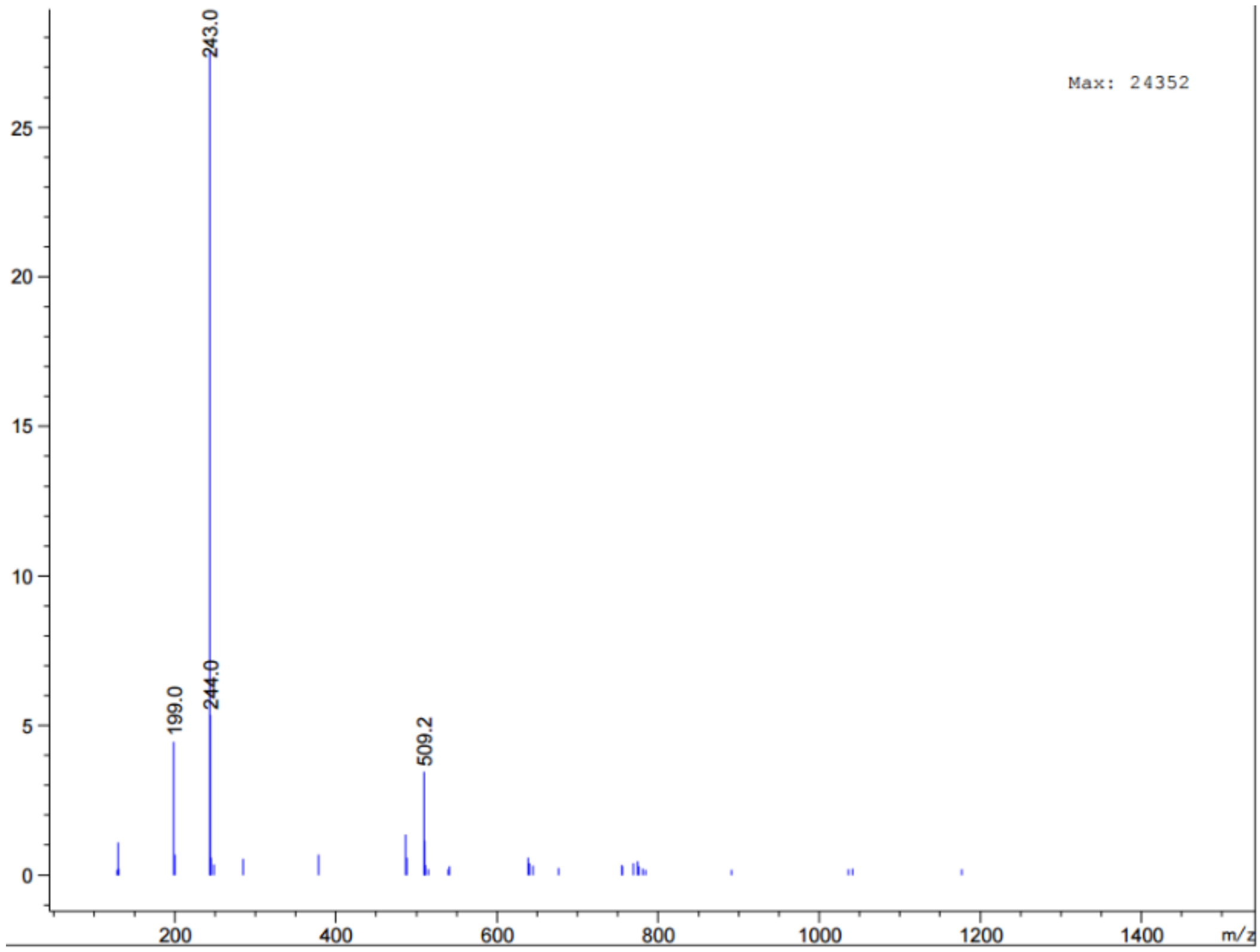

Figure S26: ESI mass spectrum of L-Ile-L-Leu. Positive (top) and negative mode (bottom).

8. L-Ile-L-Ile spectroscopic data

^{1}H NMR (400 MHz, DMSO-d_6,) δ (ppm): 8.56 (s (br), 3H, NH_3^+), 8.36 (d, J = 7.6 Hz, 1H, NH), 4.19 (dd, 1H, CH_α Ile), 3.61 (dd, J = 5.6, 5.6 Hz, 1H, CH_α Ile), 1.79 (m, 2H, 2xCH_β Ile), 1.46 (m, 1H, $CH_{2\gamma}$), 1.16 (m, 2H, $CH_{2\gamma}$ Ile), 0.88 (m, 12H, 2x$CH_{3\gamma}$, 2x$CH_{3\delta}$ Ile). MS (ESI): m/z 245.2 $[M+H]^+$. 243.0 $[M-H]^-$.

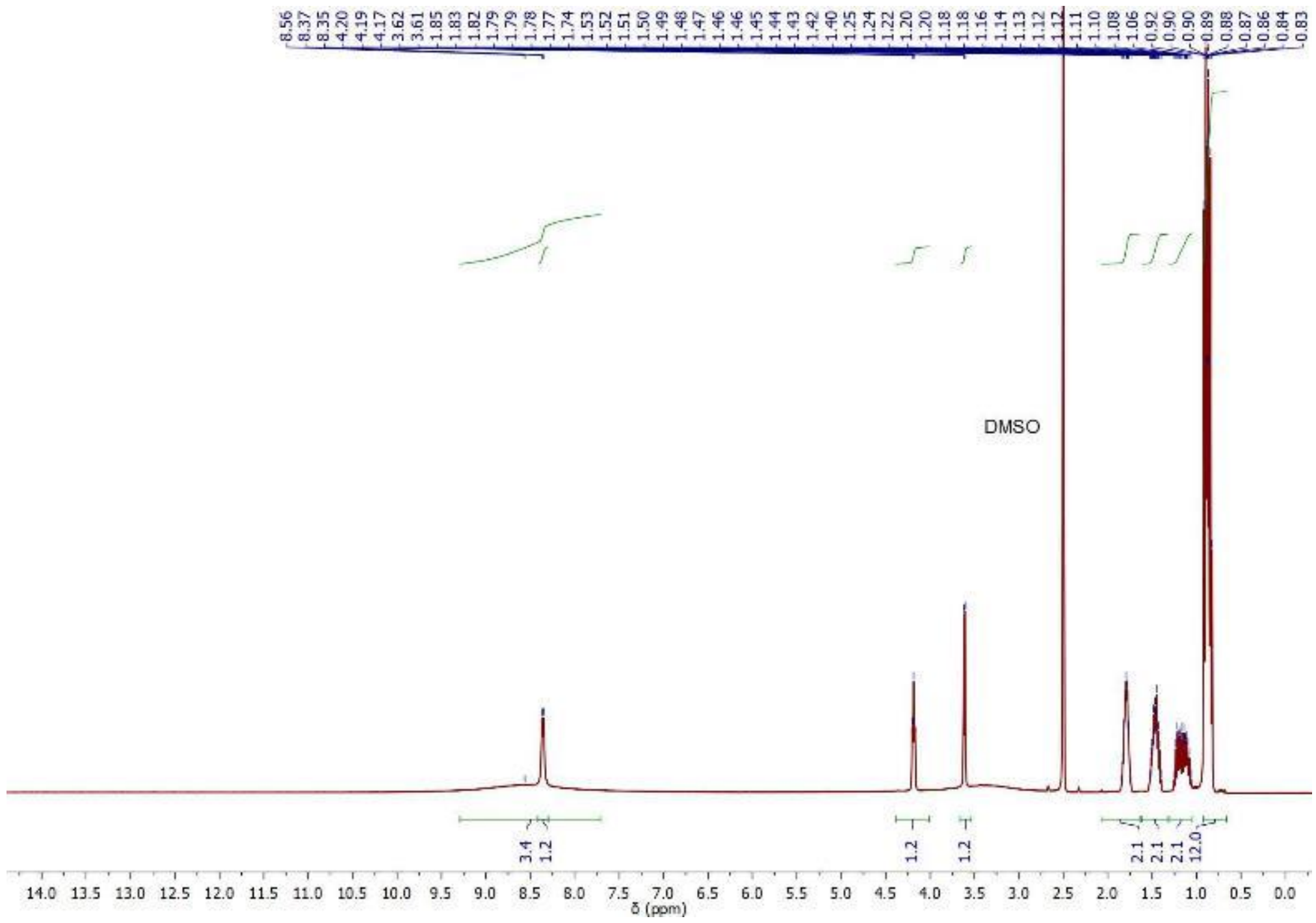

Figure S27: ^{1}H NMR (500 MHz, DMSO-d_6) of L-Ile-L-Ile

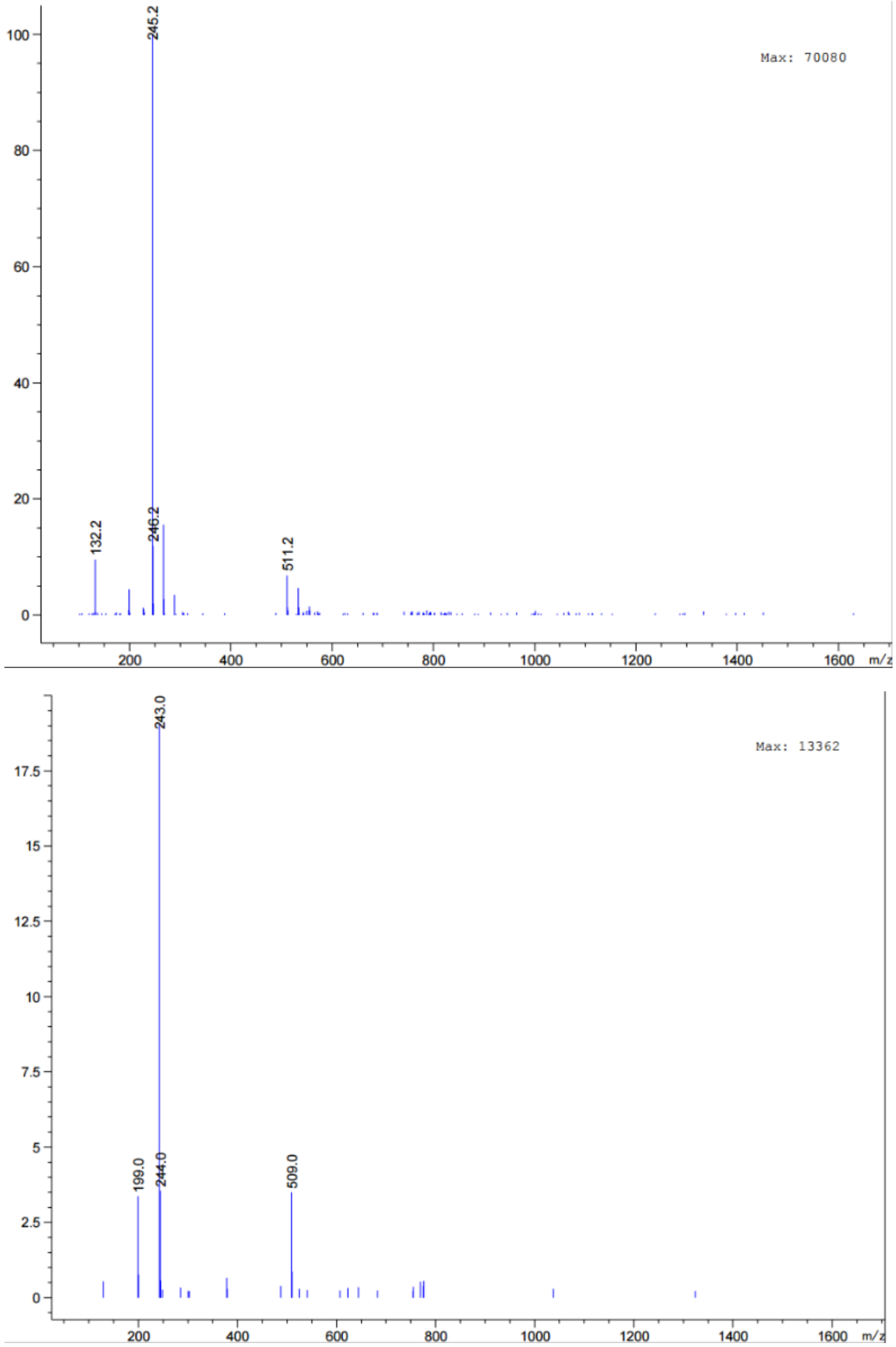

Figure S28: ESI mass spectrum of L-Ile-L-Ile. Positive (top) and negative mode (bottom).

9. HPLC traces

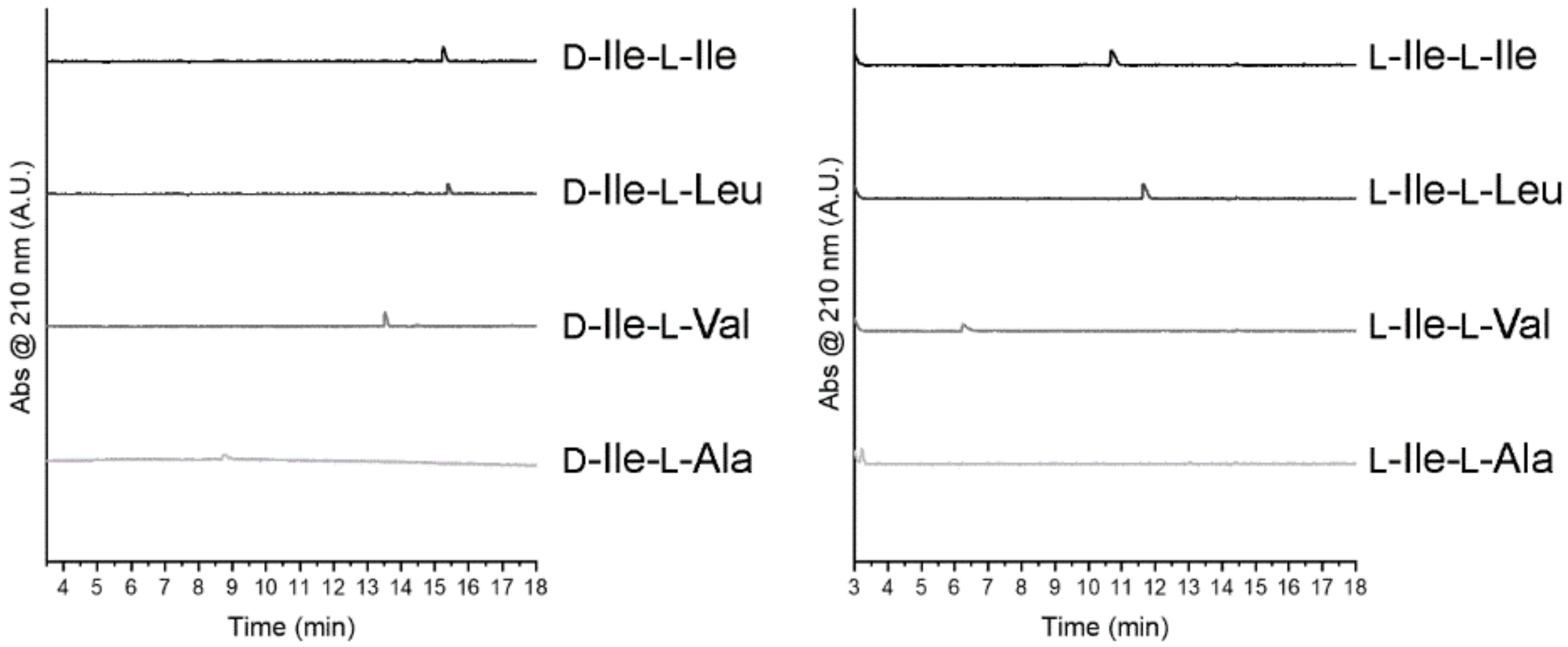

Figure S29: Reverse-phase HPLC traces of dipeptides.

10. Self-assembly tests

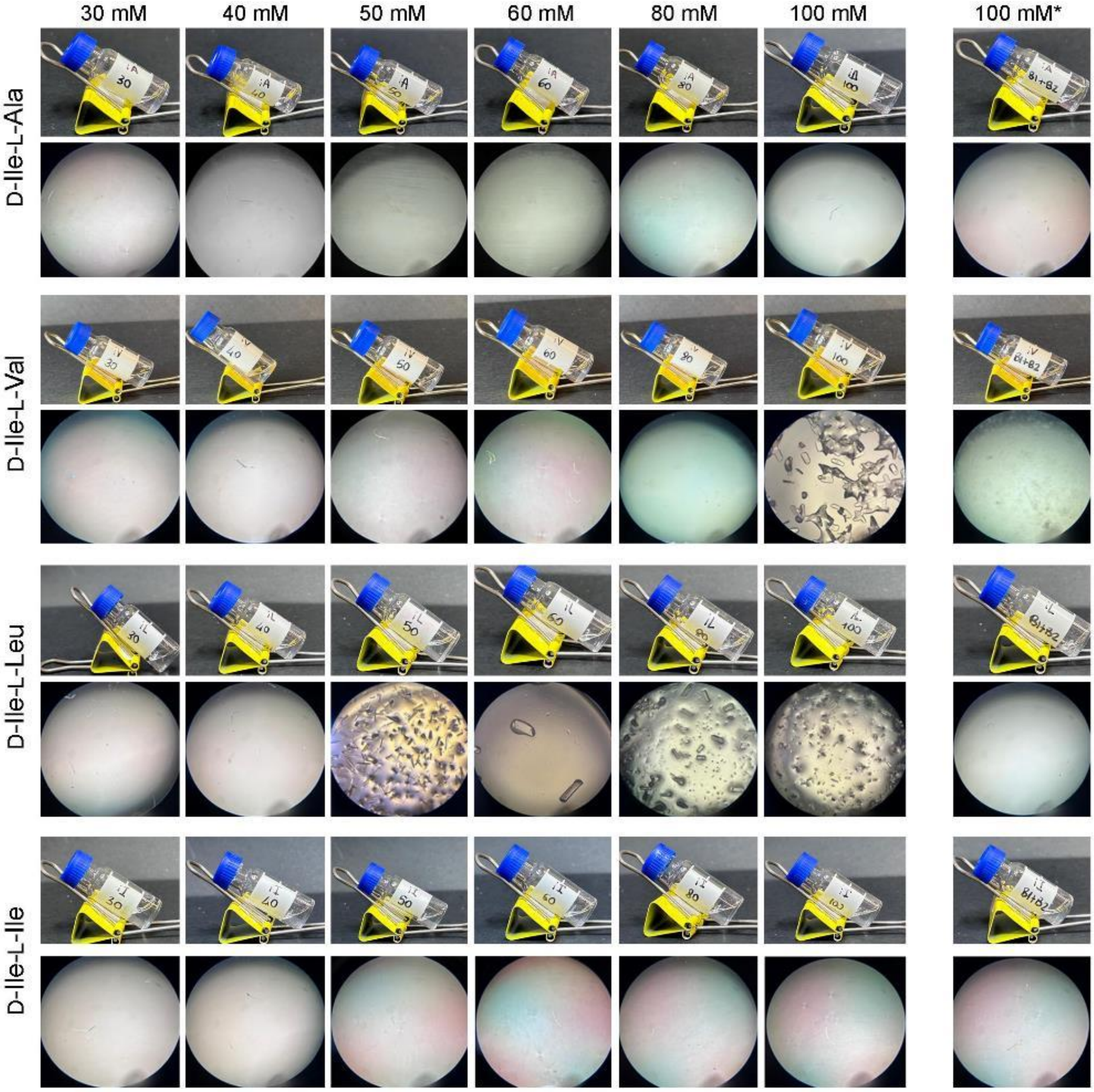

Figure S30: Photographs of self-assembly tests of heterochiral dipeptides at increasing concentrations in 0.1M PBS. * in sodium phosphate buffer 0.1M (pH trigger method).

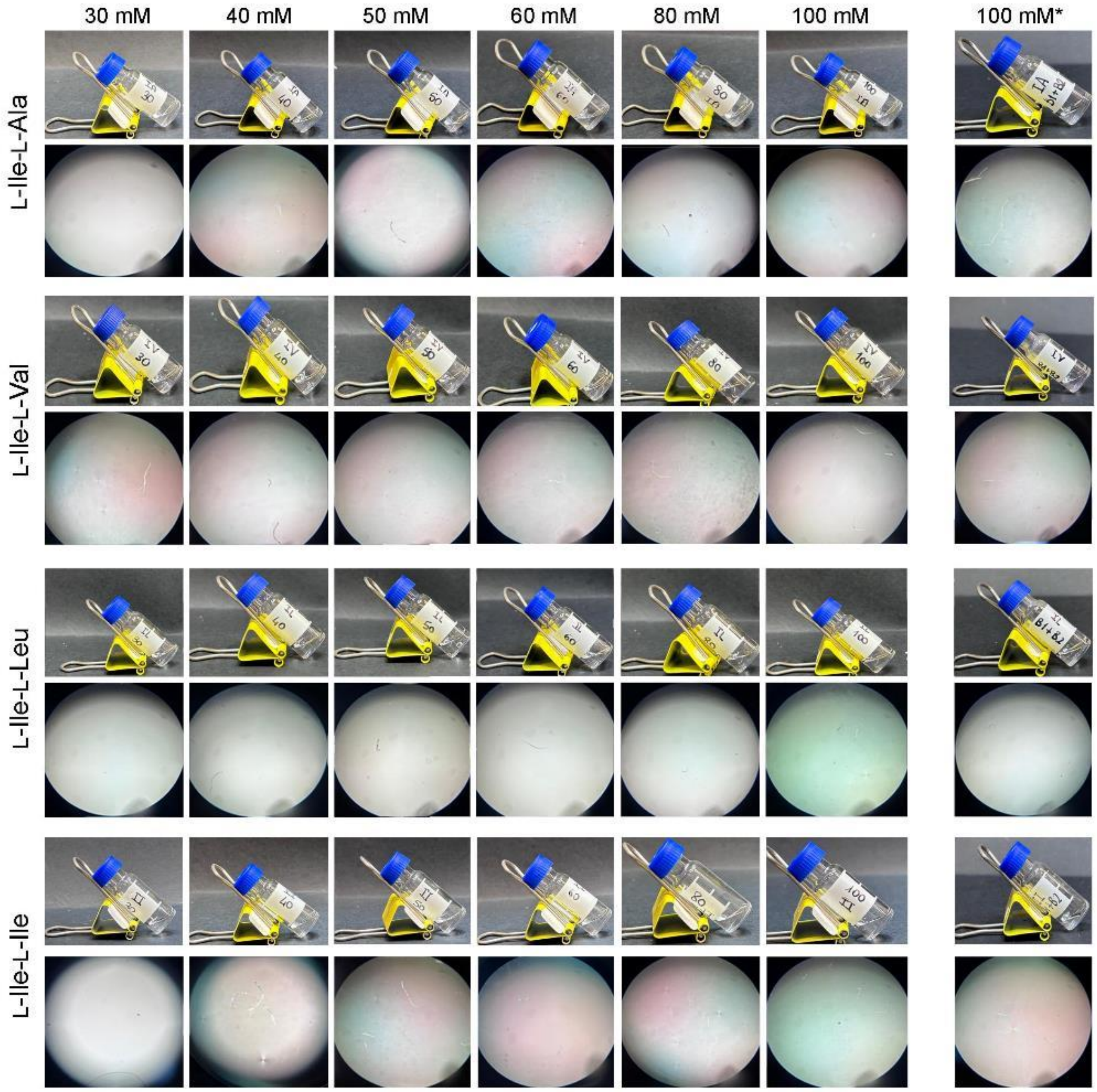

Figure S31: Photographs and optical microscope images of self-assembly tests of homochiral dipeptides at increasing concentrations in 0.1M PBS. * in sodium phosphate buffer 0.1M (pH trigger method).

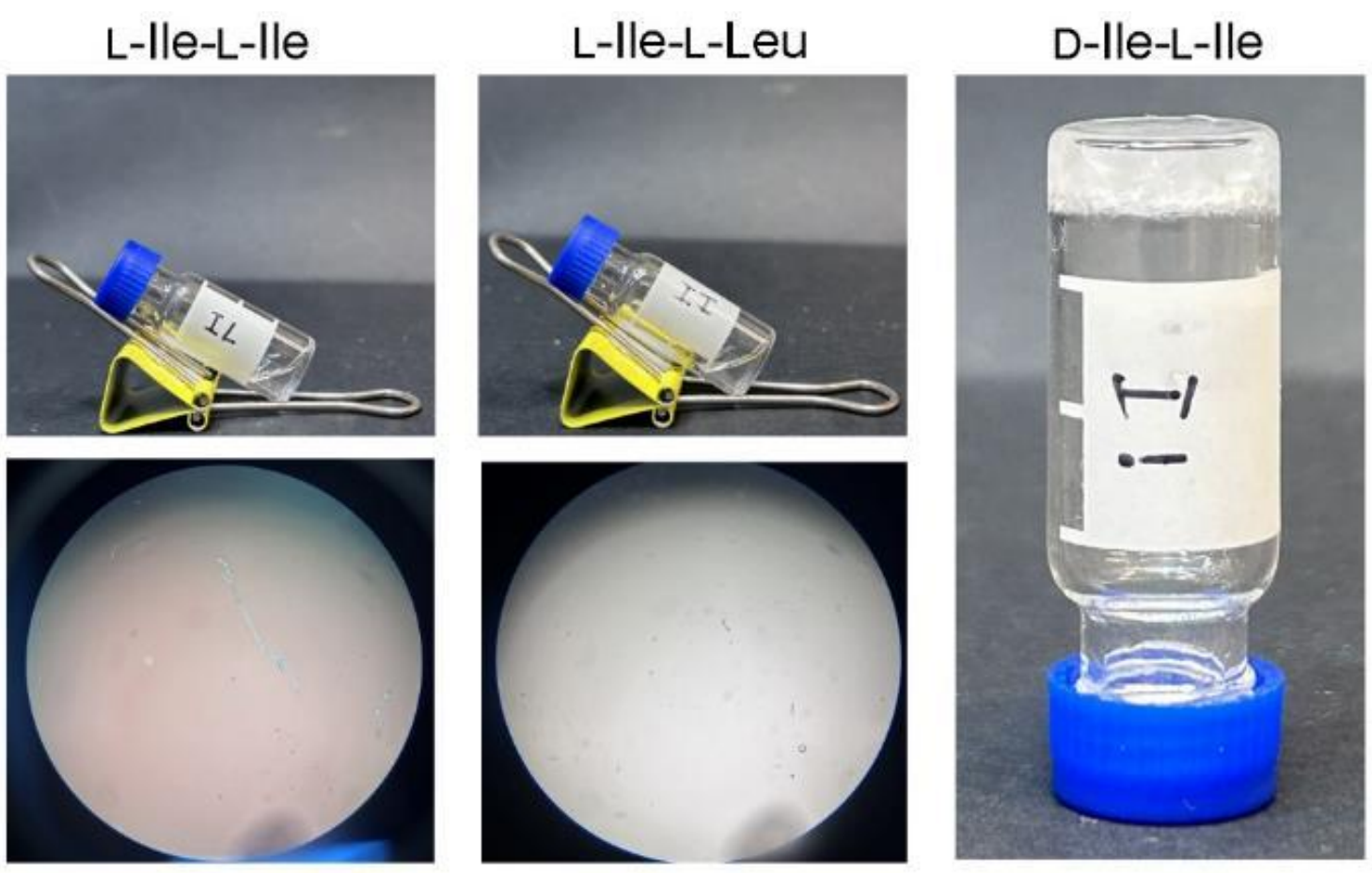

Figure S32: Photograph of self-assembly tests at 200 mM in 0.1M PBS.

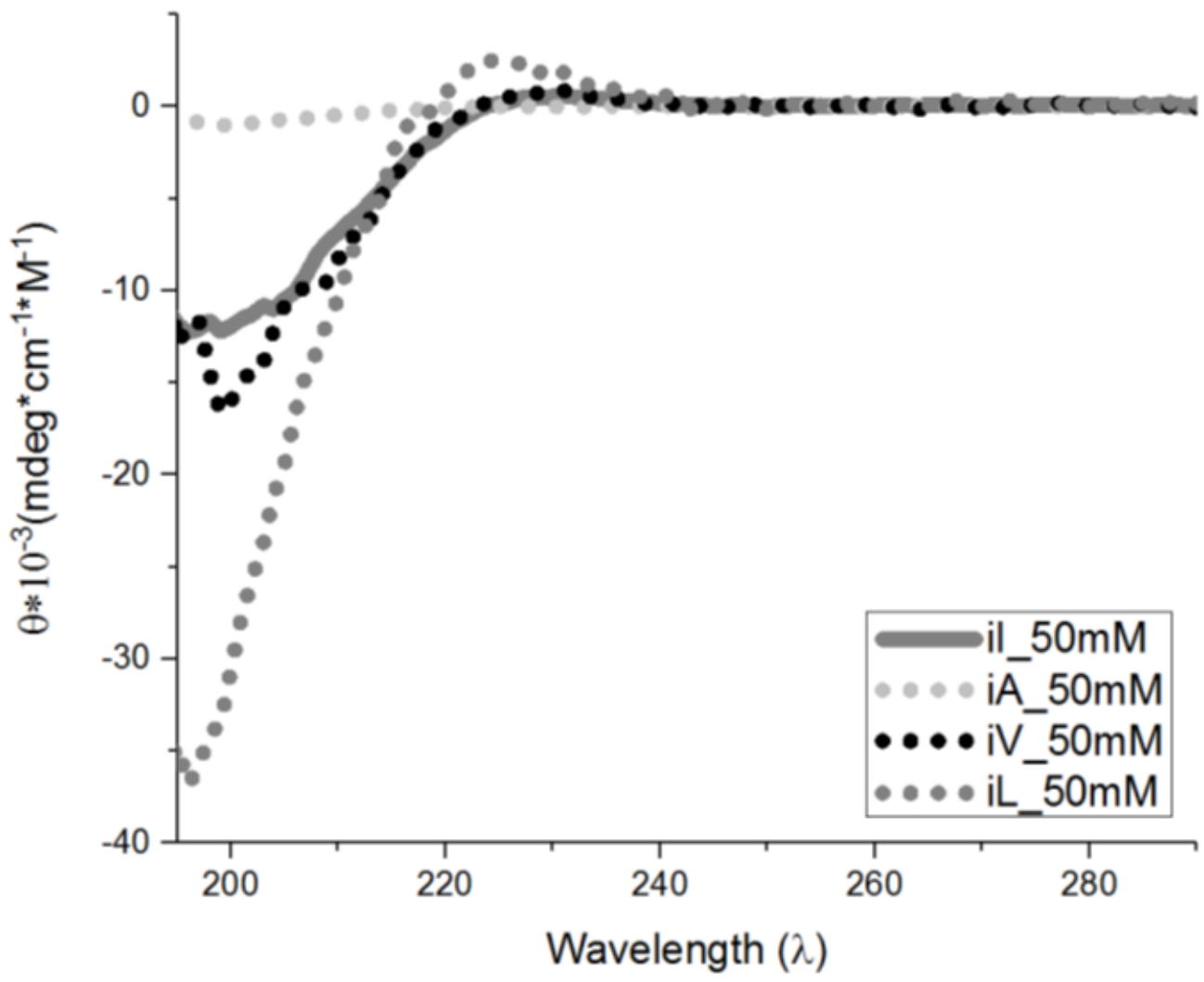

Figure S33: CD spectra of heterochiral dipeptides at 50 mM in milliQ water at pH 7.

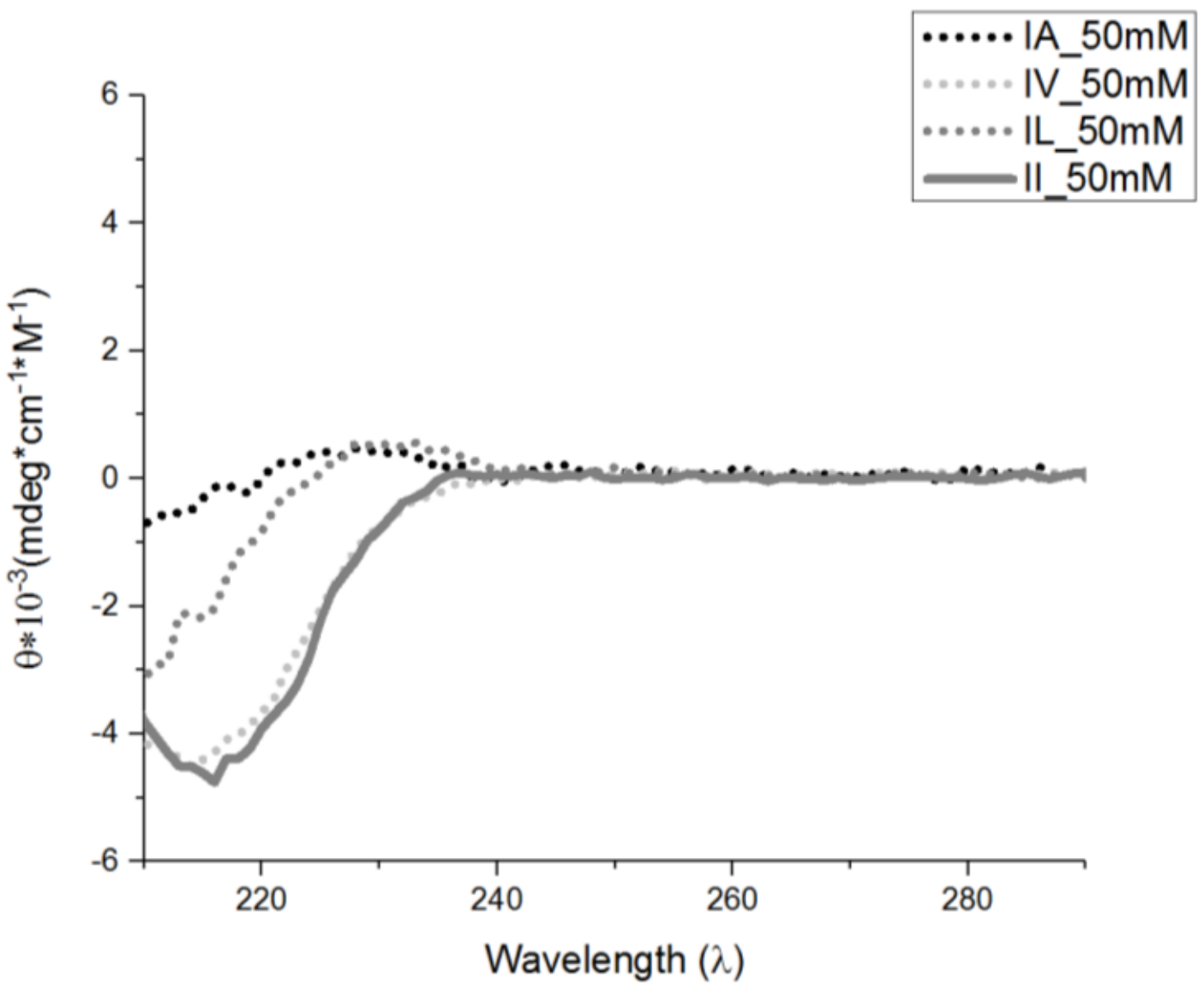

Figure S34: CD spectra of homochiral dipeptides at 50 mM in milliQ water at pH 7.

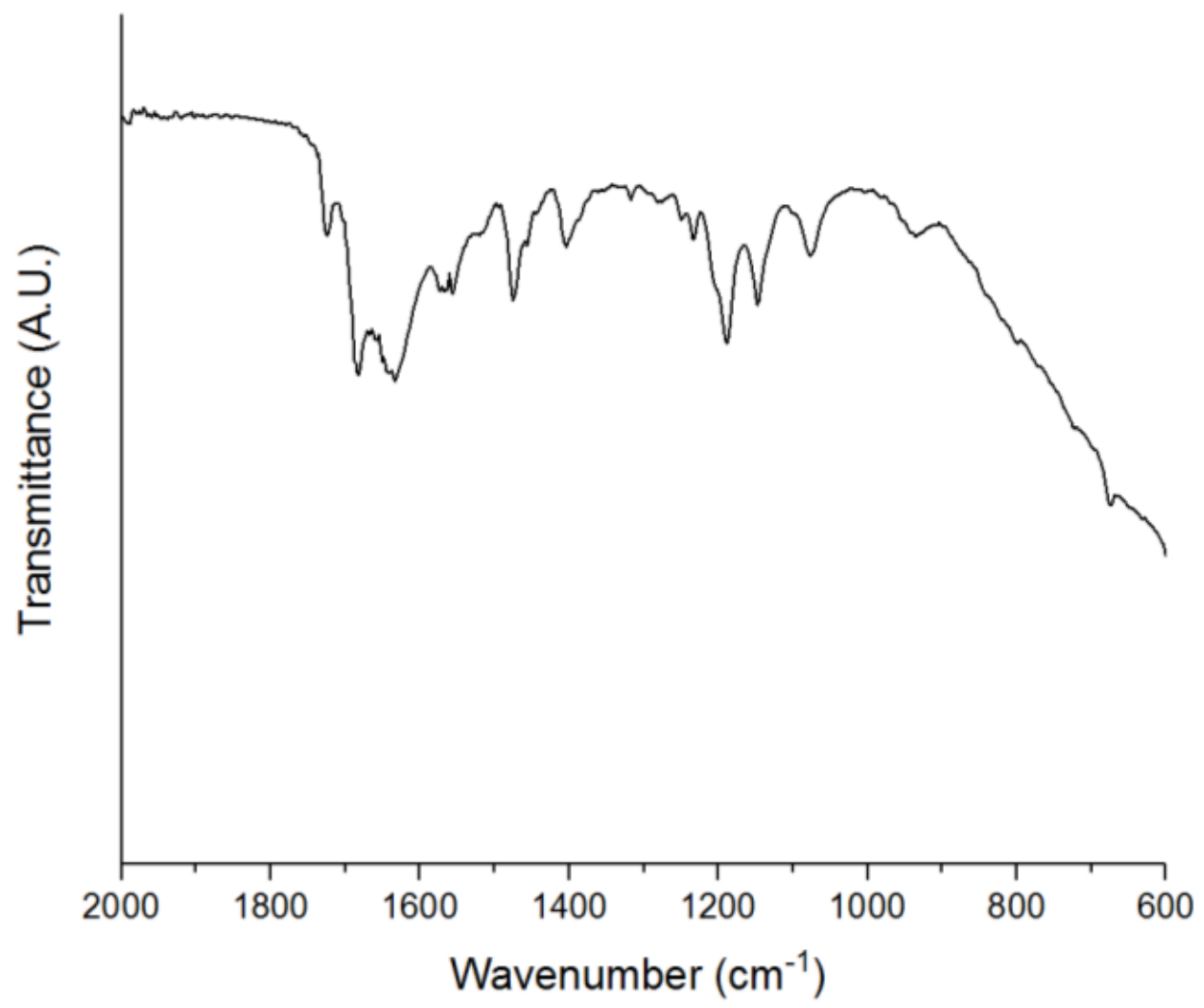

Figure S35: ATR-IR spectrum of the xerogel of iI at 200 mM in PBS.

11. Single-crystal X-ray diffraction

D-Ile-L-Ala

The peptide crystallised anhydrous in the monoclinic crystal system in the *C*2 space group. The asymmetric unit is composed by one zwitterionic independent conformer and no solvent molecules or counterions are present in the packing (Figure S36). The peptide is packed in alternate layers of hydrophobic regions constituted by the alkylic side chains and ionic polar regions. The overall charge of the peptide is 0, thus in absence of solvent, the peptide is packed head-to-tail to balance the electrical charge of the termini. Salt bridges are formed within layers and the head-to-tail interactions lead to a staggered peptide packing (Figure S37, A). This staggered disposition prevents the formation of amide hydrogen bonding. Salt bridges are also present between layers and no cavities are available for polar solvent molecules (Figure S37, B).

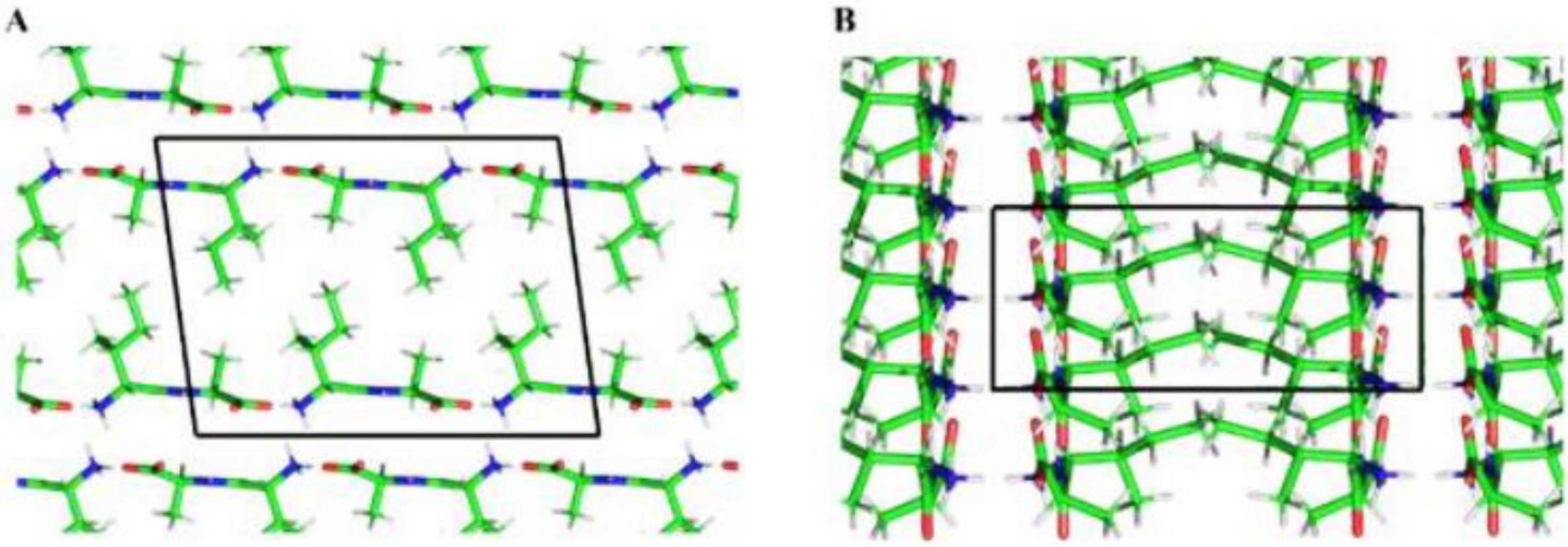

Figure S36: D-Ile-L-Ala crystal packing: **A** view along the *b* crystallographic axis, **B** view along the *a* crystallographic axis. Peptide is represented as stick model. Green: carbon, blue: nitrogen, red: oxygen, white: hydrogen.

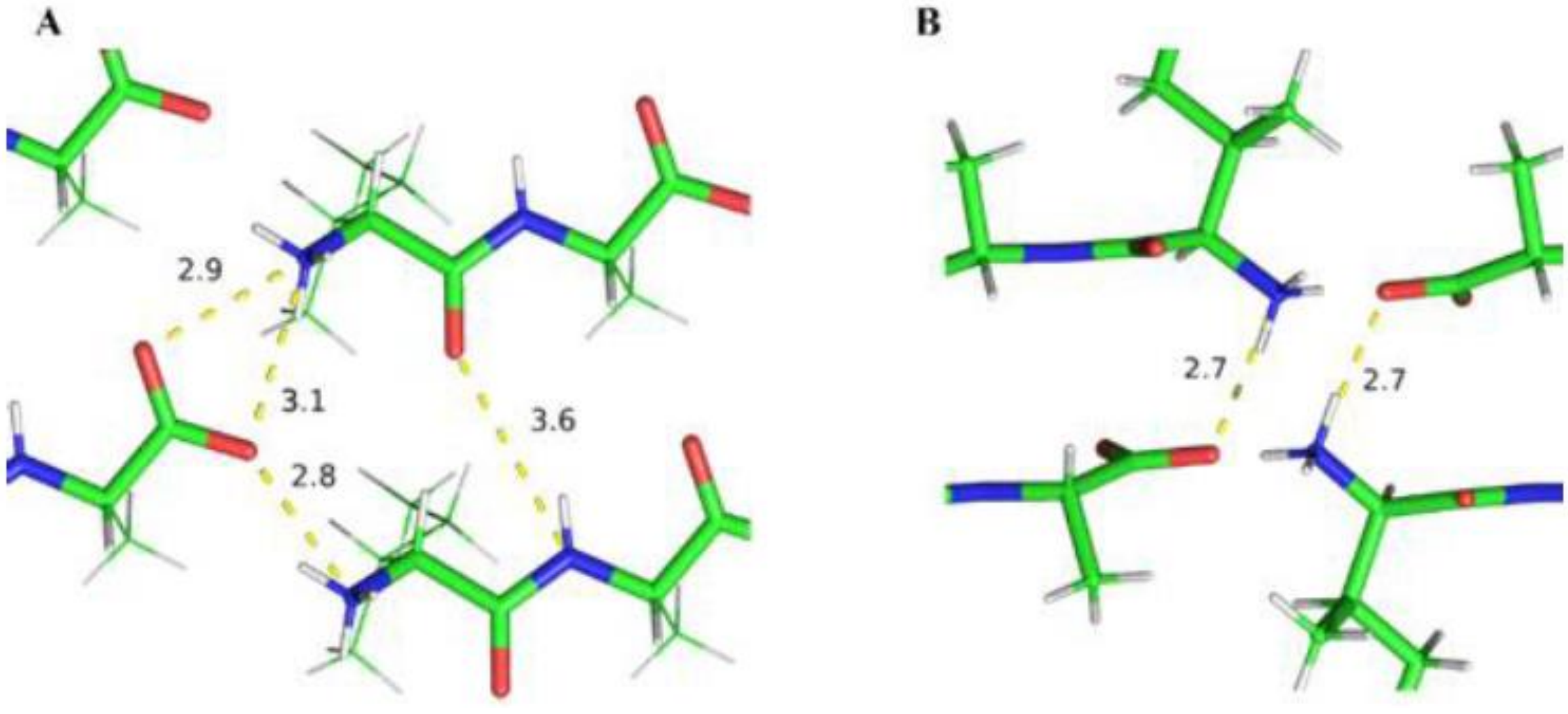

Figure S37: D-Ile-L-Ala noncovalent polar interactions. A: One layer is composed by staggered peptides which interact by head-to-tail salt bridges between termini. The amide groups of neighbouring peptides are too distant to engage in hydrogen bond interactions. Side chains are represented as wire model for clarity. B: Layers are connected in the polar region by more salt bridges between neighbouring termini.

D-Ile-L-Val

The peptide crystallised in the monoclinic crystal system in the *C*2 space group. The asymmetric unit is composed by one independent peptide conformer and three water molecules. No counterions are present and the peptide crystallised as zwitterion. The peptide packed in alternate hydrophobic/hydrophilic layers. Hydrophobic layers are composed by the peptide alkylic side chains. The hydrophilic layers are constituted by the polar peptide termini and water molecules (Figure S38). No ionic interactions are present in the polar layer. The three water molecules act as bridges between the polar groups and only ionic-dipole and dipole-dipole interactions are present (Figure S39). One peptide layer is formed by staggered peptides in a head-to-tail fashion with all the three water molecules acting as bridge between the polar functional groups. The C- and N-termini of the staggered peptide chains are bridged by two water molecules (Figure S39, A, red oxygen). Amide groups are bridged by the third water molecule (Figure S39, A, cyan oxygen) which also bridges the two charged termini of the same peptide unit. The two neighbouring peptide layers are held by the remaining hydrogens of the three water molecules (Figure S39, B).

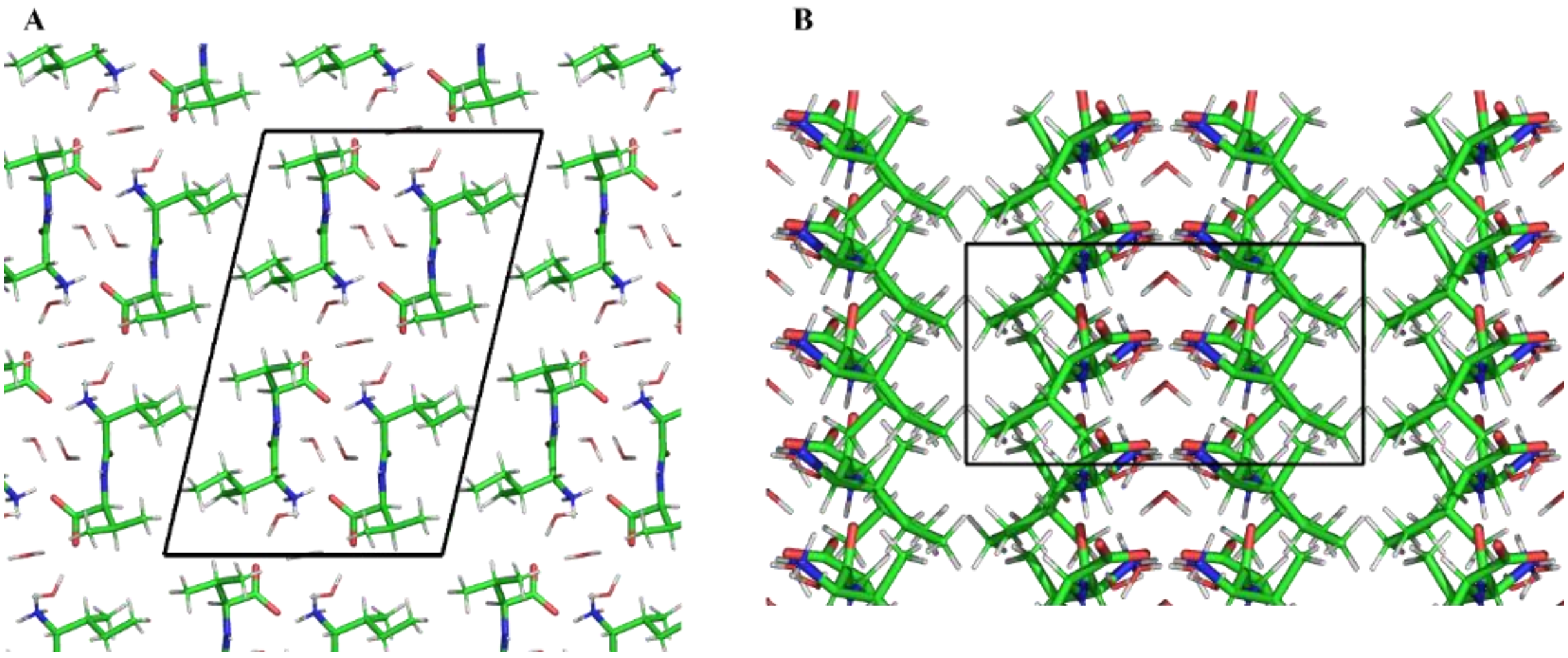

Figure S38: D-Ile-L-Val crystal packing: **A** view along the *b* crystallographic axis, **B** view along the *a* crystallographic axis. Peptide is represented as stick model. Green: carbon, blue: nitrogen, red: oxygen, white: hydrogen.

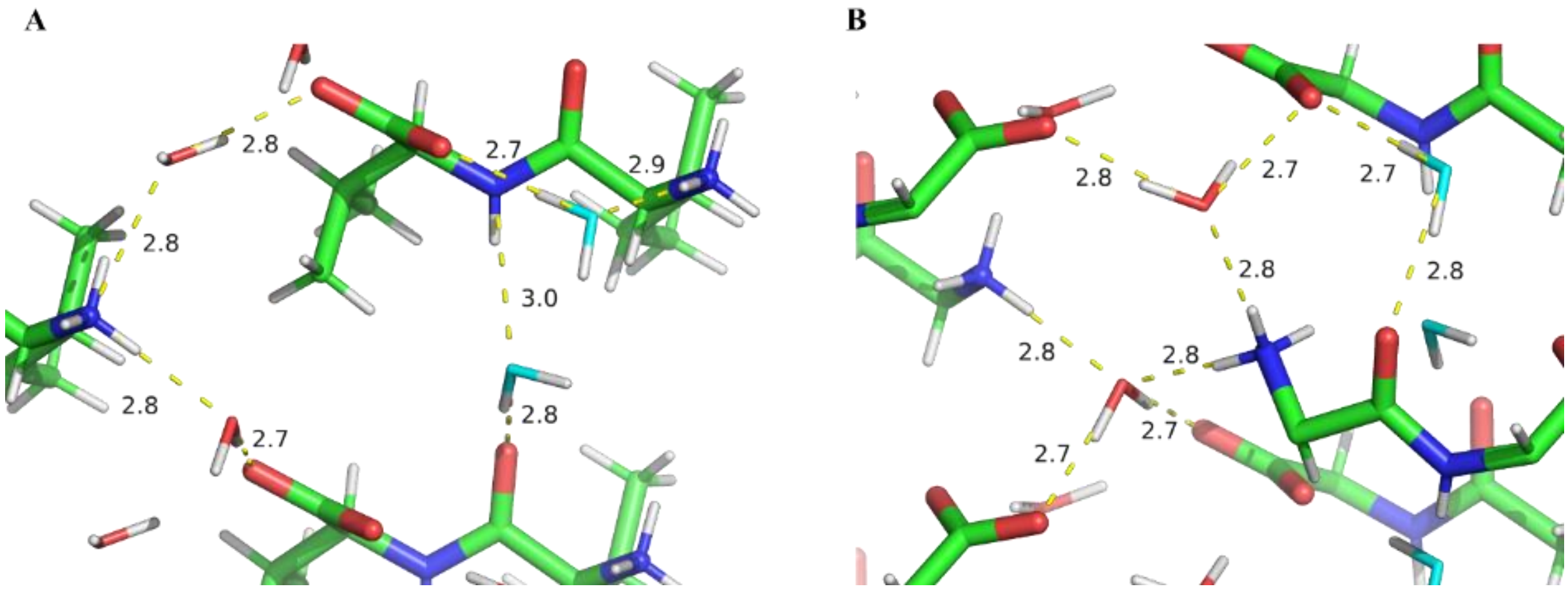

Figure S39: D-Ile-L-Val hydrogen bond network. One peptide chain and three water molecules are present in the asymmetric unit. **A**: one peptide layers is composed by a staggered peptide packing. Two water molecules (red oxygen) act as a bridge between different peptide units whereas one water molecule (cyan oxygen) is involved in a intramolecular bridge between the termini of the same peptide. This water molecule further interacts as a hydrogen bond donor with the amide carbonyl of the neighbouring peptide layer. **B**: The peptide layers are bridged by all the water molecules. Two water molecules (red oxygen) bridge carboxylates and ammonium groups between the peptidic layers. The water molecule in the special *C*2 position interact with four different peptide termini in a tetrahedral geometry. The cyan water molecule bridges the carboxylate group present in one layer with the amide carbonyl group of the neighbouring peptide layer. In panel **B**, side chains are omitted for clarity.

D-Ile-L-Leu

The peptide crystallised in the monoclinic crystal system in the *C*2 space group. The asymmetric unit is composed of one zwitterionic peptide molecule and three water molecules. The packing is similar to D-Ile-L-Val. The peptide is packed in alternate hydrophobic/hydrophilic layers (Figure S40). The hydrophobic layer is composed by the packed alkylic side chains of the peptides. The hydrophilic layer is formed by the polar charged N- and C-terminal residues of the peptide and ordered water molecules. Water molecules bridge all the polar residues of the peptide through a hydrogen bond network (Figure S41). The polar interactions are ionic-dipole and dipole-dipole between the solvent and the peptide. No direct polar non-covalent interactions between peptides are present in the crystal.

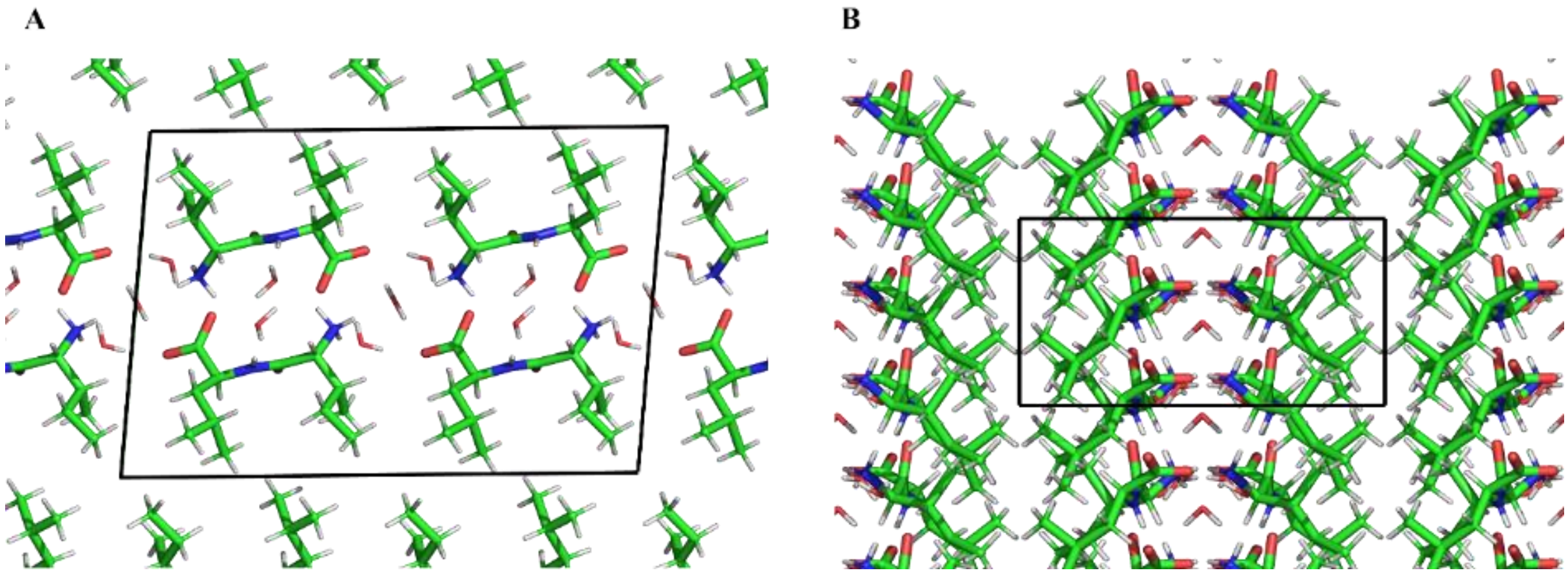

Figure S40: D-Ile-L-Leu crystal packing: **A** view along the *b* crystallographic axis, **B** view along the *a* crystallographic axis. Peptide is represented as stick model. Green: carbon, blue: nitrogen, red: oxygen, white: hydrogen.

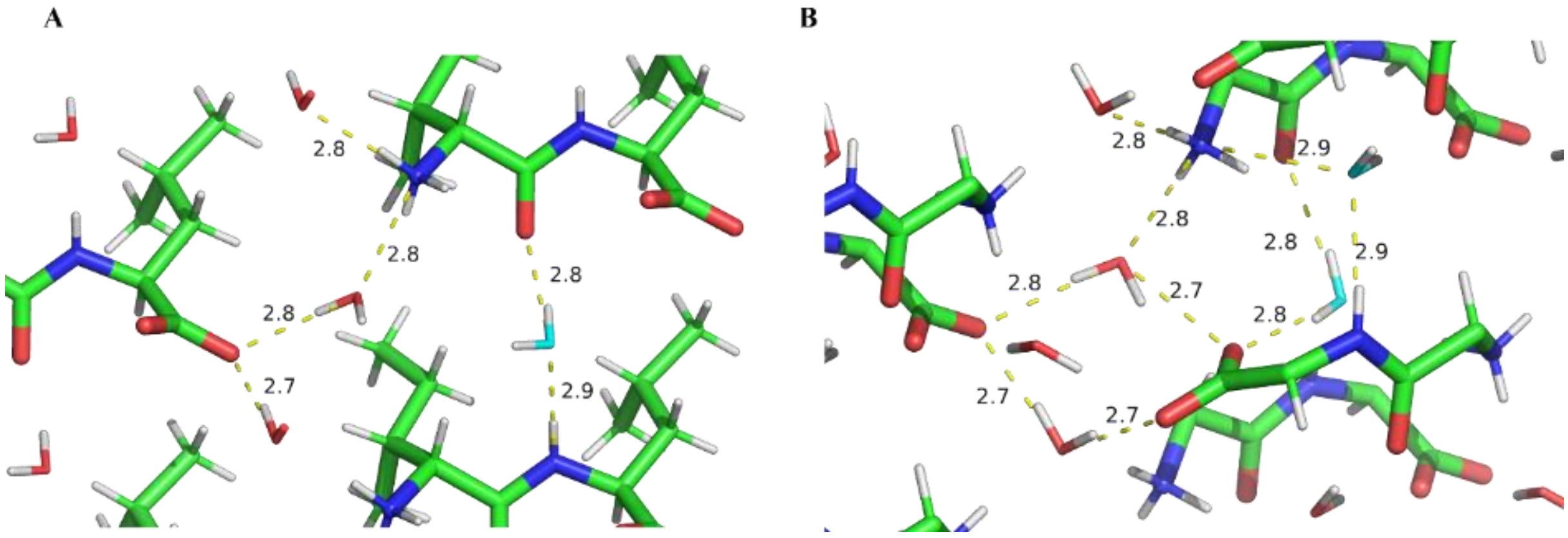

Figure S41: D-Ile-L-Leu hydrogen bond network. One peptide chain and three water molecules are present in the asymmetric unit. **A:** The peptide layer shows a disposition similar to D-Ile-L-Val. Water molecules bridge the polar termini between peptide chains. The cyan water molecule bridge amide groups of neighbouring peptides, thus there is no direct interaction between different amides. **B:** The two peptide layers are bridged by all the water molecules present in the asymmetric unit. The cyan water molecule bridges intramolecularly the N- and C-terminal residue of the peptide. In panel **B**, side chains are omitted for clarity.

D-Ile-L-Ile

The peptide crystallised in the monoclinic crystal system in the $P2_1$ space group. The asymmetric unit is composed by two independent zwitterionic conformers of the peptide and one molecule of DMSO. The peptide is packed forming head-to-tail salt bridges between the C- and N-terminal groups (Figure S42). The DMSO

molecule interacts both with the polar groups and with the alkylic side chains of the peptide. No hydrophilic regions are present in the crystal. The peptide forms a hydrogen bond network between its polar functional groups and the oxygen of the DMSO (Figure S43). Both the independent conformers form salt bridges and interact with the DMSO molecule. DMSO bridges the ammonium groups of the two independent conformers. The NH amide group of the cyan conformer engages in a hydrogen bond with the carboxylate of the neighbouring cyan peptide. This interaction is not observed for the green conformer and its amide group does not form any hydrogen bond.

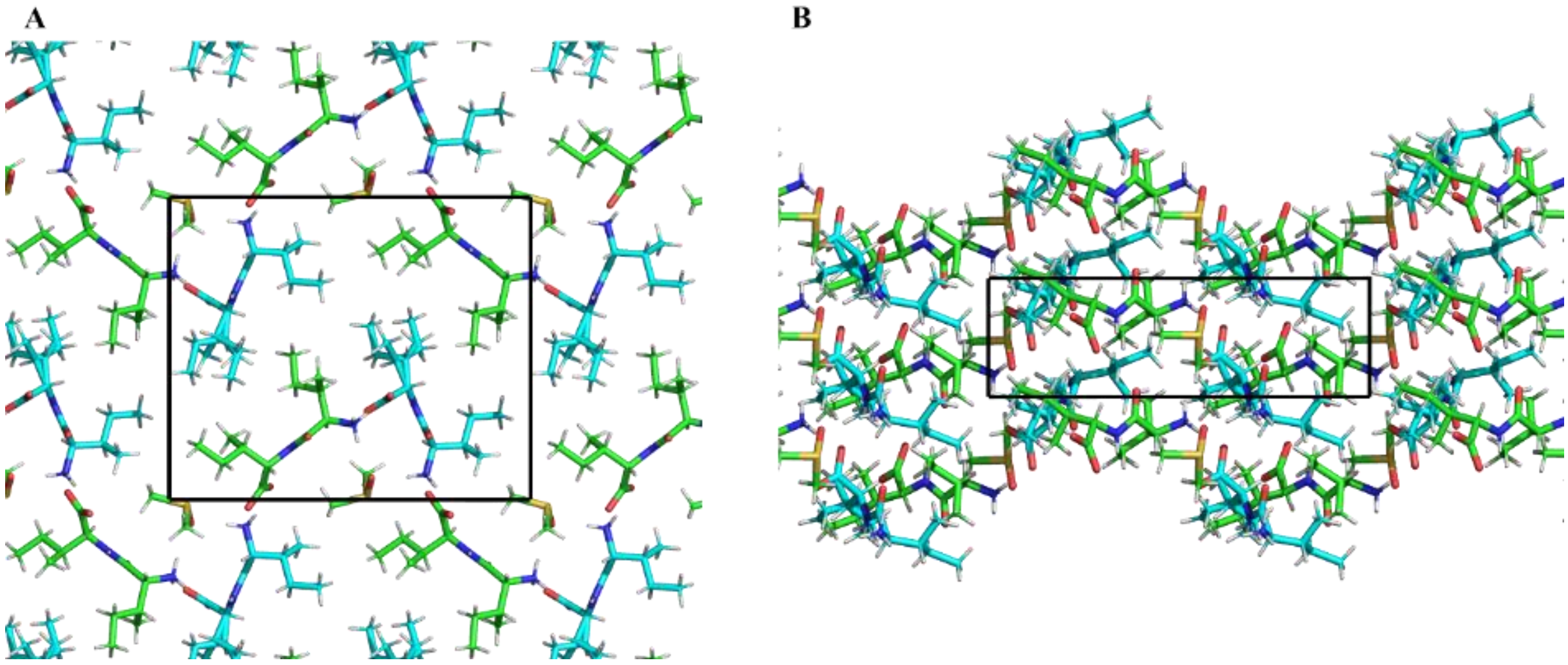

Figure S42: D-Ile-L-Ile crystal packing: **A** view along the *a* crystallographic axis, **B** view along the *c* crystallographic axis. Peptide is represented as stick model. Green, cyan: carbon, blue: nitrogen, red: oxygen, yellow: sulfur, white: hydrogen.

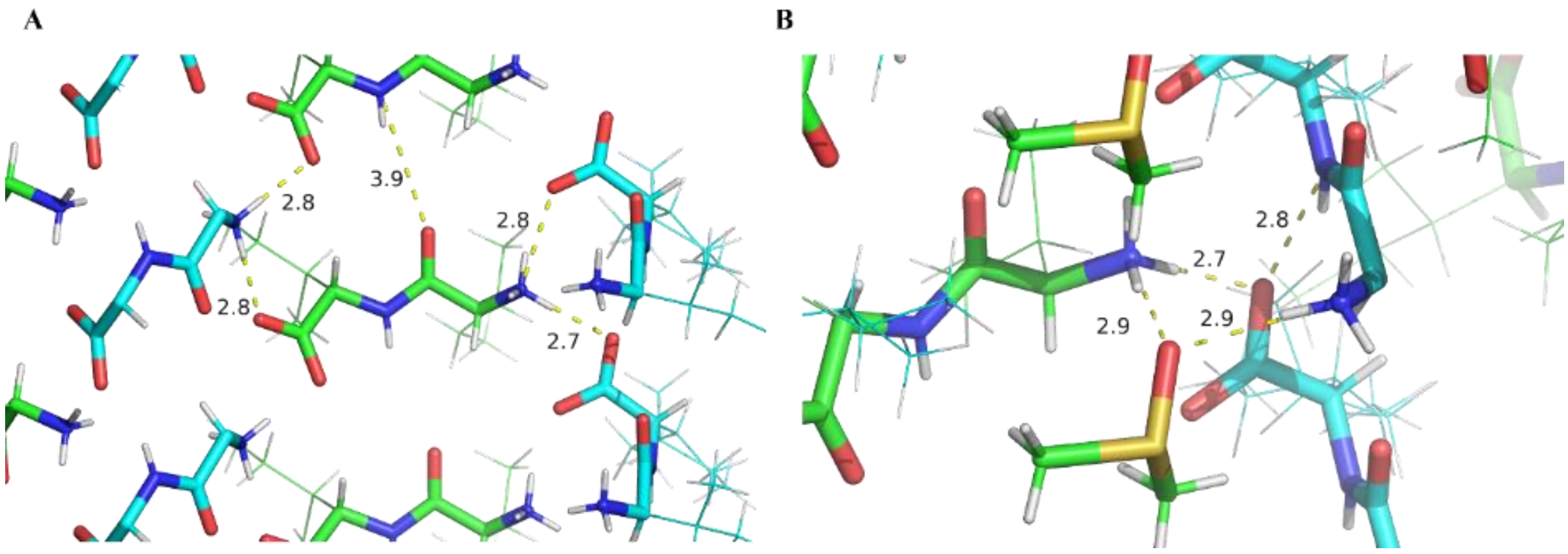

Figure S43: D-Ile-L-Ile Hydrogen bond network. **A:** Head-to-tail salt bridges between peptides. Amide group of the green peptide is isolated, and it is not involved in hydrogen bond. DMSO is omitted for clarity. Side chains are either omitted or represented by wire model clarity **B:** DMSO acts as a hydrogen bond acceptor bridging the two N-terminal residues of the two independent peptide conformers. The NH amide group of the cyan peptide engages in a hydrogen bond interaction with the carboxylate group of the neighbour cyan peptide.

Crystallographic data

Table S1. Crystallographic data and refinement details.

	**D-Ile-L-Ala**	**D-Ile-L-Val**	**D-Ile-L-Leu**	**D-Ile-L-Ile**
CCDC	2476795	2476794	2477381	2476796
Formula	$C_9H_{18}N_2O_3$	$C_{11}H_{22}N_2O_3$, 2.5 H_2O	$C_{12}H_{24}N_2O_3$, 2.5 H_2O	2($C_{12}H_{24}N_2O_3$), $C_2H_6O_1S_1$
Temperature (K)	100	100	100	100

Wavelength (Å)	0.7	0.7	0.7	0.7
Crystal system	Monoclinic	Monoclinic	Monoclinic	Monoclinic
Space group	*C*2	*C*2	*C*2	$P2_1$
a (Å)	16.672(3)	19.228(4)	19.352(4)	5.7010(11)
b (Å)	5.1930(10)	6.5710(13)	6.5710(13)	18.209(4)
c (Å)	12.480(3)	12.268(3)	13.009(3)	15.351(3)
α (°)	90	90	90	90
β (°)	97.49(3)	103.15(3)	95.15(3)	93.63(3)
γ (°)	90	90	90	90
V (Å^3)	1071.3(4)	1509.4(6)	1647.6(6)	1590.4(5)
Z, ρ_{calc} (g/cm^3)	4, 1.254	4, 1.212	4, 1.167	2, 1.184
μ (mm^{-1})	0.09	0.092	0.088	0.14
F (000)	440	604	636	620
Data collection θ range	1.621 - 29.996	1.679 – 30.004	1.548 – 29.617	1.309 – 30.062
Refl. Collected / unique	9868 / 3214	14699 / 4581	15283 / 4783	30396 / 9570
R_{int}	0.0852	0.0846	0.0778	0.0535
Completeness (%)	99.4	99.1	97.3	99.3
Data/Restraints/Parameters	3214 / 1 / 132	4581 / 1 / 183	4783 / 1 / 165	9570 / 1 / 385
GooF	1.092	1.039	0.981	1.097
*R*1, w*R*2 [I>2σ(I)]	0.0642 / 0.1732	0.0601 / 0.148	0.0623 / 0.1587	0.0444 / 0.1203
*R*1, w*R*2 all data	0.0837 / 0.1931	0.0911 / 0.1731	0.0623 / 0.1652	0.0523 / 0.1325

Table S2. Values of χ_1 and χ_2 torsion angles of the isoleucine side chains for heterochiral dipeptides D-Ile-L-Xaa (Xaa = Ala, Val, or Ile).

	χ_1 (°) (N-C_α-C_β-$C_{\gamma1}$)	χ_2 (°) (C_α-C_β-$C_{\gamma1}$-$C_{\delta1}$)	χ_1 (°) (N-C_α-C_β-$C_{\gamma1}$)	χ_2 (°) (C_α-C_β-$C_{\gamma1}$-$C_{\delta1}$)
D-Ile-L-Val	167.9	-167.9	-	-
D-Ile-L-Ala	-178.3	-168.6	-	-
D-Ile-L-Ile (molecule 1, conformer 1)	-178.0	-168.0	-62.4	-173.8
D-Ile-L-Ile (molecule 1, conformer 2)	-178.0	-168.0	89.7	178.5
D-Ile-L-Ile (molecule 2)	168.8	-163.8	-168.0	176.8
